# Cytotoxic and Optically Active Pyrisulfoxins From the Endophytic *Streptomyces albolongus* EA12432

**DOI:** 10.3389/fchem.2020.00248

**Published:** 2020-05-06

**Authors:** Yuqi Du, Chen Wang, Guodong Cui, Yiwen Chu, Qian Jia, Yi Wang, Weiming Zhu

**Affiliations:** ^1^Key Laboratory of Marine Drugs, Ministry of Education of China, School of Medicine and Pharmacy, Ocean University of China, Qingdao, China; ^2^Sichuan Industrial Institute of Antibiotics, Sichuan Industrial Institute of Antibiotics, Chengdu University, Chengdu, China; ^3^Laboratory for Marine Drugs and Bioproducts of Pilot National Laboratory for Marine Science and Technology, Qingdao, China

**Keywords:** 2, 2′-bipyridine, cytotoxicity, endophyte, *Streptomyces albolongus*, *Aconitum carmichaeli*

## Abstract

*R*-Pyrisulfoxin C (**1**), *S*-pyrisulfoxin D [(+)-**2**], *R*-pyrisulfoxin D [(–)-**2**], pyrisulfoxin E (**13**), *S*-pyrisulfoxin F [(+)-**14**], and *R*-pyrisulfoxin F [(–)-**14**], six new caerulomycin derivatives with a 2,2′-bipyridine skeleton, were obtained from the cultures of the endophytic *Streptomyces albolongus* EA12432 with *Aconitum carmichaeli* (Ranunculaceae). Additionally, the racemic pyrisulfoxins A [(±)-**3**] and B [(±)-**4**] were further purified as optically pure compounds and identified the configurations for the first time. The racemic pyrisulfoxin D [(±)-**2**] displayed significant cytotoxicity against a series of cancer cell lines with IC_50_ values ranging from 0.92 to 9.71 μM. Compounds **7**, **8**, and (±)-**3** showed cytotoxicity against the HCT-116, HT-29, BXPC-3, P6C, and MCF-7 cell lines. Notably, compounds **7** and **8** have a strong inhibition both on the proliferation of human colon cancer cells HCT-116 and HT-29 with IC_50_ values ranging from 0.048 to 0.2 μM (doxorubicin, 0.21 and 0.16 μM), and compound **1** showed a selective inhibition on the proliferation of the gastric carcinoma cell lines, N87, with an IC_50_ value of 8.09 μM. Optically pure compounds *R*(–)-**14** and *S*(+)-**14** showed weak cytotoxicity against HCT-116 and MCF-7 cell lines with the IC_50_ values of 14.7 μM and 10.4 μM, respectively. Interestingly, compounds **1** and (±)-**2** didn't show cytotoxic activity against two human normal cell lines, HEK-293F and L02, with IC_50_ values >100 μM.

**Graphical Abstract:**
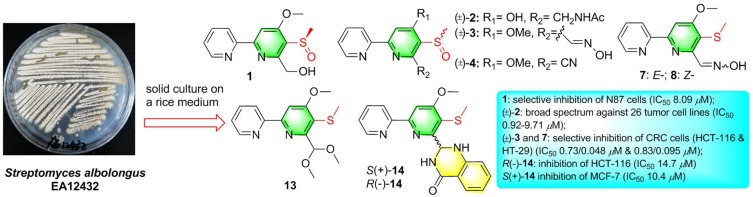
Six new caerulomycin derivatives were obtained from the endophytic Streptomyces albolongus EA12432 by solid culture.

## Introduction

Caerulomycin A is an alkaloid with a 2,2′-bypyridine core, firstly discovered from the cultures of strain *Streptomyces caeruleus* in 1959 (Funk and Divekar, 1959). Since then, large amounts of its analogs or derivatives have been isolated from both wild and mutant actinomycetes, such as caerulomycins (CAEs) (Gomi et al., 1994), collimycins (COLs) (Shindo et al., 1994), and pyrisulfoxins (PYRs) (Tsuge et al., 1999). All of these compounds consist of a 2-substituted pyridine and a tri- or tetra-substituted pyridine ring system, which were also called 2,2′-bipyridine derivatives. 2,2′-Bipyridine derivatives are well-known for their antibacterial (Ambavane et al., 2014; Bu et al., 2014), immunosuppressant (Gurram et al., 2014; Kujur et al., 2015, 2017), and cytotoxic activities (Fu et al., 2011a, 2014; Mei et al., 2019). Our group has reported cytotoxic CAE compounds, cyanogrisides A–N (Fu et al., 2011a, 2014; Mei et al., 2019), CAEs F–K (Fu et al., 2011b), and CAEs T–W (Mei et al., 2019) against several tumor cells, from *Actinoalloteichus* *cyanogriseus* WH1-2216-6. In our ongoing research, we identified three new PYRs containing a methyl sulfoxide group, pyrisulfoxin C (**1**), (+)-pyrisulfoxin D [(+)-**2**], and (–)-pyrisulfoxin D [(–)-**2**], along with the known analogs **3**–**11** and their biosynthetic precursor, picolinic acid (**12**) (Tables S1, S2) (Mehler, 1956), from a rice culture of the endophytic *Streptomyces albolongus* EA12432 isolated from *Aconitum carmichaeli* (Ranunculaceae), a famous Chinese medicinal plant (Yin et al., 2016). When the culture time was extended to 90 d, apart from the isolated compounds **1** and **3**–**11** from 30 d cultures, three new different PYRs, pyrisulfoxin E (**13**), (+)-pyrisulfoxin F [(+)-**14**], and (–)-pyrisulfoxin F [(–)-**14**], were identified (Figure S53). The known analogs included (±)-pyrisulfoxins A [(±)-**3**] and B [(±)-**4**] (Tsuge et al., 1999; Lee et al., 2017), which were further chirally resolved as their optically pure isomers for the first time, *N*-[(4-hydroxy-5-methylthio-2,2′-bipyridin-6-yl)methyl] acetamide (**5**) (Tables S1, S2) (Ignacio et al., 2012) and SF2738 A–F (**6**–**11**) (Tables S1–S3) (Gomi et al., 1994; Yin et al., 2016). The absolute configurations of pyrisulfoxin C (**1**), *S*(+)-**2**, *S*(+)-**3**, *S*(+)-**4**, *R*(–)-**2**, *R*(–)-**3**, and *R*(–)-**4** were determined by experimental and calculated electronic circular dichroism (ECD) spectra. Compounds **1**, (±)-**2**, (±)-**3**, **7**, and **8** displayed significant cytotoxicity against cancer cells with the IC_50_ values ranging from 0.048 to 9.71 μM. Compounds *R*(–)-**14** and *S*(+)-**14** showed inhibitory activity against HCT-116 and MCF-7 cancer cell lines with the IC_50_ values of 14.7 and 10.4 μM, respectively.

Notably, compounds **1**–**4** all bare a methyl sulfoxide group in the 2,2′-bypyridine nuclei. By searching the key words of “natural products (NPs) with sulfoxide” in SciFinder database, about 136 NPs baring sulfoxide group were reported. Apart from numerous sulfoxide-containing NPs from allium plants (Nohara et al., 2012, 2013; Edmands et al., 2013; Radulović et al., 2015; Fukaya et al., 2017), some sulfoxide-containing peptides and β-carboline derivatives, were isolated from marine sponge and ascidian, respectively. The methyl sulfoxide-containing peptides include cytotoxic haligramides A and B (Rashid et al., 2000), hymenamide F (Kobayashi et al., 1996), ciliatamide D (Imae et al., 2013; Takada et al., 2017), waiakeamide (Mau et al., 1996) and its sulfone derivative (Sera et al., 2003), as well as polytheonamides A and B (Hamada et al., 2005). The methyl sulfoxide-containing β-carboline derivatives include eudistomin E (Murata et al., 1991), didemnolines C and D (Schumacher and Davidson, 1995), and eudistomin K sulfoxide with antiviral activity (Lake et al., 1988). Also, a series of sulfoxides with potent cytotoxicity were isolated from microorganisms, including leinamycin (Kara et al., 1989), apratoxin A sulfoxide (Thornburg et al., 2013), and quinomycin derivatives RK-1355 A and B (Lim et al., 2014).

## Materials and Methods

### General Experimental Procedures

Melting points were obtained on X-4 digital display micro-melting point measuring instrument. Optical rotations were measured with POLAX-L polarimeter. Ultraviolet (UV) spectra were recorded on a Thermo Fisher Scientific NanoDrop One micro-spectrophotometer. IR spectra were taken on a Nicolet Nexus 470 spectrophotometer using KBr pellets. ECD spectra were measured on a JASCO-815 spectrometer (JASCO, Tokyo, Japan). Nuclear magnetic resonance (NMR) spectra of compounds **1**, **2**, and **4**–**13** were recorded on a Bruker Avance 500 MHz spectrometer while **2a**, **3**, and **14**, were measured on a JEOL JNM-ECP 600 spectrometer with TMS as an internal standard. Chemical shift (δ) was expressed in ppm with reference to the solvent signals. Mass spectra were recorded on an Agilent 6200 Q-TOF MS system. Thin layer chromatography (TLC) was performed on plates precoated with silica gel GF_254_ (10–40 μm). Column chromatography (CC) was performed on silica gel (100–200 mesh, 200–300 mesh, 300–400 mesh, Qingdao Marine Chemical Ltd., Qingdao, People's Republic of China), RP-18 gel (20–45 μm), and Sephadex LH-20 (Amersham Biosciences). Medium-pressure liquid chromatography (MPLC) was performed on a LC3000 equipped with a P3000A pump modules, and columns packed with RP-18 gel. Semi-preparative high-performance liquid chromatography (HPLC) was performed using an octadecyl silica (ODS) column [YMC-pack ODS-A, 10 × 250 mm, 5 μm, 4 ml/min].

### Actinobacterial Material

The endophytic actinobacterium strain EA12432 was isolated from *Aconitum carmichaeli* (Ranunculaceae) and identified as *Streptomyces albolongus* by 16S rRNA gene sequence and morphological characteristics (Yin et al., 2016).

### Fermentation and Extraction

Spores were inoculated into 500 ml Erlenmeyer flasks containing 150 ml liquid medium that was prepared by dissolving soluble starch (20 g), KNO_3_ (1 g), K_2_HPO_4_·3H_2_O (0.5 g), MgSO_4_·7H_2_O (0.5 g), FeSO_4_ (0.01 g), and NaCl (0.5 g) in sea water (1L). The flasks were incubated at 180 rpm and 28°C for 5 days as seed culture (OD_600_ 1.375), which was then inoculated into 200 × 1,000 ml Erlenmeyer flasks, each containing 80 g rice and 40 ml sea water. All the media were statically cultured at 28°C for 30 d. The culture broth was extracted with ethyl acetate (EtOAc) four times (30 L each). The EtOAc extracts were concentrated under reduced pressure to yield a dark brown gum 30 (40.2 g).

Spores were inoculated into 500 ml Erlenmeyer flasks containing 150 ml liquid medium that was prepared by dissolving soluble starch (20 g), KNO_3_ (1 g), K_2_HPO_4_·3H_2_O (0.5 g), MgSO_4_·7H_2_O (0.5 g), FeSO_4_ (0.01 g), and NaCl (0.5 g) in sea water (1L). The flasks were incubated at 180 rpm and 28°C for 5 days as seed culture (OD_600_ 1.504), which was then inoculated into 1,000 ml Erlenmeyer flasks containing 80 g rice and 40 ml sea water. The flasks were incubated at room temperature for 90 d. The culture broth was soaked and extracted with ethyl acetate (EtOAc) four times (30 L each). The EtOAc extracts were concentrated under reduced pressure to yield a dark brown gum 90 (10 g).

### Isolation

The gum 30 (40.2 g) was separated on silica gel using stepwise gradient elution with ethyl acetate/petroleum ether (0–100%) followed by MeOH/CH_2_Cl_2_ (0–10%) to yield 12 fractions (30 Fr.1–30 Fr.12). 30 Fr.8 (4.46 g) was separated by column chromatography on silica gel using stepwise gradient elution with CH_2_Cl_2_/MeOH (300:1–1:1) to yield eight fractions (30 Fr.8-1–30 Fr.8-8) and **7** (97 mg). 30 Fr.8-1 (818.8 mg) was further separated into three subfractions on Sephadex LH-20, eluting with CH_2_Cl_2_/MeOH (1:1). 30 Fr.8-1-2 (539 mg) was separated on an ODS column eluting with MeOH/H_2_O (10–100%) to give **8** (17.4 mg) and **9** (90.6 mg). 30 Fr.8-1-3 (86.8 mg) was subjected to an ODS column eluting with MeOH/H_2_O (10–100%) to give compound **12** (57.1 mg). 30 Fr.8-8 (60.7 mg) was purified by semi-preparative HPLC on an ODS column (65% MeOH/H_2_O) to yield **10** (32.6 mg, *t*_R_ 11.5 min) and **11** (14.2 mg, *t*_R_ 16.5 min). Fr.10 (2.54 g) was subjected to Sephadex LH-20 using MeOH to afford six subfractions (30 Fr.10-1–30 Fr.10-6). 30 Fr.10-5 (958.9 mg) was further separated by VLC on silica gel eluting with petroleum ether/ethyl acetate (10:1–1:1). 30 Fr.10-5-7 (266.2 mg) was further separated into six subfractions (30 Fr.10-5-7-1–30 Fr.10-5-7-6) on silica gel using stepwise gradient elution with petroleum ether/EtOAc (3:1–1:1). 30 Fr.10-5-7-2 (182 mg) was separated on Sephadex LH-20, eluting with CH_2_Cl_2_/MeOH (1:1), among which the second subfraction, 30 Fr.10-5-7-2-2 (70.3 mg) was purified by a preparative MPLC over an ODS column (25% MeCN/H_2_O) to yield **1** (48.3 mg, *t*_R_ 4.0 min), (±)-**4** (2.1 mg, *t*_R_ 8.5 min), and **6** (7.2 mg, *t*_R_ 12.5 min). (±)-**4** was further separated into (+)-**4** (0.9 mg, *t*_R_ 10.8 min) and (–)-**4** (1.7 mg, *t*_R_ 12.0 min) on a ChiralPak IA analytical column (40% EtOH/*n*-hexane). 30 Fr.10-5-8 (700 mg) were separated on Sephadex LH-20, eluting with CH_2_Cl_2_/MeOH (1:1). Fr.10-5-8-2 (450 mg) was separated into 10 subfractions (30 Fr.10-5-8-2-1–30 Fr.10-5-8-2-10) on silica gel using stepwise gradient elution with CH_2_Cl_2_/MeOH (40:1–1:1). 30 Fr.10-5-8-2-4 (151.7 mg) was then purified by semi-preparative HPLC (20% MeCN/H_2_O) to yield (±)-**3** (6.5 mg, *t*_R_ 23.0 min), which was further separated into (+)-**3** (2.1 mg, *t*_R_ 8.0 min) and (–)-**3** (1.6 mg, *t*_R_ 9.5 min) on a ChiralPak IA analytical column (35% EtOH/*n*-hexane). 30 Fr.10-5-8-2-5 (85.1 mg) was purified by MPLC (20% MeCN/H_2_O, ODS-A C18 column) to yield (±)-**2** (10 mg, *t*_R_ 4.5 min), along with **5** (25.1 mg, *t*_R_ 7.8 min). (±)-**2** was further separated into (+)-**2** (1.3 mg, *t*_R_ 21.0 min) and (–)-**2** (1.7 mg, *t*_R_ 24.0 min) on a Chiral tris(3,5-dimethylphenylcarbamate) immobilized cellulose (INB) analytical column (20% EtOH/*n*-hexane/Et_2_NH).

The gum 90 (10 g) was subjected to a silica gel column, eluted by stepwise gradient of ethyl acetate/petroleum ether (0–100%) followed by MeOH/CH_2_Cl_2_ (0–10%) to yield 13 fractions (90 Fr.1–90 Fr.13). 90 Fr.11 (300 mg) was separated on a Sephadex LH-20 column eluted by MeOH to give five subfractions, 90 Fr.11-1–90 Fr.11-5. 90 Fr.11-2 (40 mg) was further separated by semi-preparative HPLC (65% MeOH/H_2_O, YMC-ODS column) to yield compound **13** (2.5 mg, *t*_R_ 9.0 min). 90 Fr.12 (400 mg) was separated on a Sephadex LH-20 column eluted by MeOH to provide three subfractions, 90 Fr.12-1–90 Fr.12-3. 90 Fr.12-2 (100 mg) was further separated by semi-preparative HPLC (60% MeOH/H_2_O, YMC-ODS column) to yield (±)-**14** (3.0 mg, *t*_R_ 8.0 min), which was resolved into (+)-**14** (1.2 mg, *t*_R_ 22.0 min) and (–)-**14** (1.1 mg, *t*_R_ 16.0 min) on a ChiralPak IA analytical column (30% *i*-PrOH/*n*-hexane).

*R*-Pyrisulfoxin C (**1**): white amorphous powder; ${\left[\alpha \right]}_{\text{D}}^{13}$ −33.4 (*c* 0.5, MeOH); UV(MeOH) λ_max_ (log ε) 249 (3.57), 289 (3.81) nm; IR (KBr) ν_max_ 3,346, 2,922, 1,569, 1,464, 1,422, 1,374, 1,039, 957, 798 cm^−1^; ECD (0.90 *m*M, MeOH) λ_max_ (Δε) 216 (+5.5), 243 (+2.9), and 291 (−3.9) nm; ^1^H NMR and ^13^C NMR, see Tables 1, 3. High Resolution Electrospray Ionization Mass Spectroscopy (HRESIMS) *m*/*z* 279.0795 [M + H]^+^ (calcd for C_13_H_15_N_2_O_3_S, 279.0798).

**Table 1:** ^1^H Nuclear Magnetic Resonance (NMR) data of compounds **1**, (±)-**2**, and (±)-**2a** at 500 MHz.

**No**.	**1 (CD_**3**_OD)**	**(±)-2 (DMSO-*d*_**6**_)**	**(±)-2 (CD_**3**_OD)**	**(±)-2*^a^* (DMSO-*d*_**6**_)**	**(±)-2a*^b^* (DMSO-*d*_**6**_)**
	***δ***_**H**_ **(*****J*** **in Hz)**	***δ***_**H**_ **(*****J*** **in Hz)**	***δ***_**H**_ **(*****J*** **in Hz)**	***δ***_**H**_ **(*****J*** **in Hz)**	***δ***_**H**_ **(*****J*** **in Hz)**
3	8.01, s	7.38, s	7.15, s	7.36, s	8.03, s
4	–	–	–	–	–
4-OCH_3_	4.05, s	–	–	–	4.04, s
5	–	–	–	–	–
5-SOCH_3_	3.06, s	3.03, s	3.16, s	3.01, s	3.03, s
6	–	–	–	–	–
7	4.96, d (15.0) 4.92, d (15.0)	4.71, m	4.91, m	4.72, m	4.65, dd (5.3, 15.8) 4.85, dd (6.0, 15.8)
NHCOCH_3_	–	1.92, s	2.07, s	1.92, s	1.92, s
3′	8.46, dd (7.7)	8.32, d (7.8)	8.14, d (7.7)	8.31, d (7.1)	8.52, d (7.7)
4′	7.92, dd (7.7, 7.5)	7.96, dd (7.8, 7.5)	7.99, dd (7.7, 7.0)	7.98, dd (7.1, 6.0)	8.03, m
5′	7.42, dd (7.5, 4.4)	7.49, dd (7.5, 4.0)	7.54, dd (7.0, 4.0)	7.50, brs	7.53, dd (7.5, 5.0)
6′	8.64, d (4.4)	8.69, d (4.0)	8.75, d (4.0)	8.70, brs	8.73, brs
NH	–	8.49, brs	–	8.46, brs	8.33, brs

a *Synthetic compound*;

b *Recorded at 600 MHz*.

*S*-Pyrisulfoxin D [(+)-**2**]: white amorphous powder; ${\left[\alpha \right]}_{\text{D}}^{13}$ +14.2 (*c* 0.5, MeOH); UV(MeOH) λ_max_ (log ε) 242 (4.49), 282 (4.28) nm; IR (KBr) ν_max_ 3,260, 3,053, 2,922, 1,631, 1,572, 1,493, 1,383, 1,290, 1,029, 794 cm^−1^; ECD (0.82 *m*M, MeOH) λ_max_ (Δε) 221 (−12.8), 250 (+9.4), and 300 (+5.3) nm; ^1^H NMR and ^13^C NMR, see Tables 1, 3. HRESIMS *m*/*z* 306.0916 [M + H]^+^ (calcd for C_14_H_16_N_3_O_3_S, 306.0907).

*R*-Pyrisulfoxin D [(–)-**2**]: white amorphous powder; ${\left[\alpha \right]}_{\text{D}}^{13}$ −16.6 (*c* 0.5, MeOH); UV(MeOH) λ_max_ (log ε) 242 (4.49), 282 (4.28) nm; IR (KBr) ν_max_ 3,260, 3,053, 2,922, 1,631, 1,572, 1,493, 1,383, 1,290, 1,029, 794 cm^−1^; ECD (0.82 *m*M, MeOH) λ_max_ (Δε) 220 (+9.7), 250 (−7.7), and 300 (−3.4) nm; ^1^H NMR and ^13^C NMR, see Tables 1, 3. HRESIMS *m*/*z* 306.0916 [M + H]^+^ (calcd for C_14_H_16_N_3_O_3_S, 306.0907).

*S*-Pyrisulfoxin A [(+)-**3**]: white amorphous powder; ${\left[\alpha \right]}_{\text{D}}^{15}$ +51.5 (*c* 1, MeOH); UV(MeOH) λ_max_ (lg ε) 243 (4.37), 287 (4.13) nm; ECD (0.86 *m*M, MeOH) λ_max_ (Δε) 207 (−17.2), 244 (−4.6), and 296 (+5.4) nm; ^1^H NMR and ^13^C NMR, see Tables 2, 3, Figures S33, S34. HRESIMS *m*/*z* 292.0754 [M + H]^+^ (calcd for C_13_H_14_N_3_O_3_S, 292.0750) (Figure S31).

**Table 2:** ^1^H NMR data of compounds (±)-**3**, (±)-**4**, **13**, and (±)-**14** at 500 MHz.

**No**.	**(±)-3 *^***a***^* (CD_**3**_OD)**	**(±)-4 (CDCl_**3**_)**	**13 (CDCl_**3**_)**	**(±)-14 ***^a^^,^^b^*** (CDCl_**3**_)**
	***δ***_**H**_ **(*****J*** **in Hz)**	***δ***_**H**_ **(*****J*** **in Hz)**	***δ***_**H**_ **(*****J*** **in Hz)**	***δ***_**H**_ **(*****J*** **in Hz)**
3	8.14, s	8.31, s	8.01, s	8.05, s
4	–	–	–	–
4-OCH_3_	4.15, s	4.17, s	4.10, s	4.12, s
5	–	–	–	–
5-SO/S-CH_3_	3.15, s	3.11, s	2.38, s	2.44, s
6	–	–	–	–
7	8.44, s	–	6.09, s	6.36, s
7-OCH_3_	–	–	3.56, s	3.56, s
NHCOCH_3_	–	–	–	–
3′	8.49, d (8.0)	8.52, dd (8.0, 1.2)	8.56, d (8.0)	8.33, dd (7.8, 1.5)
4′	7.95, dd (8.0, 7.8)	7.89, ddd (8.0, 7.8, 1.8)	7.80, dd (8.0, 7.8)	7.80, ddd (7.8, 7.8, 1.5)
5′	7.49, dd (7.8, 5.0)	7.43, ddd (7.8, 4.6, 1.2)	7.31, dd (7.8, 4.8)	7.30, m (overlap)
6′	8.67, brs	8.68, dd (4.6 1.8)	8.65, d (4.8)	8.62, dd (4.5, 1.5)

a *Recorded at 600 MHz*;

b *The δ_H_ values of H-8, H-12, and H-13–H-16 were 6.82 (brs, H-8), 5.22 (brs, H-12), 6.78 (dd, J = 7.5, 1.2 Hz, H-13), 7.32 (m, overlap, H-14), 6.90 (ddd, J = 7.9, 7.5, 1.2 Hz, H-15), and 7.96 (dd, J = 7.9, 1.1 Hz, H-16), respectively*.

**Table 3:** ^13^C NMR data of compounds **1**–**4**, (±)-**2a**, **13**, and (±)-**14** at 125 MHz.

**No**.	**1 (CD_**3**_OD)**	**(±)-2 (DMSO-*d*_**6**_)**	**(±)-2 (CD_**3**_OD)**	**(±)-2*^a^* (DMSO-*d*_**6**_)**	**(±)-2a*^b^* (DMSO-*d*_**6**_)**	**(±)-3*^b^* (CD_**3**_OD)**	**(±)-4*^a^* (CDCl_**3**_)**	**13 (CDCl_**3**_)**	**(±)-14*^b^^,^^c^* (CDCl_**3**_)**
	**δ_C_**	**δ_C_**	**δ_C_**	**δ_C_**	**δ_C_**	**δ_C_**	**δ_C_**	**δ_C_**	**δ_C_**
2	160.2, C	Absent	Absent	Absent	158.3, C	160.9, C	161.7, C	absent	158.0, C
3	105.2, CH	111.3, C^d^	114.1, C	111.1, C	103.6, CH	105.6, CH	107.0, CH	103.4, CH	103.8, CH
4	166.9, C	Absent	Absent	Absent	164.9, C	167.8, C	165.1, C	168.1, C*^d^*	167.9, C
4-OCH_3_	57.1, CH_3_	–	–	–	57.2, CH_3_	57.1, CH_3_	57.4, CH_3_	56.4, CH_3_	56.7, CH_3_
5	126.0, C	125.3, C^d^	127.1, C^d^	Absent	126.5, C	127.5, C	132.2, C	120.7, C*^d^*	118.6, C
5-SO/S-CH_3_	38.8, CH_3_	38.6, CH_3_	39.0, CH_3_	38.9, CH_3_	38.9, CH_3_	38.5, CH_3_	40.4, CH_3_	18.3, CH_3_	18.2, CH_3_
6	161.0, C	154.8, C^d^	absent	Absent	157.4, C	152.9, C	133.3, C	absent	156.6, C
7	64.6, CH_2_	41.4, ${\text{CH}}_{2}^{\text{d}}$	37.7, ${\text{CH}}_{2}^{\text{d}}$	Absent	41.5, CH_2_	148.3, CH	114.9, C	101.7, CH	65.7, CH
NHCOCH_3_	–	22.5, CH_3_ 170.4, C^d^	22.3, CH_3_ 174.9, C^d^	22.4, CH_3_ 170.5, C	23.4, CH_3_ 169.9, C	–	–	–	–
7-OCH_3_	–	–	–	–	–	–	–	54.8, CH_3_	
2′	155.6, C	absent	absent	absent	154.5, C	155.6, C	153.3, C	155.7, C*^d^*	154.9, C
3′	123.1, CH	121.1, CH	122.3, CH	121.1, CH	121.7, CH	123.2, CH	122.6, CH	122.2, CH	121.9, CH
4′	138.7, CH	137.6, CH	139.2, CH	137.7, CH	138.2, CH	138.8, CH	137.8, CH	137.1, CH	137.3, CH
5′	126.2, CH	125.0, CH	126.8, CH	125.1, CH	125.5, CH	126.3, CH	126.0, CH	124.2, CH	124.6, CH
6′	150.3, CH	149.3, CH	150.7, CH	149.3, CH	149.8, CH	150.3, CH	149.7, CH	149.0, CH	149.1, CH

a *Synthetic compound*;

b *Recorded at 150 MHz*;

c *The δ_C_ values of C-9–C-11 and C-13–C-16 were 165.2 (C, C-9), 117.4 (C, C-10), 147.2 (C, C-11), 115.6 (CH, C-13), 133.8 (CH, C-14), 119.9 (CH, C-15), and 128.6 (CH, C-16), respectively*;

d *Absent in 1D NMR but present in 2D NMR*.

*R*-Pyrisulfoxin A [(–)-**3**]: white amorphous powder; ${\left[\alpha \right]}_{\text{D}}^{15}$ −40.7 (*c* 1, MeOH); UV(MeOH) λ_max_ (lg ε) 243 (4.37), 287 (4.13) nm; ECD (0.86 *m*M, MeOH) λ_max_ (Δε) 207 (+11.7), 244 (+3.1), and 296 (−3.6) nm; ^1^H NMR and ^13^C NMR, see Tables 2, 3, Figures S33, S34. HRESIMS *m*/*z* 292.0752 [M + H]^+^ (calcd for C_13_H_14_N_3_O_3_S, 292.0750) (Figure S32).

*S*-Pyrisulfoxin B [(+)-**4**]: white amorphous powder; ${\left[\alpha \right]}_{\text{D}}^{14}$ +22.4 (*c* 0.25, MeOH); UV(MeOH) λ_max_ (lg ε) 240 (3.58), 289 (3.47) nm; ECD (0.92 *m*M, MeOH) λ_max_ (Δε) 214 (−2.9), 243 (−2.1) and 293 (+1.8) nm; ^1^H NMR and ^13^C NMR, see Tables 2, 3, Figures S37, S38. HRESIMS *m*/*z* 274.0650 [M + H]^+^ (calcd for C_13_H_12_N_3_O_2_S, 274.0645) (Figure S35).

*R*-Pyrisulfoxin B [(–)-**4**]: white amorphous powder; ${\left[\alpha \right]}_{\text{D}}^{14}$ −15.2 (*c* 0.25, MeOH); UV(MeOH) λ_max_ (lg ε) 240 (3.58), 289 (3.47) nm; ECD (0.92 *m*M, MeOH) λ_max_ (Δε) 214 (+5.3), 243 (+4.0) and 293 (−3.6) nm; ^1^H NMR and ^13^C NMR, see Tables 2, 3, Figures S37, S38. HRESIMS *m*/*z* 274.0650 [M + H]^+^ (calcd for C_13_H_12_N_3_O_2_S, 274.0645) (Figure S36).

Pyrisulfoxin E (**13**): white amorphous powder; UV(MeOH) λ_max_ (log ε) 226 (3.49), 287 (3.36) nm; ^1^H NMR and ^13^C NMR, see Tables 2, 3. HRESIMS *m*/*z* 307.1118 [M + H]^+^ (calcd for C_15_H_19_N_2_O_3_S, 307.1111).

*S*-Pyrisulfoxin F [(+)-**14**]: white amorphous powder; ${\left[\alpha \right]}_{\text{D}}^{25}$ +14.5 (*c* 0.5, MeOH); UV(MeOH) λ_max_ (log ε) 221 (2.49), 282 (2.15) nm; ECD (1.3 *m*M, MeOH) λ_max_ (Δε) 214 (+3.14), 230 (–2.42), 251 (–2.72), 269 (+0.09), 290 (–2.51), and 325 (+1.31) nm; ^1^H NMR and ^13^C NMR, see Tables 2, 3. HRESIMS *m*/*z* 379.1222 [M + H]^+^ (calcd for C_20_H_19_N_4_O_2_S, 379.1223).

*R*-Pyrisulfoxin F [(–)-**14**]: white amorphous powder; ${\left[\alpha \right]}_{\text{D}}^{25}$ −16.9 (*c* 0.5, MeOH); UV(MeOH) λ_max_ (log ε) 221 (2.49), 282 (2.15) nm; ECD (1.3 *m*M, MeOH) λ_max_ (Δε) 212 (–2.81), 230 (+1.48), 251 (+1.66), 269 (–0.18), 290 (+1.77), and 325 (–0.95) nm; ^1^H NMR and ^13^C NMR, see Tables 2, 3. HRESIMS *m*/*z* 379.1222 [M + H]^+^ (calcd for C_20_H_19_N_4_O_2_S, 379.1223).

### Oxidation of Compound 5 to (±)-2

Compound **5 (**9.0 mg) was dissolved into 2.25 ml of THF and 0.45 ml of water. The mixture was cooled in an ice bath, and 120 μl of aqueous solution of potassium peroxomonosulfate (Oxone) (0.1 g/ml) was added dropwise. The resulting mixture was stirred at 0°C for 2.5 h. Then, 1 ml of aqueous solution of NaHCO_3_ (pH 8) was added to quench the reaction. The reaction product was extracted by *n*-BuOH to afford 9.2 mg (96.8% yield) of (±)-**2**, which was identified by the same MS (Figure S22) and co-HPLC retention time (*t*_R_ 10.5 min, Figure S39) and NMR (Figures S23, S24, Tables 1, 3) to those of natural one.

### Methylation of (±)-2 With TMS-CHN_2_

Five milligram of (±)-**2** was dissolved in anhydrous MeOH (5 ml), and then 0.9 ml of TMS-CHN_2_ (2.0 M in *n*-hexane) was added. After stirring for 2.5 h at room temperature (about 20°C), the reaction mixture was evaporated to dryness and prepared by semi-preparative HPLC on an ODS column (10%−100% MeCN-H_2_O with 0.5%0 CF_3_CO_2_H) to yield (±)-**2a** (2.5 mg, *t*_*R*_ 5.36 min, 43.5% yield). (±)-**2a** was further separated into (+)-**2a** (1.0 mg, *t*_R_ 10.8 min) and (–)-**2a** (1.0 mg, *t*_R_ 14.0 min) on a Chiral INC 5 u analytical column (35% EtOH/*n*-hexane).

4-*O*-Methylpyrisulfoxin D [(±)-**2a**]: white amorphous powder; UV(MeOH) λ_max_ (log ε) 221 (3.05), 248 (2.81), 289 (3.00) nm; IR (KBr) ν_max_ 3,444, 1,683, 1,574, 1,428, 1,383, 1,205, 1,136, 1,056, 800 cm^−1^; ^1^H NMR (600 MHz) and ^13^C NMR (150 MHz), see Tables 1, 3, Figures S26–S30. HRESIMS *m*/*z* 320.1070 [M + H]^+^ (calcd for C_15_H_18_N_3_O_3_S, 320.1063) (Figure S25).

*S*-4-*O*-Methylpyrisulfoxin D [(+)-**2a**]: ${\left[\alpha \right]}_{\text{D}}^{25}$ +44.6 (*c* 0.5, MeOH); ECD (1.57 *m*M, MeOH) λ_max_ (Δε) 217 (−7.8), 245 (−3.8) and 293 (+6.1) nm.

*R*-4-*O*-Methylpyrisulfoxin D [(–)-**2a**]: ${\left[\alpha \right]}_{\text{D}}^{25}$ −41.4 (*c* 0.5, MeOH); ECD (1.57 *m*M, MeOH) λ_max_ (Δε) 217 (+6.2), 245 (+2.9) and 293 (−4.8) nm.

### ECD Calculation

The calculations were performed by using the density functional theory (DFT) as carried out in the Gaussian 09 (Frisch et al., 2010). The preliminary conformational distributions search was performed by HyperChem 8.0 software. All ground-state geometries were optimized at the B3LYP/6-31G(d) level (Stephens et al., 2007) (Tables S4–S6). Time-dependent DFT (TDDFT) at B3LYP/6-31G(d) was employed to calculate the electronic excitation energies and rotational strengths in MeOH (Casida, 1995) (Tables S7–S9). The overall calculated ECD curves were weighted by Boltzmann distribution with a half-bandwidth of 0.30 eV and UV corrections of compounds (*S*)-**2**, (*R*)-**3**, and (*S*)-**14** were −15, 0, and 0 nm, respectively. The calculated ECD spectra were produced by SpecDis 1.70.1 software (Bruhn et al., 2017). Solvent effects of MeOH were evaluated at the same DFT level by use of the SCRF/PCM method (Cammi and Tomasi, 1995).

### Cytotoxic Assays

The cytotoxic activities of compounds **1** and (±)-**2** were detected by CellTiter-Glo® (CTG) assay (He et al., 2019) against human colon carcinoma cell lines (HCT-116), lung cancer cell lines (A549, H1975, H1299, SPC-A1, H2228), breast cancer cell lines (MCF-7, BT474, MDA-MB-231, MDA-MB-468), glioblastoma cell line (U87, U251), leukemia cell lines (HL-60, MV-4-11, K562), ductal carcinoma cell line (HCC1954, HUCCT1), gastric cancer cell line (MKN-45), epidermoid carcinoma cell line (A431), liver cancer cell line (Hep3B), prostate cancer cell line (DU145), gastric carcinoma cell line (N87), rhabdomyoma cell line (A673), bone osteosarcoma cell line (143B), T cell lymphoma cell line (Karpass299), B16F10 (highly metastatic mouse melanoma cell line), as well as the human embryonic kidney-293F cell line (HEK-293F) and normal liver cell line (L02). Cells were cultured in Dulbecco's Modified Eagle Medium (DMEM) supplemented with 10% fetal bovine serum and 1% penicillin–streptomycin solution under a humidified atmosphere of 95% air and 5% CO_2_ at 37°C. Ninety microliter of culture solution (containing fetal bovine serum) and 100 μl of cell suspension at a density of 2 × 10^3^ cell/ml was plated in 96-well microtiter plates, allowed to attach overnight, and then exposed to 10 μl of drugs for 72 h within the final concentrations of 0.032, 0.16, 0.8, 4, 20, and 100 μM, respectively. Hundred microliter of the CTG solution was then added to each well and incubated for 10 min, and the absorbance was read at 500 nm on a Spectra Max Plus plate reader. Adriamycin was used as the positive control.

The cytotoxicities of compounds **3**–**12** against Jurkat, K562, MCF-7, and P6C cell lines were assayed by 3-(4,5-Dimethylthiazol-2-yl)-2,5-diphenyltetrazolium bromide (MTT) method (Mosmann, 1983). MCF-7 and P6C cell lines were grown in RPMI-1640 supplemented with 10% Fetal Bovine Serum (FBS) under a humidified atmosphere of 5% CO_2_ and 95% air at 37°C, respectively. Cell suspension, 200 μl, at a density of 5 × 10^4^ cell/ml was plated in 96-well microtiter plates and incubated for 24 h. Then, the samples to be tested (final concentration, 10 μM) were added to each well and further incubated for 72 h. Twenty microliter of MTT solution (5 mg/ml in IPMI-1640) was then added to each well and incubated for 4 h. Old medium containing MTT (150 μl) was then gently replaced by DMSO and pipetted to dissolve any formazan crystals formed. Absorbance was then determined on a Spectra Max Plus plate reader at 570 nm. Jurkat and K562 cell lines were grown in DMEM supplemented with 10% FBS, 2 mM L-glutamine, 100 U/ml penicillin and 100 g/ml streptomycin under a humidified atmosphere of 5% CO_2_ at 37°C. The cells in the logarithmic growth phase were seeded in 96-well plates at 8,000 cells/well (180 ml/well). After 24 h at 37°C and 5% CO_2_, the samples to be tested were added (final concentration, 10 μM) and three replicate wells were set for each concentration. The solvent control Dimethyl Sulfoxide (DMSO) was used in an amount of 0.1% of the maximum dose used in the test group. After 72 h of drug treatment at 37°C and 5% CO_2_, 20 μl of MTT (5 mg/ml) was added and incubated for another 4 h. Then, 100 μl mixture of 10% SDS, 5% isopropanol and 12 mM HCl was added and incubated for 12–20 h. The optical density of each well at 570 nm was read by a microplate reader.

The cytotoxicities of compounds **3**–**12** against HT29, HCT-116, and BXPC-3 cell lines were assayed by SRB method (Skehan et al., 1990). They were cultured as K562 and Jurkat cell lines described above. The cells in the logarithmic growth phase were seeded in 96-well plates at 8,000 cells/well (180 ml/well). After 24 h in 5% CO_2_ and 37°C, the samples to be tested were added (final concentration, 10 μM) and three replicate wells were set for each concentration. The solvent control DMSO was used in an amount of 0.1% of the maximum dose used in the test group. After 72 h of drug treatment at 37°C and 5% CO_2_, 50% (m/v) ice-cold trichloroacetic acid was added to each well to fix the cells. After SRB staining, 150 μl/well of Tris solution was added and the optical density of each well at 540 nm was read in a microplate reader.

## Results and Discussion

### Identification of Compounds

Pyrisulfoxin C (**1**) was obtained as white amorphous powder. The molecular formula of **1** was determined to be C_13_H_14_N_2_O_3_S by HRESIMS (Figure S5) with one more oxygen atom than compound **9**. Analysis of the ^1^H NMR data (Table 1, Figure S6) for **1** and comparison with reported data showed four signals at 8.46 (H-3′, d, *J* = 7.7 Hz), 7.92 (H-4′, dd, *J* = 7.7, 7.5 Hz), 7.42 (H-5′, dd, *J* = 7.5, 4.4 Hz), and 8.64 (H-6′, d, *J* = 4.4 Hz), which were assigned to a 2-substituted pyridine ring system, and one signal at 8.01 (H-3, s) assigned to a 2,4,5,6-tetrasubstituted pyridine ring system. Heteronuclear Multiple Bond Correlations (HMBC) (Figure 2, Figure S10) from H-3′ to C-2 and from H-3 to C-2' identified a 2,2′-bipyridine structure. The signal at δ_C/H_ 64.6/4.92&4.96 was assigned as a hydroxymethyl. In addition, two methyl singlets were observed, among which the signal at δ_C/H_ 57.1/4.05 was a methoxy and the one at δ_C/H_ 38.8/3.15 was assigned as a methylsulfinyl group (Tables 1, 3, Figures S7–S9). These data were very similar to those of SF2738 C (**9**) (Yin et al., 2016) except for the methylsulfinyl signal (δ_C/H_ 38.8/3.15), indicating **1** as a methyl sulphoxide (Figure 2). The constitution of **1**, named as pyrisulfoxin C, was thus elucidated as 4-methoxy-5-methylsulfinyl-2,2′-bipyridine-6-methanol.

**Figure 1:**
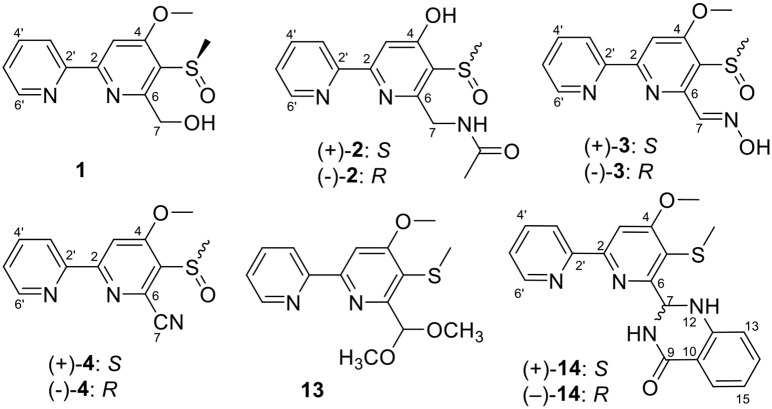
Structures of compounds **1**–**3**, **13**, and **14**.

**Figure 2:**
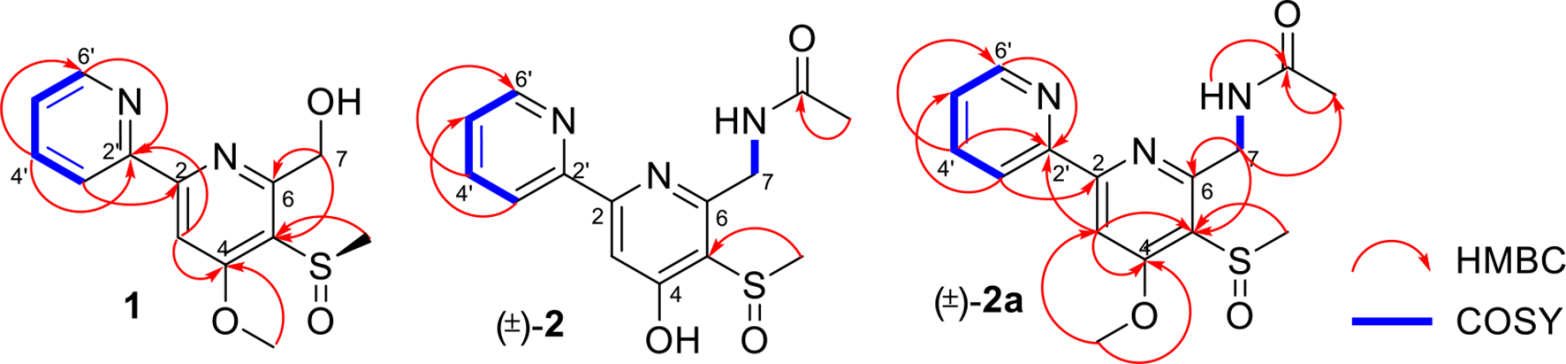
Key ^1^H-^1^H COSY and HMBC correlations of **1**, (±)-**2**, and (±)-**2a**.

(±)-Pyrisulfoxin D [(±)-**2**] was obtained as a pair of racemate. Its molecular formula was assigned as C_14_H_15_N_3_O_3_S from the HRESIMS peak at *m*/*z* 306.0916 [M+H]^+^ (Figure S11), which has one more oxygen atom than compound **5**. Similar to **1**, proton signals measured in DMSO-*d*_6_ at 8.32 (H-3′, d), 7.96 (H-4′, dd), 7.49 (H-5′, dd), 8.69 (H-6′, d), and 7.38 (H-3, s) (Table 1, Figure S12) were assigned to a 2,2′-bipyridine system. Besides the 2,2′-bipyridine core skeletons, its NMR (Tables 1, 3, Figures S12–S14) displayed one NH signal at δ_H_ 8.49 (1H, brs), one methylene at δ_H_ 4.71, and one acetyl group at δ_C/H_ 22.5/1.92 and δ_C_ 170.4. And the key COSY correlation signals (Figure 2, Figure S15) of H-7 (δ_H_ 4.71, m, 2H) to NH (δ_H_ 8.49, brs, 1H) revealed the -CH_2_-NH- group. The HMBC correlation signals (Figure 2, Figure S16) between both methyl protons (δ_H_ 1.92) and methylene protons (δ_H_ 4.71) with the carbonyl carbon (δ_C_ 170.4) established an (acetylamino)methyl group. These data are very similar to those of **5** (Ignacio et al., 2012) within the main difference that the -SCH_3_ protons' signal at δ_H_ 2.25 (3H, s) in **5** moved to δ_H_ 3.03 (3H, s) in (±)-**2**, indicating the existence of a -S(O)CH_3_ group in (±)-**2**. Unexpectedly, three non-protonated carbon signals (C-2, C-4 and C-2′) were absent in both 1D and 2D NMR spectra of (±)-**2**, even after changing the solvent as MeOH-*d*_6_ (Table 3, Figures S17–S21). To further confirm the constitution of (±)-**2**, (±)-**2** was prepared by oxidation of the known compound **5** with Oxone (Scheme 1). The product was identified by the same MS (Figure S22), the same co-HPLC retention times (*t*_R_ 10.5 min, Figure S39) and almost the same 1D NMR (Figures S23, S24, Tables 1, 3) to the natural (±)-**2**. Therefore, the constitution of (±)-**2**, named as pyrisulfoxin D, was identified as *N*-[(4-hydroxy-5-methylsulfinyl-2,2′-bipyridin-6-yl)methyl] acetamide. However, the carbon signals of C-2, C-4, C-5, C-6, C-7, and C-2′ were neither absent in the ^13^C NMR spectrum of the synthetic (±)-**2** due to the coinfluence of 4-OH and 3-S(O)CH_3_. In order to verify this hypothesis, a new 4-*O*-methyl derivative [(±)-**2a**] was prepared from the methylation of (±)-**2** by TMS-CHN_2_ (Scheme 1) and identified by HRESIMS (Figure S25) as well as 1D and 2D NMR spectra (Figure 2, Figures S26–S30). As expected, these non-hydrogenated carbon signals were present in the ^13^C NMR spectrum of (±)-**2a** (Figure S27, Table 3).

**Scheme 1:**
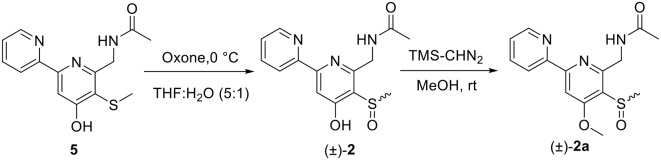
The oxidation of **5** and the methylation of (±)-**2** into (±)-**2a**.

**Figure 3:**
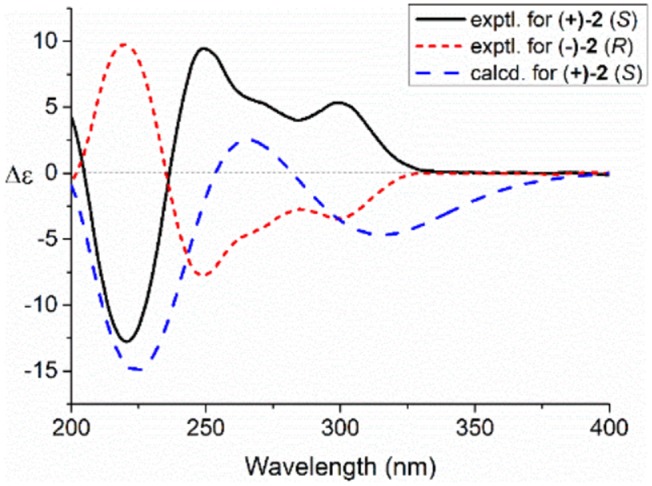
Experimental and calculated electronic circular dichroism (ECD) spectra of *S*(+)-**2** and *R*(–)-**2** in MeOH.

**Figure 4:**
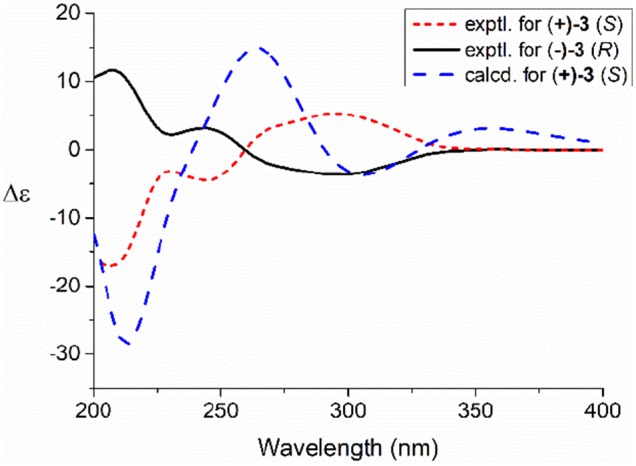
Experimental and calculated ECD spectra of *S*(+)-**3** and *R*(–)-**3** in MeOH.

**Figure 5:**
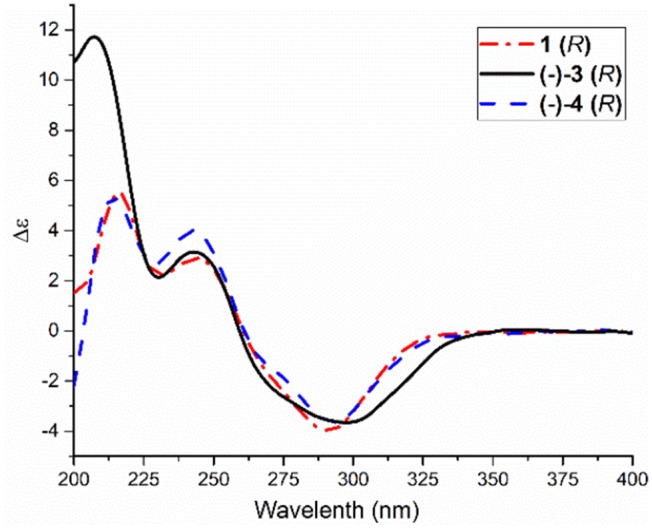
ECD spectra of **1**, *R*(–)-**3** and *R*(–)-**4** in MeOH.

Compared to other natural products, the absolute configuration of the chiral sulfoxide is less concerned. Apart from X-ray single crystal diffraction, sulfoximine NMR method (Kusumi et al., 2006), and vibrational circular dichroism (VCD) spectroscopy (Stephens et al., 2001), the ECD spectroscopy was widely used to determine the absolute configuration of the chiral sulfoxide (Cho and Plapp, 1998; Donnoli et al., 2003). To determine the absolute configurations, the racemic pyrisulfoxin D [(±)-**2**], pyrisulfoxin A [(±)-**3**], pyrisulfoxin B [(±)-**4**] and (±)-4-*O*-methyl pyrisulfoxin D [(±)-**2a**] were first separated on a chiral column into their optically pure isomers (+)-**2** and (–)-**2** (Figure S1), (+)-**3** and (–)-**3** (Figure S2), (+)-**4** and (–)-**4** (Figure S3), as well as (+)-**2a** and (–)-**2a** (Figure S4), respectively. Then, ECD spectra were measured and the results indicated that (+)-**2**, (+)-**2a**, (+)-**3**, and (+)-**4** displayed strong negative Cotton effects, while (–)-**2**, (–)-**2a**, (–)-**3**, and (–)-**4** showed strong positive Cotton effects around λ_max_ 210–220 nm (Figures 3–5). The Cotton effects around λ_max_ 210–220 nm that arose from the exciton-couple of the chromophores S=O (n → π^*^) and pyridine (π → π^*^) could be used to identify the absolute configuration of aryl methyl sulfoxides, which the positive and negative effects were correlated to the *R*- and *S*- configurations, respectively (Cho and Plapp, 1998). To confirm the deduction, we further calculated the ECD spectra of the optically pure compounds (*S*)-**2** and (*R*)-**3** by means of DFT at B3LYP/6-31G(d) level. The calculated ECD spectra (*S*)-**2** and (*R*)-**3** matched well with the experimental ECD spectra of (+)-**2** and (–)-**3**, which showed Cotton effects around λ_max_ (Δε) 221 (−12.8), 250 (+9.4) and 300 (+5.3) nm, and 207 (+11.7), 244 (+3.1) and 296 (−3.6) nm, respectively (Figures 3, 4), further indicating the configurations of (+)-**2** and (–)-**3** as *S*- and *R*-, respectively. Thus, the absolute configurations of (–)-**2** and (+)-**3** were respectively determined as *R*- and *S*-. As shown in Figure 5, the experimental ECD spectra of **1**, (–)-**2a**, and (–)-**4** were similar to that of (–)-**3**, but opposite to those of (+)-**3**, (+)-**2a**, and (+)-**4**. Thus, the absolute configurations of **1**, (–)-**2a**, (–)-**4**, (+)-**2a**, and (+)-**4** were determined to be *R*-, *R-, R-, S*-, and *S*-, respectively.

**Figure 6:**
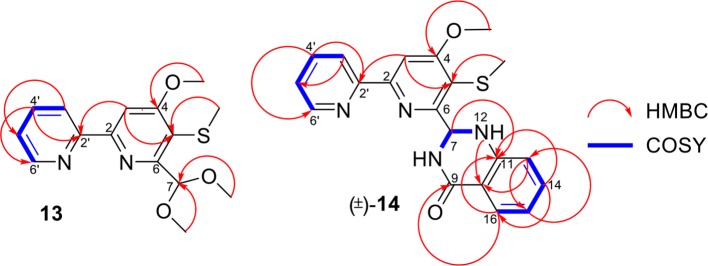
Key ^1^H-^1^H COSY and HMBC correlations of **13** and (±)-**14**.

Pyrisulfoxin E (**13**) was obtained as white amorphous powder. HRESIMS showed the protonated molecular ion peak at *m/z* 307.1118 [M+H]^+^, indicating a molecular formula C_15_H_18_N_2_O_3_S (Figure S40). ^1^H NMR signals at 8.56 (H-3′, d), 7.80 (H-4′, dd), 7.31 (H-5′, dd), 8.65 (H-6′, d) (Table 2, Figure S41), and 8.01 (H-3, s) were assigned to a 4,5,6-tetrasubstituted 2,2′-bipyridine ring system. What more, ^1^H-^1^H COSY correlative signals of H-3′/H-4′/H-5′/H-6′ (Figure S44) and HMBC correlative signals from H-2 to C-2' (Figure S45) verified that. Three methoxy and one methylthio signals were observed at δ_C/H_ 56.4/4.10 (OCH_3_-4), 54.8/3.56 (OCH_3_-7), and δ_C/H_ 18.3/2.38 (SCH_3_-5), respectively (Tables 2, 3, Figures S41–S43). These data indicated that compound **13** also contained the same 6-substituted 4-methoxy-5-methylthio-2,2′-bipyridine skeleton as SF2738 C (**9**) (Yin et al., 2016). The rest methine signal at δ_C/H_ 101.7/6.09 (CH-7), along with its HMBC correlation with the two methoxy protons at δ_H_ 3.56, suggested a methylal moiety that was linked to C-6 of the 2,2′-bipyridine nucleus from the key HMBC correlations of H-7 (δ_H_ 6.09), H-3 (δ_H_ 8.01), and methylthio proton (δ_H_ 2.38) to C-5 (δ_C_ 120.7) (Figure 6, Figure S45). Thus, compound **13**, named as pyrisulfoxin E, was elucidated as 6-dimethoxymethyl-4-methoxy-5-methylthio-2,2′-bipyridine.

**Figure 7:**
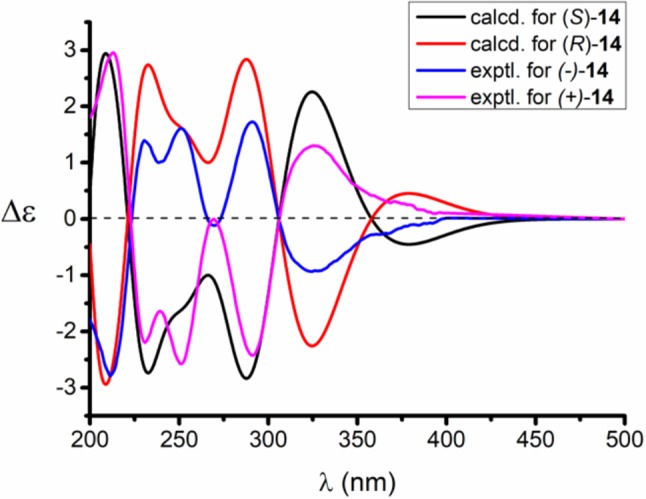
Experimental and calculated ECD spectra of *S*(+)-**14** and *R*(–)-**14** in MeOH.

Pyrisulfoxin F [(±)-**14**] was obtained as white amorphous powder. The protonated molecular ion peak at *m/z* 379.1222 [M+H]^+^ in HRESIMS indicating the molecular formula C_20_H_18_N_4_O_2_S (Figure S46). Similar to those of **13**, ^1^H NMR signals at δ_H_ 8.33 (H-3′, dd), 7.80 (H-4′, ddd), 7.30 (H-5′, m), 8.62 (H-6′, dd), 8.05 (H-3, s), 4.12 (3H, OCH_3_-4), and 2.44 (3H, SCH_3_-5) (Table 2, Figure S47) indicated the same 6-substituted 4-methoxy-5-methylthio-2,2′-bipyridine skeleton. ^1^H-^1^H COSY correlative signals of H-3′/H-4′/H-5′/H-6′ (Figure S50) and HMBC correlative signals from H-3 to C-2′ (δ_C_ 154.9), H-3 and OCH_3_-4 to C-4 (δ_C_ 167.9), as well as H-3 and SCH_3_-5 to C-5 (δ_C_ 118.6) (Figure S51) confirmed the deduction. Apart from this moiety, another four *ortho*-disubstituted benzene protons observed at δ_H_ 6.78 (H-13, dd), 7.32 (H-14, m), 6.90 (H-15, ddd), and 7.96 (H-16, dd), which was further supported by ^1^H-^1^H COSY correlations of H-13/H-14/H-15/H-16 (Figure 6, Figure S50). The rest signals were observed at δ_H_ 5.22 (NH-12) and 6.82 (NH-8), δ_C−9_ 165.2 (carbonyl) and δ_C/H_ 65.7/6.36 (CH-7) (Tables 2, 3, Figures S47–S49) along with the ^1^H-^1^H COSY correlations of H-8/H-7/H-12 (Figure S50) and the HMBC correlations of H-7 (δ_H_ 6.36) and H-14 (δ_H_ 7.32) to C-11 (δ_C_ 147.2), H-12 (δ_H_ 5.22) and H-13 (δ_H_ 6.78) to C-10 (δ_C_ 117.4), and H-16 (δ_H_ 7.96) to C-9 (δ_C_ 165.2) and C-11 (Figure 6, Figure S51). These data implied a 2-substituted 2,3-dihydroquinazolin-4(1*H*)-one moiety. Thus, the 6-substituted 4-methoxy-5-methylthio-2,2′-bipyridine and 2-substituted 2,3-dihydroquinazolin-4(1*H*)-one moieties connected together to form the constitution of racemic **14** (Figure 1), which was further separated on a chiral column into optically pure isomers (+)-**14** and (–)-**14** (Figure S52). ECD calculation of (*S*)-**14** at B3LYP/6-31G(d) level was used to determine the absolute configurations of the optically pure compounds. The results matched well with the experimental ECD spectrum of (+)-**14** that showed Cotton effects around λ_max_ (Δε) 214 (+3.14), 230 (–2.42), 251 (–2.72), 269 (+0.09), 290 (–2.51), and 325 (+1.31) nm (Figure 7). Therefore, (+)-1**4** was elucidated as *S-* configuration while (–)-1**4** as *R*- configuration.

### Biological Activity

Compounds **1**–**14** were evaluated for cytotoxic activity against a panel of human cancer cell lines by MTT, SRB, and CTG methods. As shown in Table 4, racemic (±)-**2** displayed a broad spectrum of cytotoxic activities against 26 cancer cell lines with IC_50_ values ranging from 0.92 to 9.71 μM, while compound **1** showed a selective cytotoxicity against human gastric carcinoma cell line (N87) with an IC_50_ value of 8.09 μM. As shown in Table 5, compounds **7**, **8**, and (±)-**3** showed cytotoxicity against human colorectal cancer (CRC) cell lines (HCT-116, HT-29, and P6C) and human pancreatic cancer cell line (BXPC-3) with IC_50_ values ranging from 0.048 to 3.2 μM, while compounds **7** and **8** and *S*(+)-**14** also showed cytotoxicity against human breast cancer cell line (MCF-7) with IC_50_ values of 1.6, 1.8, and 10.4 μM, respectively. The new compound *R*(–)-**14** showed inhibitory activity against HCT-116 with IC_50_ of 14.7 μM. The results indicated that 6-oxime group or 6-acetylaminomethyl and 5-methylsulfinyl substitutions might be beneficial to the cytotoxicity of these 2,2′-bipyridine derivatives. Also, 6-oxime substituted 2,2′-bipyridine derivatives [(±)-**3**, **7**, and **8**] may have high selectivity against human CRC cells, indicating their potential use in the development of anti-CRC drugs.

**Table 4:** Cytotoxicity of **1***^a^* and (±)-**2***^b^* against cancer and normal cell lines (IC_50_, μM).

**Cell lines**	**A431**	**BT474**	**MDA-MB-468**	**U251**	**HCC1954**	**MCF-7**	**MKN-45**	**Hep3B**	**H1975**
(±)-**2**	4.63	8.24	1.31	4.79	2.14	5.78	4.88	4.42	2.55
Adriamycin*^c^*	0.17	1.94	>100	0.19	0.048	0.10	0.19	17.58	0.091
**Cell lines**	**A673**	**H2228**	**MDA-MB-231**	**U87**	**Karpass299**	**HL60**	**MV-4-11**	**N87**	**H1299**
(±)-**2**	6.90	8.05	0.92	3.67	3.13	3.18	9.71	7.46	7.38
Adriamycin	0.13	0.097	0.18	0.12	0.39	0.21	0.16	0.12	0.49
**Cell lines**	**A549**	**K562**	**HCT-116**	**143B**	**B16F10**	**SPC-A1**	**HUCCT1**	**DU145**	**L02**
(±)-**2**	4.18	5.92	2.45	8.39	7.74	7.83	7.91	4.51	>100
Adriamycin	0.099	0.018	0.10	0.095	0.015	0.19	0.051	0.048	0.096

a *The IC_50_ values for **1** against N87 and other 26 cell lines were 8.09 μM and >100 μM, respectively*.

b *The IC_50_ for **2** against HEK-293F was >100 μM*.

c *Positive control with an IC_50_ value of 0.050 μM against HEK-293F cells*.

**Table 5:** Cytotoxicity of (±)-**3**, **7**, **8**, and **14** against cancer cell lines (IC_50_, μM).

**Cell lines**	**HCT-116**	**HT-29**	**P6C**	**BXPC-3**	**K562**	**Jurkat**	**MCF-7**
(±)-**3**	0.73	0.83	3.2	1.6	13.0	NT*^a^*	NT
**7**	0.048	0.095	0.60	0.28	10.0	NT	1.6
**8**	0.10	0.20	0.30	0.49	8.4	13.7	1.8
*R*(+)-**14**	NA*^c^*	NT	NT	NT	NA	NT	10.4
*S*(–)-**14**	14.7	NT	NT	NT	NA	NT	NA
Adriamycin*^b^*	0.21	0.16	0.65	0.032	0.25	0.44	0.86

a *NT, not tested*,

b *Positive control*,

c *NA, no activity*.

## Conclusions

Six new optically active caerulomycin compounds were obtained from two solid cultures of *Streptomyces albolongus* EA12432 endophytic with *Aconitum carmichaeli*. The racemic pyrisulfoxin D [(±)-**2**] showed broad-spectrum cytotoxic activities against cancer cell lines with the IC_50_ values of 0.92–9.71 μM, while *R*-Pyrisulfoxin C (**1**), *S*-pyrisulfoxin F [(+)-**14**], and *R*-pyrisulfoxin F [(–)-**14**] showed a selective cytotoxicity against N87, MCF-7, and HCT-116 cancer cell lines with the IC_50_ values of 8.09, 10.4, and 14.7 μM, respectively.

## Data Availability Statement

The raw data supporting the conclusions of this article will be made available by the authors, without undue reservation, to any qualified researcher.

## Author Contributions

All authors listed have made a substantial, direct and intellectual contribution to the work, and approved it for publication.

## Conflict of Interest

The authors declare that the research was conducted in the absence of any commercial or financial relationships that could be construed as a potential conflict of interest.
