# Supplementary material for: Cytotoxic and Optically Active Pyrisulfoxins From the Endophytic Streptomyces albolongus EA12432
Source: Front Chem. 2020 May 6;8:248. doi: 10.3389/fchem.2020.00248 (PMC7218127; doi:10.3389/fchem.2020.00248)

Supplementary Material

Cytotoxic and Optically-active Pyrisulfoxins from the Endophytic *Streptomyces albolongus* EA12432

Yuqi Du, [1,†] Chen Wang, [1,†] Guodong Cui, [1,†] Yiwen Chu, [2] Qian Jia, [1] Yi Wang, [1,*] and Weiming Zhu[1,3,*]

[1] Key Laboratory of Marine Drugs, Ministry of Education of China, School of Medicine and Pharmacy, Ocean University of China, Qingdao 266003, China

[2] Sichuan Industrial Institute of Antibiotics, Chengdu University, Chengdu 610052, China

[3] Laboratory for Marine Drugs and Bioproducts of Pilot National Laboratory for Marine Science and Technology, Qingdao 266003, China

[†] These authors contributed equally to this work.

***Correspondence:**

Weiming Zhu: weimingzhu@ouc.edu.cn

Yi Wang: wangyi0213@ouc.edu.cn

List of Supporting Information

Structures of compounds 1–14 .... S3
Table S1. ^{13}C NMR (125 M) data of compounds **5–12** S4
Table S2. ^{1}H NMR (500 M) data of compounds **5–8** S4
Table S3. ^{1}H NMR (500 M) data of compounds **9–12** S5
Physical and chemical properties of known compounds 5–12 .... S5
Table S4. Optimized Z-matrixes of (*S*)-**2** in the gas phase (Å) at B3LYP/6-31G(d) level S6
Table S5. Optimized Z-matrixes of (*R*)-**3** in the gas phase (Å) at B3LYP/6-31G(d) level S7
Table S6. Optimized Z-matrixes of (*S*)-**14** in the gas phase (Å) at B3LYP/6-31G(d) level S8
Table S7. Important thermodynamic parameters (a.u.) for (*S*)-**2** S9
Table S8. Important thermodynamic parameters (a.u.) for (*R*)-**3** S9
Table S9. Important thermodynamic parameters (a.u.) for (*S*)-**14** S10
Figure S1. HPLC profile of (±)-pyrisulfoxin D ((±)-**2**) on chiral analytic column S11
Figure S2. HPLC profile of (±)-pyrisulfoxin A ((±)-**3**) on chiral analytic column S11
Figure S3. HPLC profile of (±)-pyrisulfoxin B ((±)-**4**) on chiral analytic column S11
Figure S4. HPLC profile of (±)-4-*O*-methyl pyrisulfoxin D ((±)-**2a**) on chiral analytic column S11

Figure S5. HRESIMS spectrum of **1** ... S12
Figure S6. ^{1}H NMR spectrum of **1** in CD_3OD ... S13
Figure S7. ^{13}C NMR spectrum of **1** in CD_3OD ... S14
Figure S8. HSQC spectrum of **1** in CD_3OD ... S15
Figure S9. ^{1}H-^{1}H COSY spectrum of **1** in CD_3OD ... S17
Figure S10. HMBC spectrum of **1** in CD_3OD ... S18
Figure S11. HRESIMS spectrum of (±)-**2** ... S24
Figure S12. ^{1}H NMR spectrum of (±)-**2** in DMSO-*d*$_6$ ... S25
Figure S13. Quantitative DEPT (Q-DEPT) NMR spectrum of (±)-**2** in DMSO-*d*$_6$ ... S26
Figure S14. HSQC spectrum of (±)-**2** in DMSO-*d*$_6$ ... S27
Figure S15. ^{1}H-^{1}H COSY spectrum of (±)-**2** in DMSO-*d*$_6$ ... S30
Figure S16. HMBC spectrum of (±)-**2** in DMSO-*d*$_6$ ... S31
Figure S17. ^{1}H NMR spectrum of (±)-**2** in CD_3OD ... S36
Figure S18. ^{13}C NMR spectrum of (±)-**2** in CD_3OD ... S37
Figure S19. HSQC spectrum of (±)-**2** in CD_3OD ... S38
Figure S20. ^{1}H-^{1}H COSY spectrum of (±)-**2** in CD_3OD ... S40
Figure S21. HMBC spectrum of (±)-**2** in CD_3OD ... S41
Figure S22. ESIMS spectrum of synthetic (±)-**2** ... S44
Figure S23. ^{1}H NMR spectrum of synthetic (±)-**2** in DMSO-*d*$_6$ ... S45
Figure S24. ^{13}C NMR spectrum of synthetic (±)-**2** in DMSO-*d*$_6$ ... S46
Figure S25. HRESIMS spectrum of (±)-**2a** ... S47
Figure S26. ^{1}H NMR spectrum of (±)-**2a** in DMSO-*d*$_6$ ... S48
Figure S27. ^{13}C NMR spectrum of (±)-**2a** in DMSO-*d*$_6$ ... S49
Figure S28. HSQC spectrum of (±)-**2a** in DMSO-*d*$_6$ ... S50
Figure S29. ^{1}H-^{1}H COSY spectrum of (±)-**2a** in DMSO-*d*$_6$ ... S52
Figure S30. HMBC spectrum of (±)-**2a** in DMSO-*d*$_6$ ... S52
Figure S31. HRESIMS spectrum of (+)-**3** ... S57
Figure S32. HRESIMS spectrum of (–)-**3** ... S58
Figure S33. ^{1}H NMR spectrum of (±)-**3** in CD_3OD ... S59
Figure S34. ^{13}C NMR spectrum of (±)-**3** in CD_3OD ... S60
Figure S35. HRESIMS spectrum of (+)-**4** ... S61
Figure S36. HRESIMS spectrum of (–)-**4** ... S62
Figure S37. ^{1}H NMR spectrum of (±)-**4** in $CDCl_3$ ... S63
Figure S38. ^{13}C NMR spectrum of (±)-**4** in $CDCl_3$ ... S64
Figure S39. Co-HPLC profiles of the synthetic and the natural (±)-**2** ... S65
Figure S40. HRESIMS spectrum of **13** ... S66
Figure S41. ^{1}H NMR spectrum of **13** in $CDCl_3$ ... S67
Figure S42. ^{13}C NMR spectrum of **13** in $CDCl_3$ ... S68
Figure S43. HSQC spectrum of **13** in $CDCl_3$ ... S69
Figure S44. ^{1}H-^{1}H COSY spectrum of **13** in $CDCl_3$ ... S71
Figure S45. HMBC spectrum of **13** in $CDCl_3$ ... S72
Figure S46. HRESIMS spectrum of **14** ... S74
Figure S47. ^{1}H NMR spectrum of **14** in $CDCl_3$ ... S75

Figure S48. ^{13}C NMR spectrum of **14** in $CDCl_3$ S76
Figure S49. HSQC spectrum of **14** in $CDCl_3$ S77
Figure S50. ^{1}H-^{1}H COSY spectrum of **14** in $CDCl_3$ S79
Figure S51. HMBC spectrum of **14** in $CDCl_3$ S80
Figure S52. HPLC profile of (±)-pyrisulfoxin F ((±)-**14**) on chiral analytic column S82
Figure S53. UPLC-MS profile of EtOAc extracts from solid cultivations of strain EA12432 in 30d and 90d........S82

Structures of compounds 1–14:

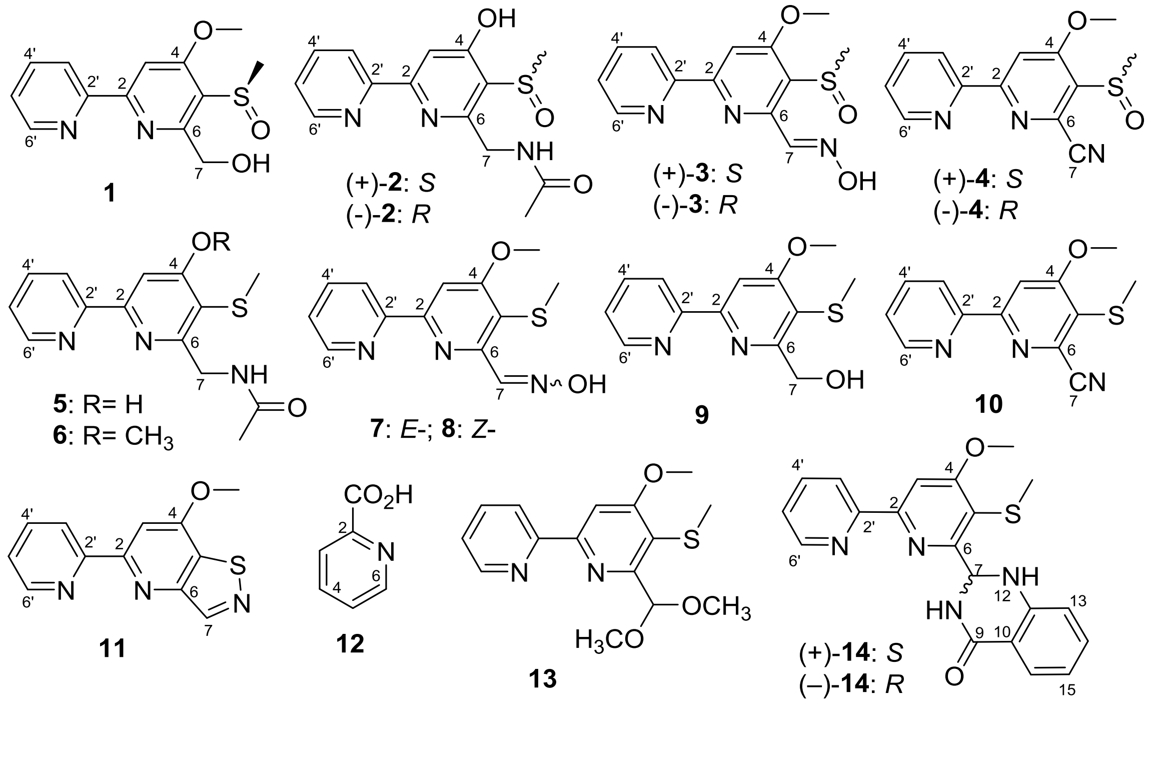

Table S1. ^{13}C NMR (125 M) data of compounds **5–12**.

no.	**5**[b]	**6**[b]	**7**[a]	**8**[a]	**9**[a]	**10**[a]	**11**[a]	**12**[a]
2	153.8, C	159.7, C	157.6, C	154.8, C	160.0, C	158.5, C	159.1, C	150.9, C
3	112.1, CH	103.7, CH	103.8, CH	105.0, CH	102.4, CH	105.8, CH	99.9, CH	127.8, CH
4	174.6, C	168.4, C	167.5, C	168.3, C	166.8, C	167.1, C	160.6, C	138.6, CH
4-OCH_3	-	56.6, CH_3	56.6, CH_3	56.8, CH_3	56.0, CH_3	56.8, CH_3	56.4, CH_3	-
5	123.2, C	122.8, C	122.2, C	121.1, C	117.3, C	127.3, C	-	123.2, CH
5-SCH_3/ 5-$SOCH_3$	17.1, CH_3	17.5, CH_3	18.7, CH_3	18.5, CH_3	17.1, CH_3	18.0, CH_3	-	-
6	147.3, C	149.9, C	152.7, C	152.6, C	154.5, C	137.3, C	154.3, C	149.8, CH
7	42.1, CH_2	44.5, CH_2	147.0, CH	140.3, C	62.1, CH_2	116.6, C	156.4, CH	-
2'	150.5, C	156.9, C	155.3, C	153.2, C	155.0, C	153.8, C	155.7, C	-
3'	121.9, CH	112.0, CH	122.0, CH	121.1, CH	120.7, CH	121.7, CH	121.7, CH	-
4'	126.3, CH	138.6, CH	137.4, CH	137.6, CH	136.6, CH	137.3, CH	137.3, CH	-
5'	124.3, CH	125.4, CH	124.5, CH	125.0, CH	123.8, CH	125.0, CH	124.5, CH	-
6'	139.0, CH	144.0, CH	149.0, CH	149.6, CH	148.7, CH	149.2, CH	149.2, CH	-
$NHCOCH_3$	22.4, CH_3	22.5, CH_3	-	-	-	-	-	-
NHCO	179.3, C	173.2, C	-	-	-	-	-	-
2-CO_2H	-	-	-	-	-	-	-	169.2, C

[a] Recorded in $CDCl_3$; [b] Recorded in CD_3OD.

Table S2. ^{1}H NMR (500 M) data of compounds **5–8**.

no.	**5**[b]	**6**[b]	**7**[a]	**8**[a]
3	7.01, s	7.91, s	8.05, s	8.11, s
4-OCH_3	-	4.07, s	4.12, s	4.14, s
5-S-CH_3/5-SO-CH_3	2.25, s	2.37, s	2.38, s	2.40, s
7	4.71, s	4.77, s	9.11, s	8.67, s
3'	7.96, dd (7.9, 0.8)	8.47, dd (8.0, 1.1)	8.53, dd (8.0, 1.2)	8.08, dd (8.0, 1.4)
4'	7.84, ddd (8.6, 7.4, 1.5)	7.94, ddd (8.3, 7.8, 1.6)	7.86, ddd (8.3, 7.8, 2.0)	7.85, ddd (8.3, 7.6, 1.7)
5'	7.38, ddd (7.7, 5.0, 0.8)	7.45, ddd (7.5, 4.9, 1.2)	7.34, ddd (7.5, 5.0, 1.0)	7.38, ddd (7.0, 5.2, 1.2)
6'	8.60, dd (4.0, 1.1)	8.64, dd (4.6, 1.3)	8.66, dd (4.4, 1.3)	8.68, dd (4.5, 1.7)
NHCO-CH_3	2.01, s	2.08, s	-	-
7-NOH	-	-	10.32, brs	-

[a] Recorded in $CDCl_3$; [b] Recorded in CD_3OD.

Table S3. ^{1}H NMR (500 M) data of compounds **9–12**.

no.	**9** [a]	**10** [a]	**11** [a]	**12** [a]
3	7.92, s	8.16, s	8.08, s	8.09, dd (8.0, 1.2)
4	-	-	-	7.92, ddd (8.1, 7.8, 1.6)
4-OCH_3	4.03, s	4.12, s	4.19, s	-
5	-	-	-	7.51, ddd (7.8, 4.8, 1.1)
5-S-CH_3/ 5-SO-CH_3	2.28, s	2.52, s	-	-
6	-	-	-	8.59, dd (4.6, 0.8)
7	4.86, s	-	9.12, s	-
3'	8.35, dd (8.0, 1.4)	8.42, dd (8.0, 1.0)	8.55, dd (8.0, 0.8)	-
4'	7.73, ddd (8.3, 7.6, 1.7)	7.83, ddd (8.5, 7.6, 1.6)	7.87, ddd (8.0, 7.6, 1.5)	-
5'	7.25, ddd (7.0, 5.2, 1.2)	7.35, ddd (7.4, 4.9, 1.0)	7.38, ddd (7.6, 5.3, 1.2)	-
6'	8.59, dd (4.5, 1.7)	8.64, dd (4.5, 1.7)	8.70, dd (4.6, 1.0)	-
7-OH	4.73, s	-	-	-

[a] Recorded in $CDCl_3$; [b] Recorded in CD_3OD.

Physical and chemical properties of known compounds 5–12

*N-(4-Hydroxy-5-methylthio-2,2'-bipyridin-6-yl)methyl acetamide (**5**):* yellow amorphous powder; UV (MeOH) λ_{max} (lg ε) 243 (4.94), 278 (4.86) nm; ^{13}C NMR and ^{1}H NMR, see Tables S1 and S2.

*SF2738 E (**6**)*: colorless needles; mp 155–158 ℃; UV (MeOH) λ_{max} (lg ε) 217 (4.24), 245 (4.02), 290 (4.06) nm; ^{13}C NMR and ^{1}H NMR, see Tables S1 and S2.

SF2738 A (7): colorless crystals, mp 180–183 ℃; UV (CH_2Cl_2) λ_{max} (lg ε) 244 (4.61) nm; ^{13}C NMR and ^{1}H NMR, see Tables S1 and S2.

*SF2738 B (**8**)*: white amorphous powder; UV (CH_2Cl_2) λ_{max} (lg ε) 245 (4.74), 295 (4.45) nm; ^{13}C NMR and ^{1}H NMR, see Tables S1 and S2.

SF2738 C (9): colorless needles; mp 108–112 ℃; UV (CH_2Cl_2) λ_{max} (lg ε) 220 (4.67), 239 (4.38), 295 (4.37) nm; ^{13}C NMR and ^{1}H NMR, see Tables S1 and S3.

*SF2738 D (**10**)*: white amorphous powder; UV (CH_2Cl_2) λ_{max} (lg ε) 240 (4.94), 292 (4.66) nm; ^{13}C NMR and ^{1}H NMR, see Tables S1 and S3.

*SF2738 F (**11**):* white amorphous powder; UV (CH_2Cl_2) λ_{max} (lg ε) 256 (4.88), 278 (4.65) nm; ^{13}C NMR and ^{1}H NMR, see Tables S1 and S3.

*Picolinic acid (**12**):* yellow crystals; mp 100–104 ℃; UV (CH_2Cl_2) λ_{max} (lg ε) 218 (4.26), 266 (3.80) nm; ^{13}C NMR and ^{1}H NMR, see Tables S1 and S3.

Table S4. Optimized Z-matrixes of (*S*)-**2** in the gas phase (Å) at B3LYP/6-31G(d) level

(*S*)-**2**-A				(*S*)-**2**-B				(*S*)-**2**-C			
C	-1.8564	-2.297	-4.6103	C	3.6052	-1.4	-2.0278	C	3.6941	-1.1335	-2.1792
N	-1.5785	-1.6756	-3.5146	N	2.4295	-0.8755	-1.9451	N	2.5028	-0.6516	-2.0669
C	-1.5226	-0.2585	-3.427	C	2.0701	0.0481	-0.9272	C	2.0346	-0.0288	-0.8789
C	-1.7691	0.4754	-4.5319	C	2.9905	0.3965	-0.0043	C	2.8643	0.0674	0.1808
C	-2.0891	-0.1984	-5.7974	C	4.3396	-0.1809	-0.0674	C	4.228	-0.4705	0.089
C	-2.1319	-1.5406	-5.8375	C	4.6392	-1.051	-1.0464	C	4.6317	-1.0525	-1.0528
C	-1.1969	0.3718	-2.1335	C	0.7029	0.6009	-0.9039	C	0.6575	0.4973	-0.834
N	-1.1433	1.6556	-2.0292	N	0.3657	1.4589	-0.0035	N	0.2106	1.0532	0.2392
C	-0.8369	2.3365	-0.8249	C	-0.9236	2.0386	0.0958	C	-1.0953	1.5878	0.3703
C	-0.5812	1.6379	0.3063	C	-1.889	1.6941	-0.7883	C	-1.9549	1.5348	-0.6739
C	-0.6316	0.1555	0.2302	C	-1.5444	0.7093	-1.8455	C	-1.4844	0.8969	-1.9301
C	-0.9261	-0.4419	-0.94	C	-0.3026	0.1909	-1.8938	C	-0.2334	0.4033	-1.9986
O	-0.3772	-0.6121	1.3199	O	-2.4638	0.3129	-2.7614	O	-2.2975	0.7936	-3.0115
C	-0.8208	3.8487	-0.9035	C	-1.1569	3.0238	1.2242	C	-1.4565	2.218	1.701
S	-0.2021	2.5007	1.793	S	-3.488	2.416	-0.6408	S	-3.565	2.2255	-0.4994
N	0.551	4.3347	-0.9611	N	0.0683	3.2812	1.9696	N	-0.3982	2.0299	2.6847
C	1.0638	5.3166	-0.0769	C	0.4097	2.5738	3.1497	C	0.6032	3.003	2.9292
C	2.5145	5.7132	-0.1602	C	1.7503	2.8015	3.7978	C	1.7351	2.6915	3.873
O	0.3514	5.8301	0.787	O	-0.3429	1.7157	3.6135	O	0.605	4.0758	2.3235
O	-0.7337	1.4392	2.8297	O	-3.9302	2.3317	-2.1513	O	-3.8309	2.597	-2.0079
C	1.5135	2.0046	1.9002	C	-4.3442	0.9311	-0.1275	C	-4.4657	0.68	-0.5412
H	-1.8851	-3.347	-4.6221	H	3.8209	-2.0785	-2.8002	H	3.992	-1.5847	-3.0799
H	-1.7362	1.5256	-4.5036	H	2.7586	1.0762	0.7631	H	2.5513	0.5226	1.0748
H	-2.2823	0.366	-6.6624	H	5.0623	0.0851	0.6475	H	4.8803	-0.4	0.9097
H	-2.3587	-2.0425	-6.732	H	5.5979	-1.4757	-1.11	H	5.6027	-1.4438	-1.1399
H	-0.9599	-1.4909	-0.9994	H	-0.0543	-0.5094	-2.6375	H	0.104	-0.0516	-2.8842
H	-0.4019	-1.5519	1.2848	H	-2.2603	-0.3125	-3.434	H	-2.0136	0.3891	-3.812
H	-1.3333	4.164	-1.8156	H	-1.5094	3.9726	0.8135	H	-1.6453	3.2831	1.5537
H	-1.3613	4.2714	-0.0558	H	-1.9238	2.6285	1.8932	H	-2.3656	1.7545	2.0908
H	1.144	3.9915	-1.6878	H	0.7472	3.8923	1.5657	H	-0.3087	1.1337	3.1165
H	2.7691	6.3851	0.6606	H	1.8069	3.8265	4.1662	H	2.2404	1.7802	3.5508
H	3.1418	4.823	-0.0991	H	1.8867	2.1135	4.6332	H	2.4534	3.5124	3.884
H	2.6994	6.2204	-1.1079	H	2.5433	2.6364	3.0674	H	1.3406	2.5486	4.8798
H	2.0721	2.4492	1.0758	H	-4.0034	0.6431	0.8678	H	-4.2388	0.105	0.3574
H	1.9297	2.3642	2.8421	H	-5.4155	1.1328	-0.0886	H	-5.5355	0.8916	-0.5637
H	1.6173	0.9211	1.859	H	-4.1644	0.1092	-0.8195	H	-4.2037	0.0908	-1.4191

Table S5. Optimized Z-matrixes of (*R*)-**3** in the gas phase (Å) at B3LYP/6-31G(d) level

(*R*)-**3**-A				(*R*)-**3**-B				(*R*)-**3**-C			
C	-4.5516	-0.9438	0.9902	C	-4.497	-1.0365	1.1016	C	1.8744	0.0979	-4.9064
N	-3.5045	-0.2849	0.6249	N	-3.4833	-0.35	0.6954	N	1.2169	0.0034	-3.8007
C	-2.8991	-0.4479	-0.6503	C	-2.9395	-0.4802	-0.6109	C	1.4818	0.8291	-2.6751
C	-3.4358	-1.3272	-1.5215	C	-3.5013	-1.357	-1.4688	C	2.4608	1.7537	-2.7582
C	-4.6284	-2.0952	-1.1396	C	-4.6574	-2.1559	-1.0407	C	3.2306	1.8897	-4.0019
C	-5.1703	-1.9109	0.076	C	-5.141	-2.0022	0.2034	C	2.9483	1.0889	-5.0432
C	-1.7029	0.3473	-0.9876	C	-1.7775	0.3444	-0.9938	C	0.6814	0.6545	-1.4481
N	-1.1398	0.2209	-2.1393	N	-1.2633	0.2395	-2.1704	N	0.9023	1.3913	-0.4151
C	0.0087	0.9451	-2.5422	C	-0.1502	0.9927	-2.6166	C	0.1837	1.2928	0.8023
C	0.5727	1.8456	-1.701	C	0.4498	1.8764	-1.7828	C	-0.8087	0.3769	0.9275
C	-0.0407	2.022	-0.3544	C	-0.1172	2.0405	-0.4148	C	-1.1025	-0.4982	-0.2439
C	-1.1292	1.2979	-0.0263	C	-1.1835	1.3008	-0.0507	C	-0.3816	-0.3547	-1.3735
O	0.5051	2.9147	0.5194	O	0.4434	2.943	0.4395	O	-2.1008	-1.4227	-0.1576
C	0.4703	0.6358	-3.9084	C	0.2646	0.7001	-4.0011	C	0.6323	2.2553	1.8296
S	1.9585	2.7962	-2.2281	S	1.8844	2.7509	-2.3115	S	-1.7164	0.2428	2.4309
O	2.6458	3.0376	-0.8308	O	2.5898	2.9471	-0.9161	O	-2.1046	-1.2829	2.364
C	1.1302	4.3798	-2.3019	C	1.1434	4.3781	-2.3712	C	-3.2756	0.8593	1.8082
C	0.0615	3.2333	1.8031	C	0.0464	3.2465	1.7419	C	-2.5333	-2.3084	-1.1449
N	1.701	0.7389	-4.2702	N	0.8124	1.5799	-4.7642	N	0.1702	2.2957	3.0295
O	2.0798	0.4286	-5.47	O	1.1348	1.3043	-5.9886	O	0.6038	3.1599	3.8923
H	-4.9608	-0.7861	1.9448	H	-4.8608	-0.9026	2.0779	H	1.6391	-0.5369	-5.7097
H	-3.0143	-1.4732	-2.4732	H	-3.1252	-1.4794	-2.4425	H	2.6839	2.3815	-1.9453
H	-5.0526	-2.7818	-1.8125	H	-5.1008	-2.8408	-1.7028	H	3.9947	2.6072	-4.0747
H	-6.0232	-2.4495	0.3691	H	-5.9669	-2.5634	0.5298	H	3.4866	1.1693	-5.9417
H	-1.577	1.4085	0.9172	H	-1.6002	1.404	0.9077	H	-0.5756	-0.9601	-2.2094
H	-0.2314	0.2633	-4.5998	H	0.0493	-0.2517	-4.397	H	1.3825	2.9454	1.5624
H	0.3926	4.3642	-3.1053	H	0.4061	4.4099	-3.1744	H	-3.1724	1.9165	1.5603
H	0.6313	4.6136	-1.3625	H	0.6577	4.6305	-1.4297	H	-3.5957	0.3134	0.9219
H	1.8659	5.1568	-2.5139	H	1.9203	5.1157	-2.5767	H	-4.0359	0.7517	2.583
H	0.0931	2.3505	2.4427	H	0.7081	4.0165	2.1405	H	-3.3563	-2.9035	-0.747
H	0.7181	3.9968	2.2222	H	-0.9769	3.6239	1.7459	H	-2.8861	-1.7573	-2.0175
H	-0.9556	3.6252	1.7642	H	0.1157	2.36	2.3734	H	-1.7214	-2.9771	-1.4335
H	2.9839	0.5129	-5.7142	H	1.5353	1.9616	-6.5287	H	0.2514	3.1716	4.7639

Table S6. Optimized Z-matrixes of (*S*)-**14** in the gas phase (Å) at B3LYP/6-31G(d) level

(*S*)-**14**-A				(*S*)-**14**-B				(*S*)-**14**-C				(*S*)-**14**-D			
C	-1.7827	-3.1454	2.2959	C	-3.8916	2.4527	-0.669	C	-1.8571	2.0431	-4.4725	C	0.5485	-4.9012	-0.1687
C	-3.2202	-3.31	2.0486	C	-4.3393	2.9728	-1.9665	C	-0.8254	2.7704	-5.2217	C	-0.4165	-5.723	-0.9091
C	-3.7539	-2.7799	0.9353	C	-3.6275	2.6766	-3.0669	C	0.4658	2.5824	-4.9012	C	-1.2447	-5.1259	-1.7825
C	-2.8886	-2.0522	-0.0019	C	-2.4243	1.8433	-2.9441	C	0.8107	1.6581	-3.8131	C	-1.1633	-3.6721	-1.9737
C	-1.5719	-1.9247	0.2644	C	-2.0483	1.3864	-1.7314	C	-0.1699	1.0104	-3.1499	C	-0.2606	-2.9547	-1.2726
N	-1.0492	-2.5007	1.453	N	-2.8302	1.7234	-0.5941	N	-1.5218	1.238	-3.522	N	0.5953	-3.6262	-0.3592
C	-0.666	-1.2036	-0.6489	C	-0.849	0.5454	-1.5606	C	0.1295	0.0739	-2.0508	C	-0.1437	-1.4933	-1.4325
N	-1.1294	-0.6798	-1.7301	N	-0.1128	0.2628	-2.5792	N	1.3495	-0.1131	-1.6823	N	-0.8895	-0.8801	-2.2851
C	-0.3468	0.03	-2.6747	C	1.0523	-0.5397	-2.5146	C	1.7364	-0.992	-0.6413	C	-0.8525	0.5178	-2.5086
C	0.9746	0.1973	-2.4448	C	1.4363	-1.0607	-1.3285	C	0.7913	-1.692	0.0238	C	0.0012	1.2821	-1.7928
C	1.562	-0.3962	-1.2204	C	0.63	-0.7617	-0.1235	C	-0.6283	-1.5127	-0.357	C	0.8873	0.6285	-0.8034
C	0.7685	-1.0665	-0.3616	C	-0.4677	0.0116	-0.2442	C	-0.9375	-0.6617	-1.3559	C	0.811	-0.7071	-0.6365
O	2.8994	-0.2347	-1.0097	O	1.0277	-1.2823	1.07	O	-1.5762	-2.2181	0.3193	O	1.7528	1.4042	-0.0944
C	3.6464	-0.7109	0.0681	C	0.4085	-1.1251	2.3101	C	-2.9559	-2.1934	0.1145	C	2.6641	0.9858	0.8754
S	2.1162	1.098	-3.5349	S	2.9016	-2.1279	-1.1947	S	1.2299	-2.8428	1.3606	S	0.0592	3.0856	-2.0123
C	1.6369	2.3636	-4.7918	C	3.4618	-2.8675	0.404	C	-0.0264	-3.838	2.2816	C	1.1929	4.1772	-1.0428
C	-1.1008	0.5233	-3.8926	C	1.7715	-0.7738	-3.8295	C	3.2268	-1.0752	-0.3717	C	-1.8196	1.0613	-3.5438
N	-1.9803	-0.5395	-4.3725	N	1.0094	-1.7458	-4.6051	N	3.8856	-1.5984	-1.5636	N	-1.9456	0.1067	-4.6413
N	-1.8358	1.7292	-3.5354	N	1.9298	0.4975	-4.524	N	3.7137	0.2461	0.0013	N	-3.0971	1.3302	-2.8983
C	-3.2188	1.69	-3.2485	C	1.0821	0.865	-5.5925	C	4.3851	1.0727	-0.9271	C	-4.1741	0.421	-2.9964
C	-3.3449	-0.6186	-3.9938	C	0.0987	-1.3572	-5.6206	C	4.4802	-0.7498	-2.5321	C	-2.9818	-0.8612	-4.681
C	-3.9683	0.4415	-3.4409	C	0.1063	-0.1016	-6.1128	C	4.7273	0.5469	-2.2555	C	-4.0678	-0.7397	-3.8908
C	-5.3803	0.3289	-3.0306	C	-0.8591	0.2815	-7.1599	C	5.3291	1.4141	-3.2854	C	-5.1219	-1.7702	-3.9313
C	-6.0442	-0.828	-3.2009	C	-1.7451	-0.6167	-7.6248	C	5.6312	0.916	-4.4972	C	-5	-2.8311	-4.7484
C	-5.3658	-1.9819	-3.8035	C	-1.7511	-1.9847	-7.0924	C	5.3666	-0.4964	-4.7971	C	-3.818	-2.9631	-5.6089
C	-4.0805	-1.8813	-4.1849	C	-0.8719	-2.3382	-6.1387	C	4.8199	-1.2908	-3.8603	C	-2.8577	-2.0227	-5.5797
O	-3.7741	2.6992	-2.813	O	1.1532	2.0036	-6.0563	O	4.6531	2.2344	-0.6189	O	-5.1862	0.6073	-2.3201
H	-1.348	-3.5531	3.161	H	-4.4355	2.6737	0.2021	H	-2.8717	2.1771	-4.7094	H	1.2036	-5.3506	0.5186
H	-3.8209	-3.8343	2.7326	H	-5.203	3.5671	-2.0318	H	-1.0943	3.4272	-5.9962	H	-0.4577	-6.7616	-0.7569
H	-4.7801	-2.8839	0.7351	H	-3.9272	3.0367	-4.0074	H	1.2231	3.091	-5.4225	H	-1.9445	-5.6919	-2.3247
H	-3.3091	-1.6386	-0.8718	H	-1.8703	1.612	-3.8071	H	1.8217	1.5128	-3.5649	H	-1.8135	-3.2079	-2.6566
H	1.166	-1.4926	0.512	H	-1.0562	0.2369	0.5959	H	-1.9372	-0.5186	-1.6443	H	1.4287	-1.1938	0.0595
H	3.275	-0.2853	1.0012	H	-0.6081	-1.5186	2.2765	H	-3.3433	-1.1873	0.2797	H	2.1395	0.5112	1.7056
H	3.5926	-1.7994	0.1123	H	0.9766	-1.6777	3.0595	H	-3.1922	-2.5179	-0.8996	H	3.3798	0.2857	0.4427
H	4.6873	-0.4138	-0.0662	H	0.3871	-0.071	2.5893	H	-3.4288	-2.8748	0.8228	H	3.2037	1.8566	1.25
H	0.7727	2.9264	-4.4429	H	2.6761	-3.5066	0.8066	H	-0.5741	-4.4717	1.584	H	0.9834	4.0692	0.0214
H	2.4706	3.0499	-4.9427	H	4.3585	-3.4629	0.2315	H	0.4783	-4.4639	3.0176	H	2.2277	3.8986	-1.2418
H	1.4015	1.8737	-5.7364	H	3.6875	-2.0732	1.1155	H	-0.7205	-3.1699	2.7911	H	1.0351	5.2145	-1.3382
H	-0.4191	0.7554	-4.7082	H	2.7718	-1.1778	-3.6686	H	3.4495	-1.7521	0.4535	H	-1.4469	1.9935	-3.9722
H	-1.5611	-1.3297	-4.817	H	1.0027	-2.6945	-4.2929	H	3.7518	-2.5623	-1.7887	H	-1.1743	0.008	-5.2683
H	-1.3279	2.5657	-3.3408	H	2.5619	1.1745	-4.1515	H	3.4492	0.6253	0.8861	H	-3.1659	2.0967	-2.2625
H	-5.8735	1.1503	-2.598	H	-0.8616	1.2592	-7.5463	H	5.5212	2.4268	-3.0791	H	-5.9715	-1.6833	-3.3183
H	-7.0495	-0.9074	-2.9048	H	-2.4333	-0.3415	-8.3699	H	6.0579	1.536	-5.2308	H	-5.7509	-3.566	-4.7736
H	-5.8866	-2.8849	-3.9372	H	-2.4429	-2.6862	-7.4582	H	5.6061	-0.885	-5.7438	H	-3.7249	-3.7924	-6.2477
H	-3.5985	-2.7108	-4.6149	H	-0.8809	-3.319	-5.7602	H	4.6317	-2.3015	-4.08	H	-2.0123	-2.1223	-6.1966

Table S7. Important thermodynamic parameters (a.u.) for (*S*)-**2**

Conformer	Total energy	Sum of electronic and zero-point Energies	Sum of electronic and thermal Energies	Sum of electronic and thermal Enthalpies	Sum of electronic and thermal Free Energies
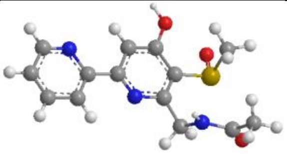 (*S*)-**2**-A (81.0%)	-1330.60442620	-1330.325366	-1330.305133	-1330.304189	-1330.376014
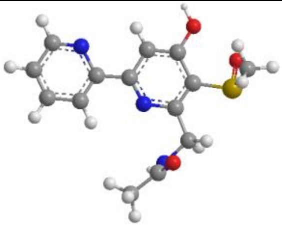 (*S*)-**2**-B (9.5%)	-1330.60240968	-1330.324350	-1330.303687	-1330.302743	-1330.376913
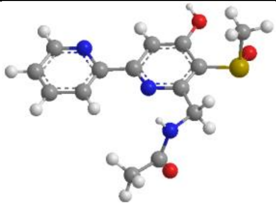 (*S*)-**2**-C (9.5%)	-1330.60240968	-1330.324350	-1330.303687	-1330.302743	-1330.376912

Table S8. Important thermodynamic parameters (a.u.) for (*R*)-**3**

Conformer	Total energy	Sum of electronic and zero-point Energies	Sum of electronic and thermal Energies	Sum of electronic and thermal Enthalpies	Sum of electronic and thermal Free Energies
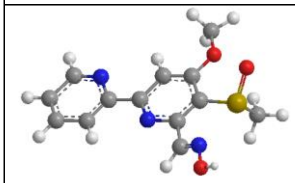 (*R*)-**3**-A (33.4%)	-1291.20095277	-1290.951800	-1290.932850	-1290.931906	-1290.999625
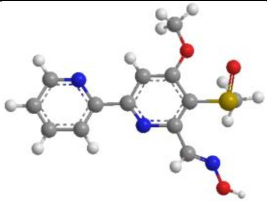 (*R*)-**3**-B (33.3%)	-1291.20095274	-1290.951801	-1290.932850	-1290.931906	-1290.999628
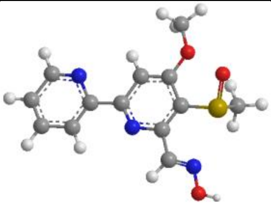 (*R*)-**3**-C (33.3%)	-1291.20095272	-1290.951801	-1290.932849	-1290.931905	-1290.999627

Table S9. Important thermodynamic parameters (a.u.) for (*S*)-**14**

Conformer	Total energy	Sum of electronic and zero-point Energies	Sum of electronic and thermal Energies	Sum of electronic and thermal Enthalpies	Sum of electronic and thermal Free Energies
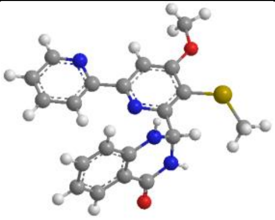 (*S*)-**14**-A (33.4%)	-1540.62905573	-1540.275984	-1540.252728	-1540.251784	-1540.330259
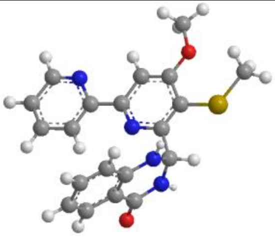 (*S*)-**14**-B (33.4%)	-1540.62905550	-1540.275984	-1540.252729	-1540.251785	-1540.330255
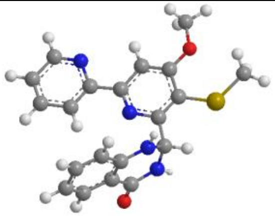 (*S*)-**14**-C (23.4%)	-1540.62872090	-1540.275457	-1540.252323	-1540.251378	-1540.329394
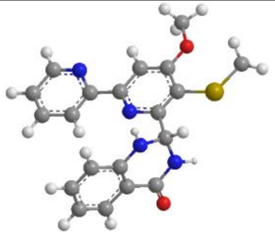 (*S*)-**14**-D (9.8%)	-1540.62789666	-1540.274497	-1540.251398	-1540.250454	-1540.328499

Fig. S1. HPLC profile of (±)-pyrisulfoxin D ((±)-**2**) on chiral analytic column.

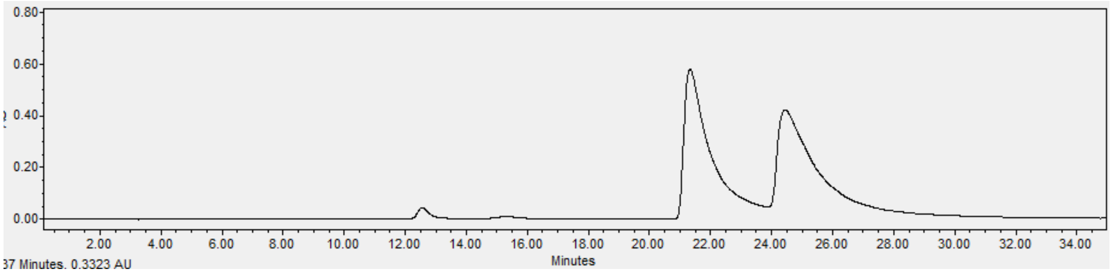

(+)-**2**: t_R = 21.0 min; (–)-**2**: t_R = 24.0 min (Chiral INB 5u analytical column; 20% EtOH/*n*-C_6H_{14} with 0.5 ‰ Et_2NH)

Fig. S2. HPLC profile of (±)-pyrisulfoxin A ((±)-**3**) on chiral analytic column.

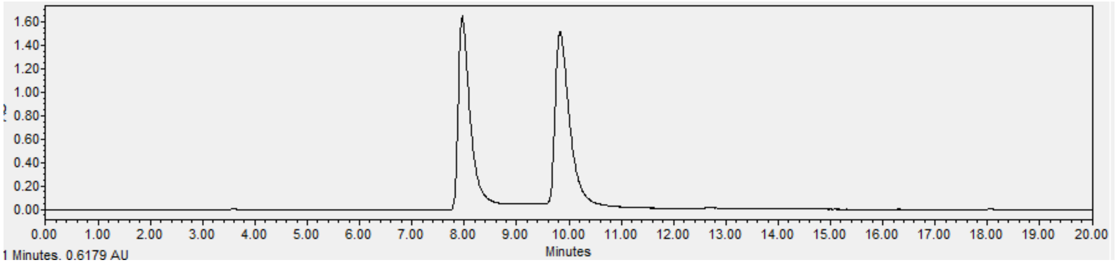

(+)-**3**: t_R = 8.0 min; (–)-**3**: t_R = 9.5 min (ChiralPak IA analytical column; 35% EtOH/*n*-C_6H_{14}

Fig. S3. HPLC profile of (±)-pyrisulfoxin B ((±)-**4**) on chiral analytic column.

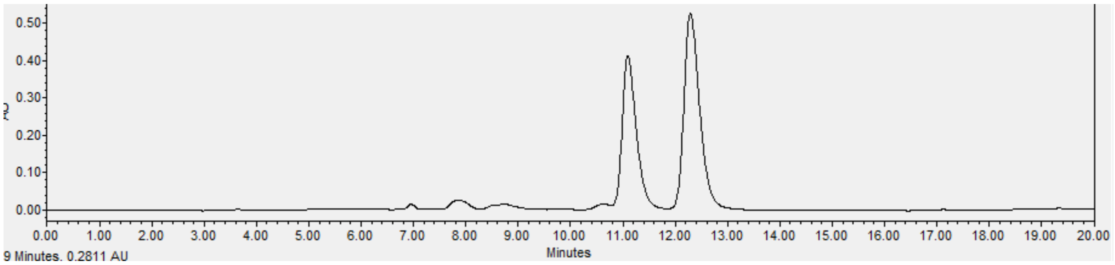

(+)-**4**: t_R = 10.8 min; (–)-**4**: t_R = 12.0 min (ChiralPak IA analytical column; 40% EtOH/*n*-C_6H_{14})

Fig. S4. HPLC profile of (±)-4-*O*-methylpyrisulfoxin D ((±)-**2a**) on chiral analytic column.

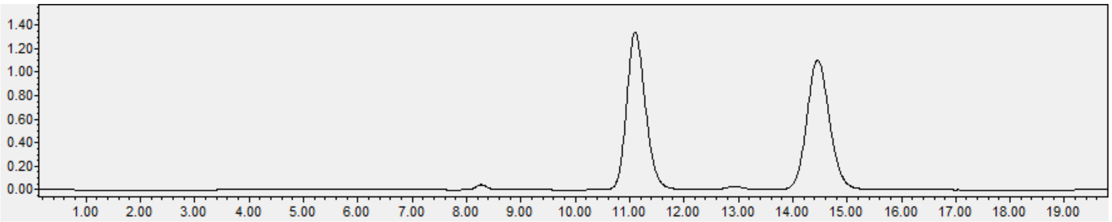

(+)-**2a**: t_R = 10.8 min; (–)-**2a**: t_R = 14.0 min (Chiral INC 5u analytical column; 35% EtOH/*n*-C_6H_{14})

Fig. S5. HRESIMS spectrum of **1**.

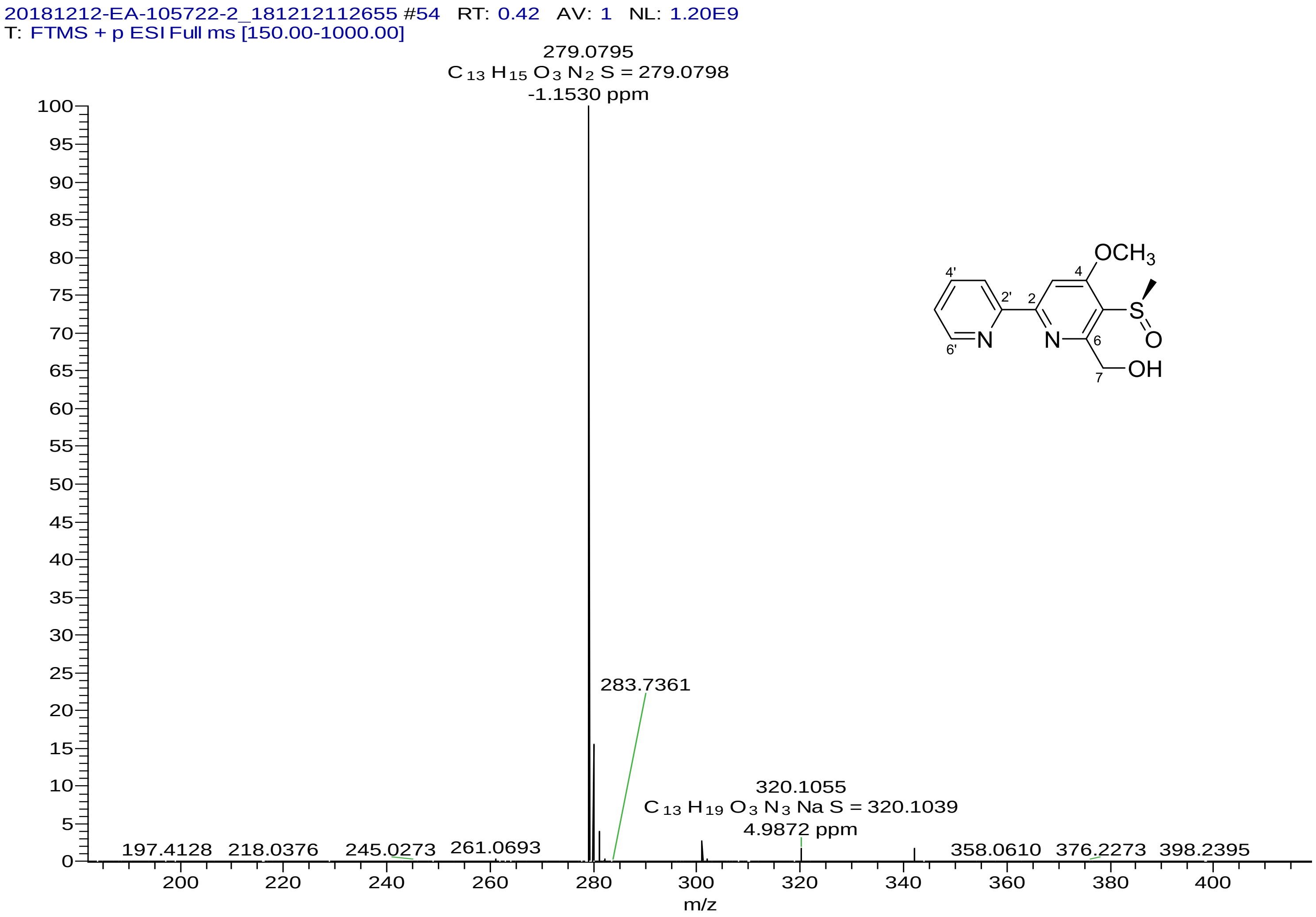

Fig. S6. ^{1}H NMR spectrum of **1** in CD_3OD.

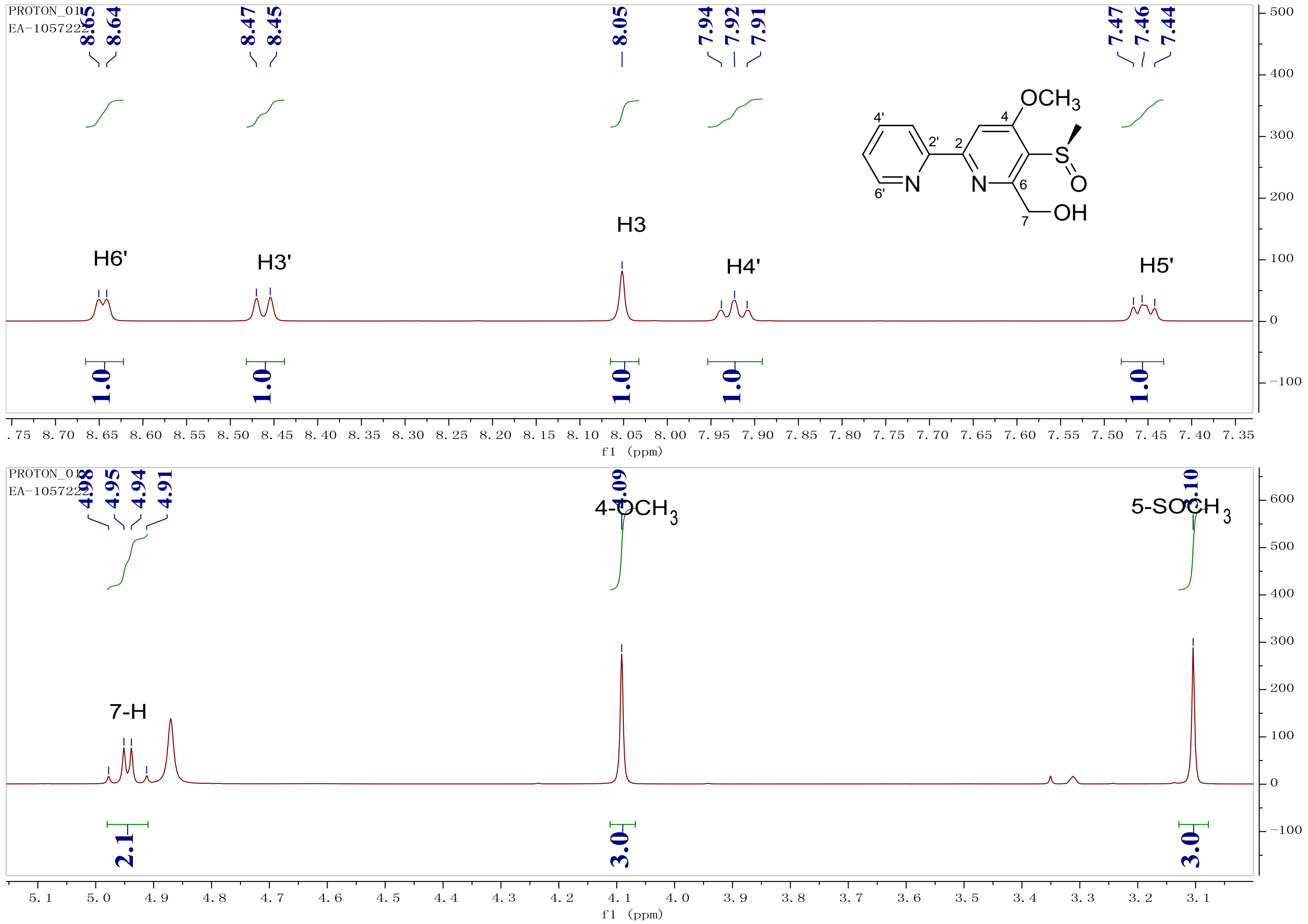

Fig. S7. ^{13}C NMR spectrum of **1** in CD_3OD.

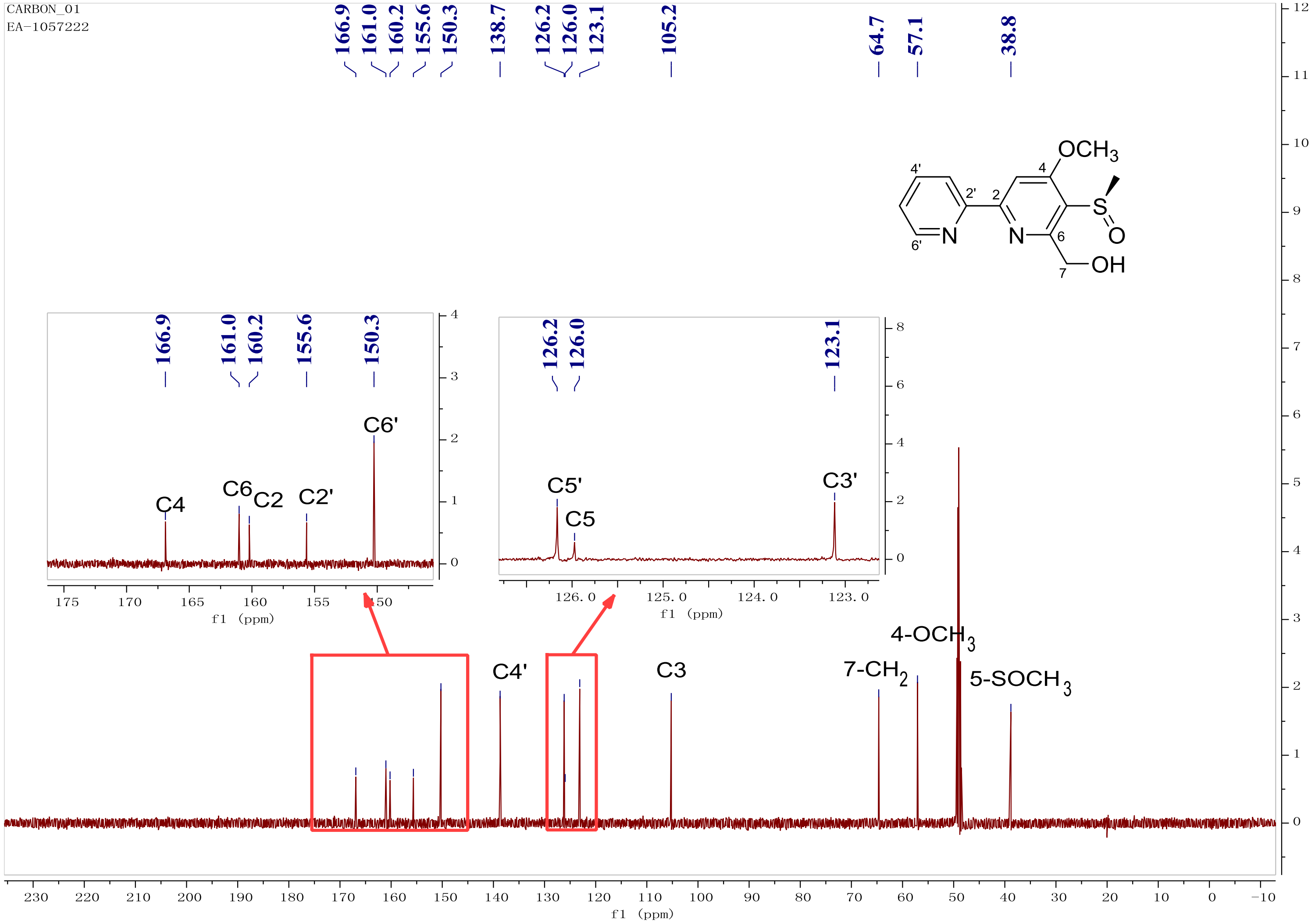

Fig. S8. HSQC spectrum of **1** in CD_3OD.

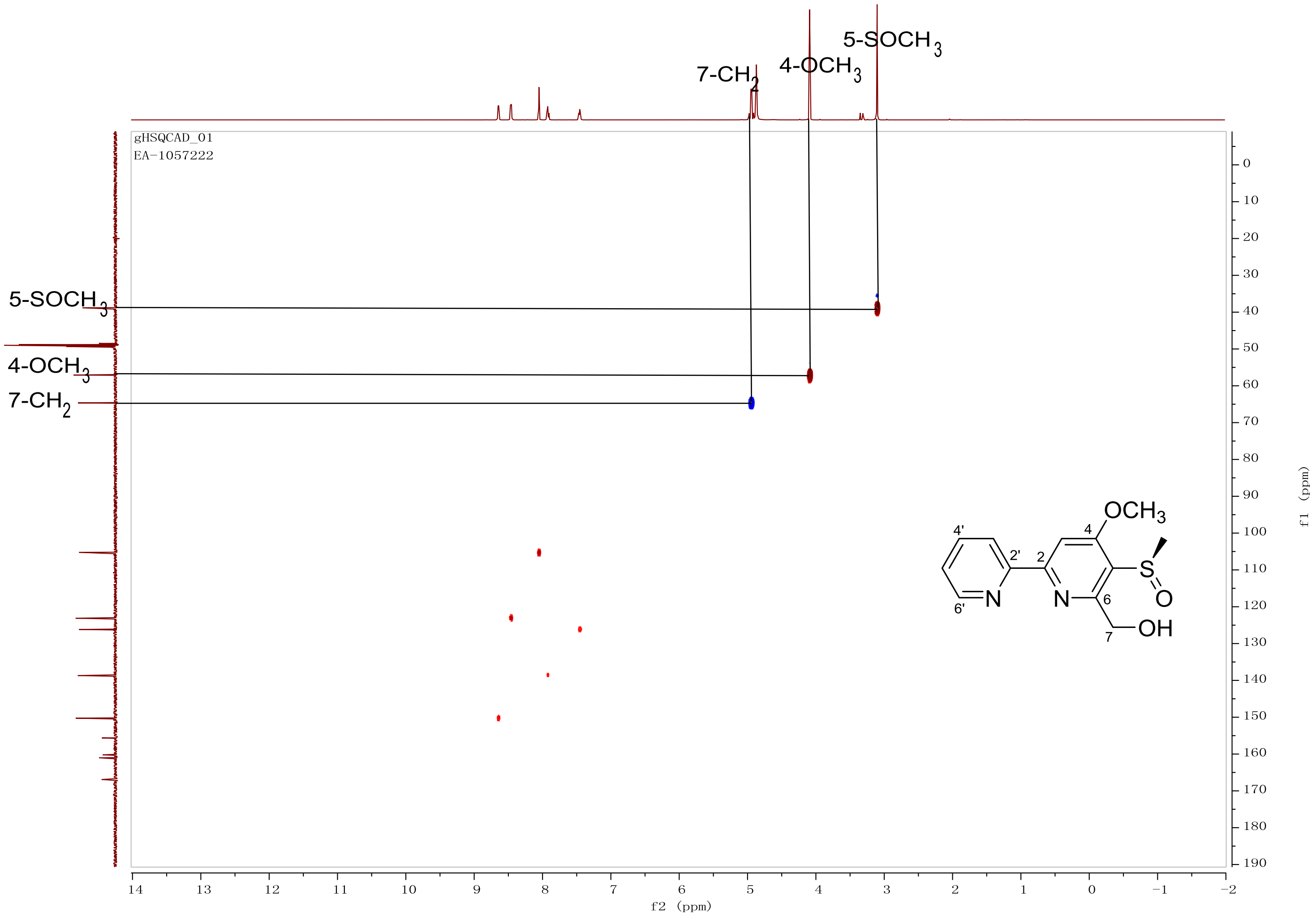

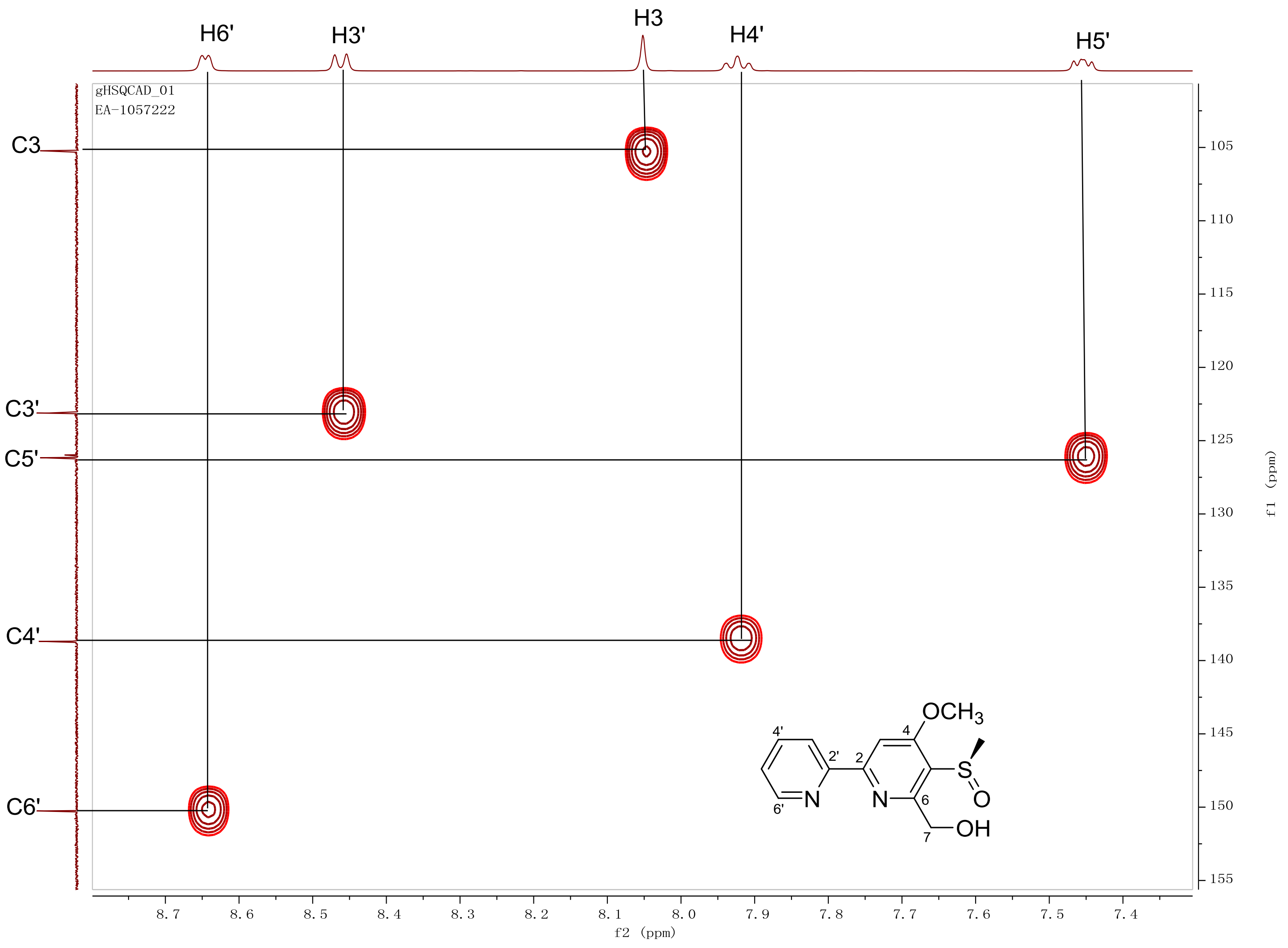
H6'
H3'
H3
H4'
H5'
gHSQCAD_01
EA-1057222
C3
C3'
C5'
C4'
C6'
OCH3
4'
2'
2
4
6
6'
7
N
N
S
O
OH
f1 (ppm)
f2 (ppm)

Fig. S9. ^1H–^1H COSY spectrum of **1** in CD_3OD.

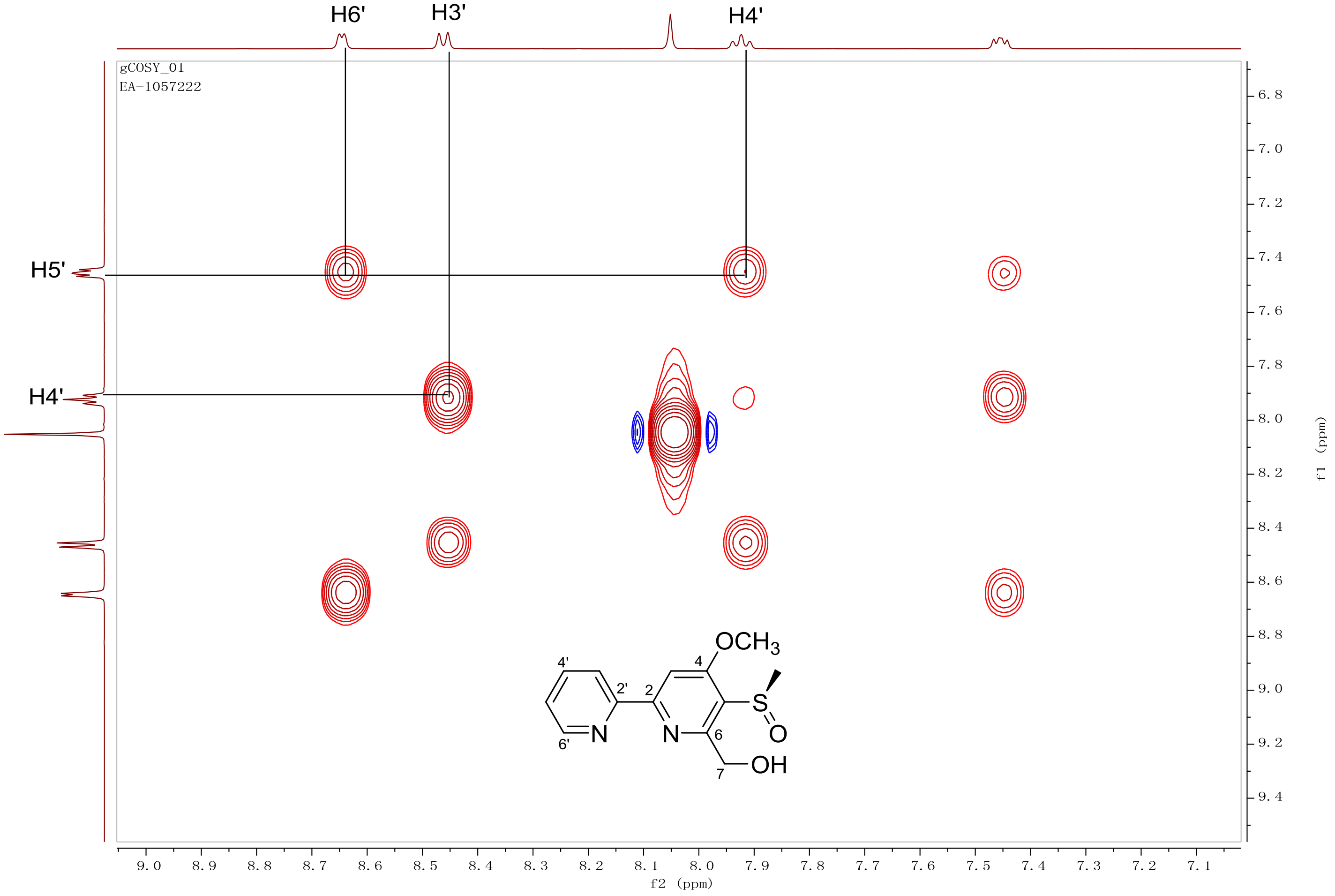

Fig. S10. HMBC spectrum of **1** in CD_3OD.

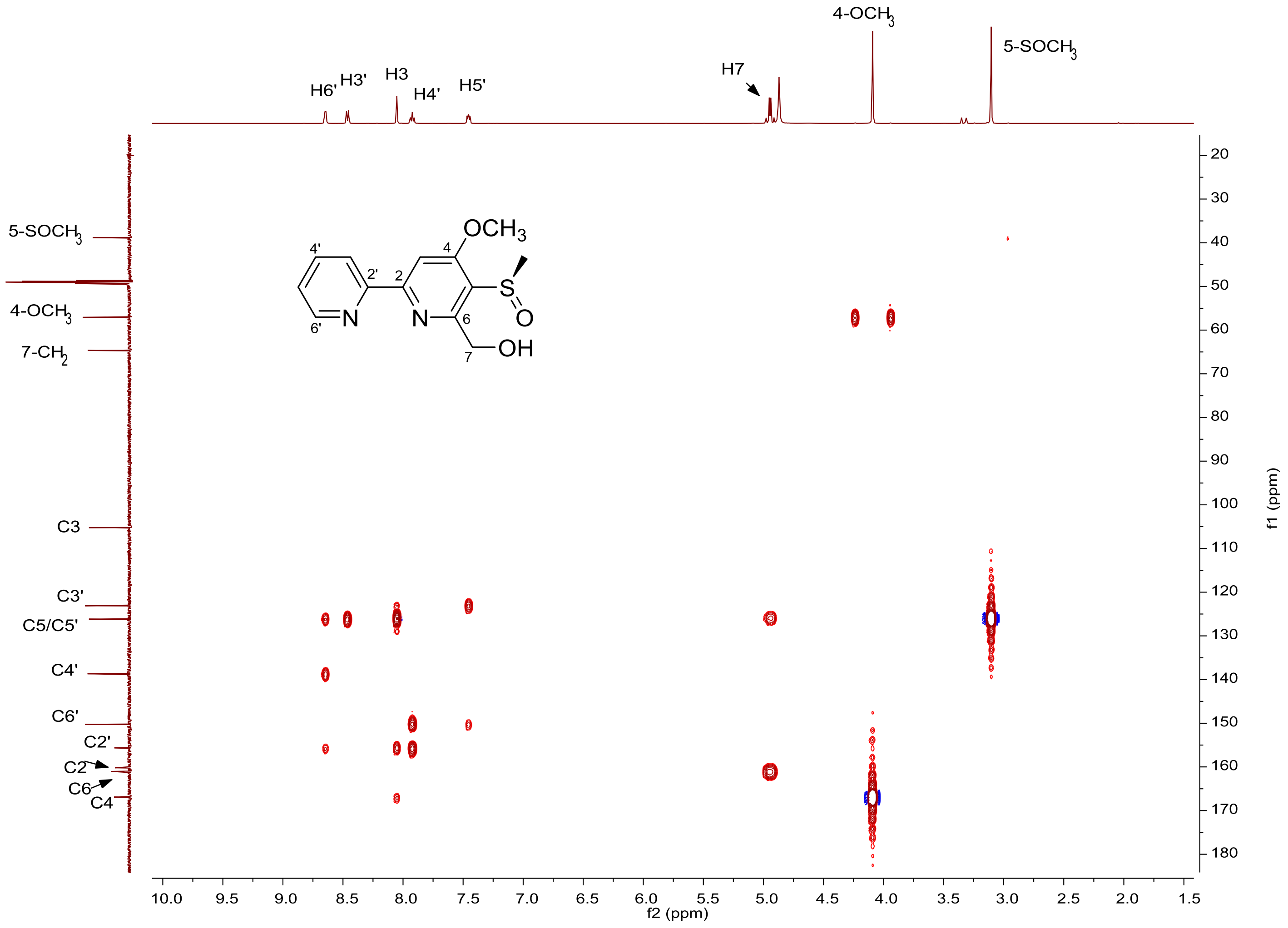

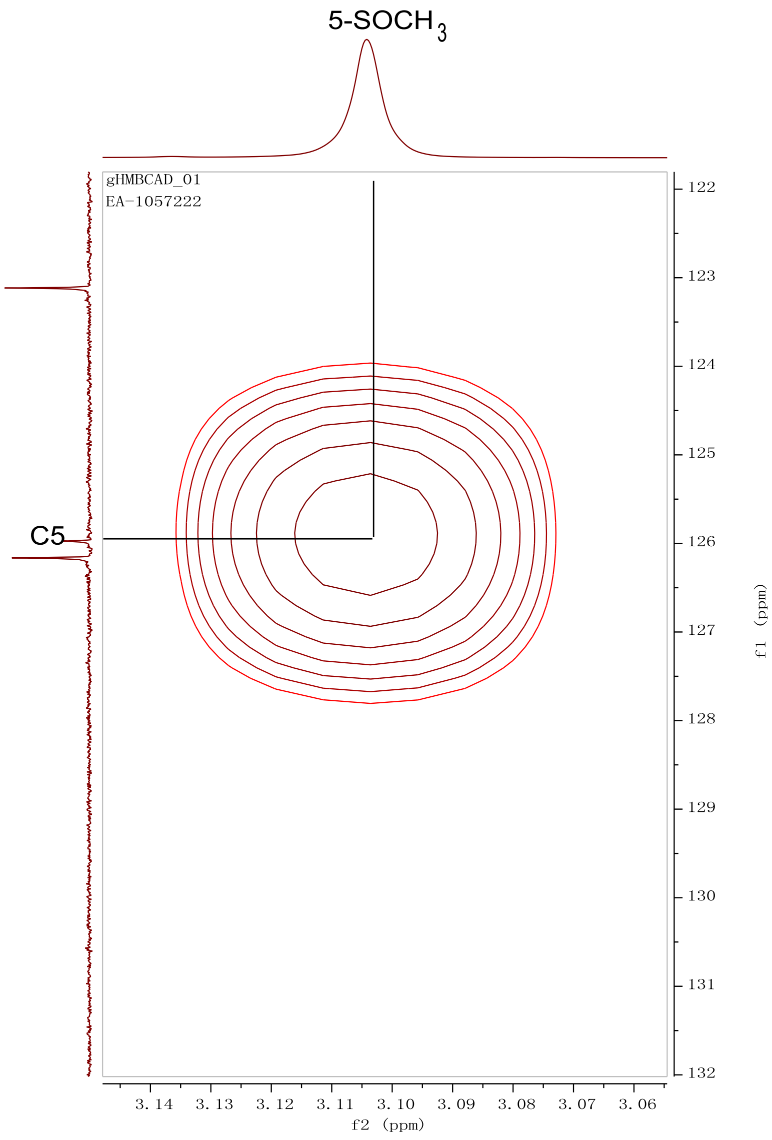
5-SOCH3
gHMBCAD_01
EA-1057222
C5
f1 (ppm)
f2 (ppm)

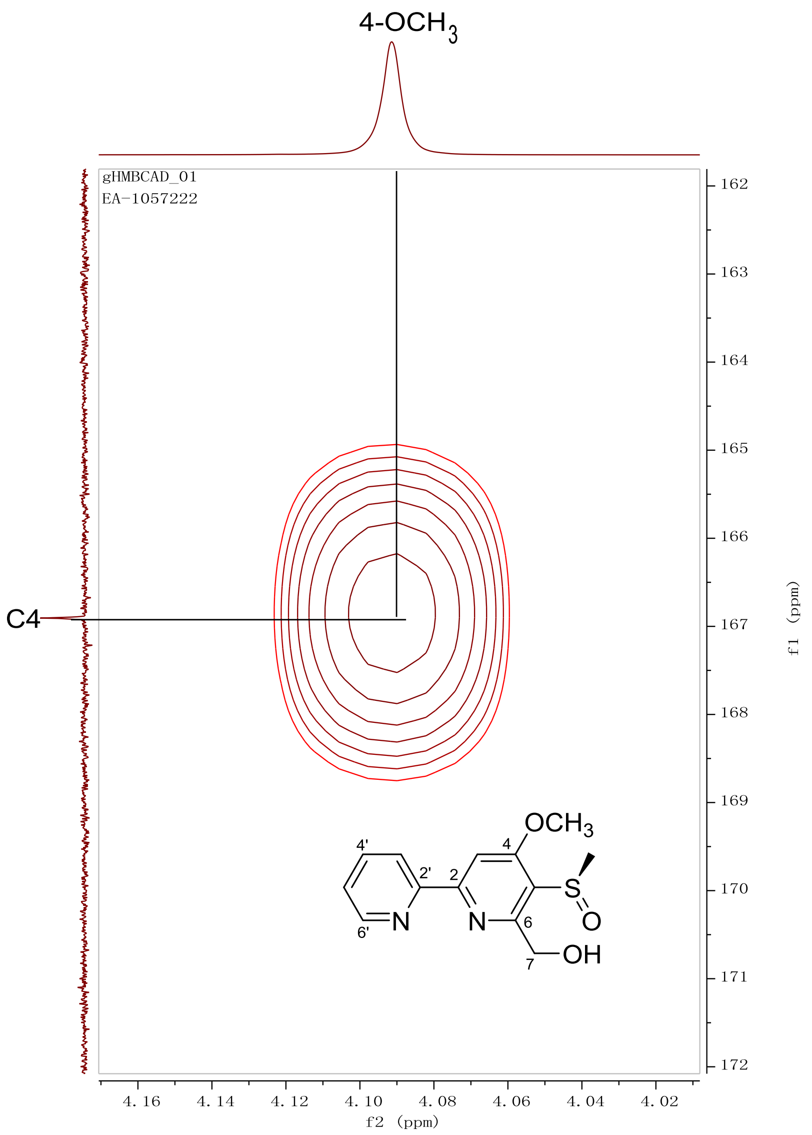
4-OCH3
gHMBCAD_01
EA-1057222
C4
OCH3
OH
f1 (ppm)
f2 (ppm)

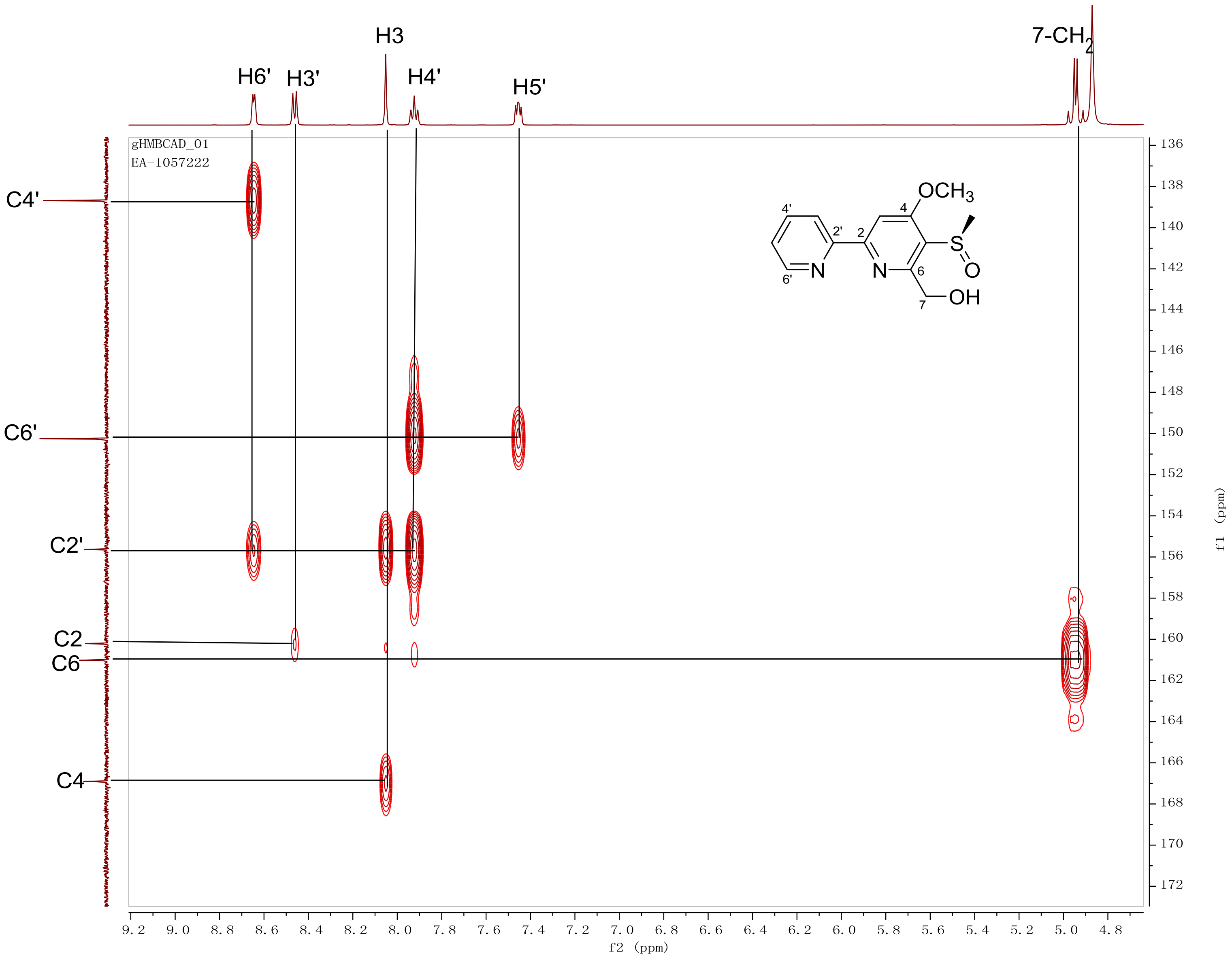
H6'
H3'
H3
H4'
H5'
7-CH2
gHMBCAD_01
EA-1057222
C4'
C6'
C2'
C2
C6
C4
OCH3
S
O
N
N
OH
4'
2'
2
4
6
6'
7
f2 (ppm)
f1 (ppm)

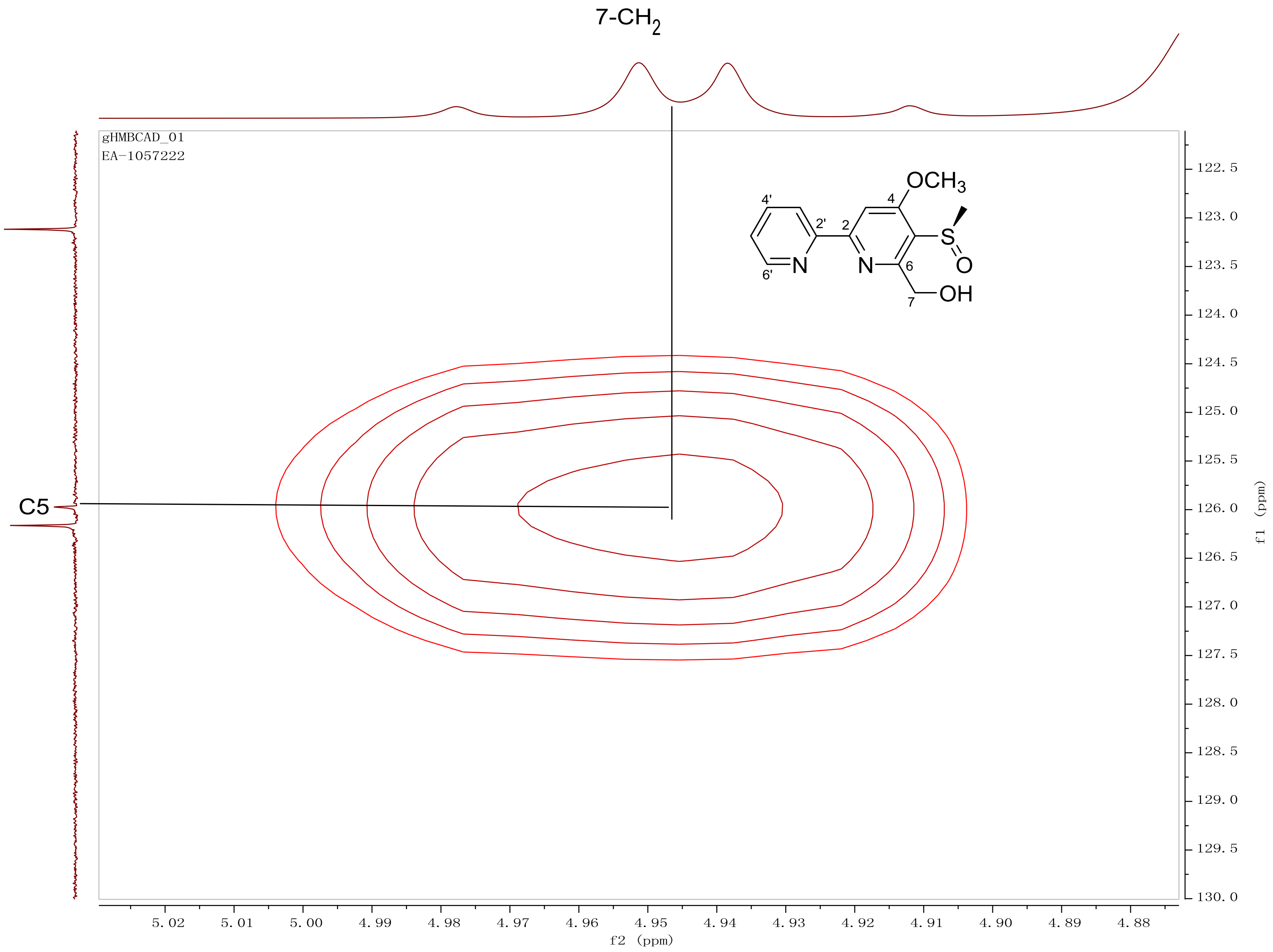
7-CH2
gHMBCAD_01
EA-1057222
OCH3
4'
2'
2
4
S
O
N
N
6
6'
7
OH
C5
122.5
123.0
123.5
124.0
124.5
125.0
125.5
126.0
126.5
127.0
127.5
128.0
128.5
129.0
129.5
130.0
f1 (ppm)
5.02
5.01
5.00
4.99
4.98
4.97
4.96
4.95
4.94
4.93
4.92
4.91
4.90
4.89
4.88
f2 (ppm)

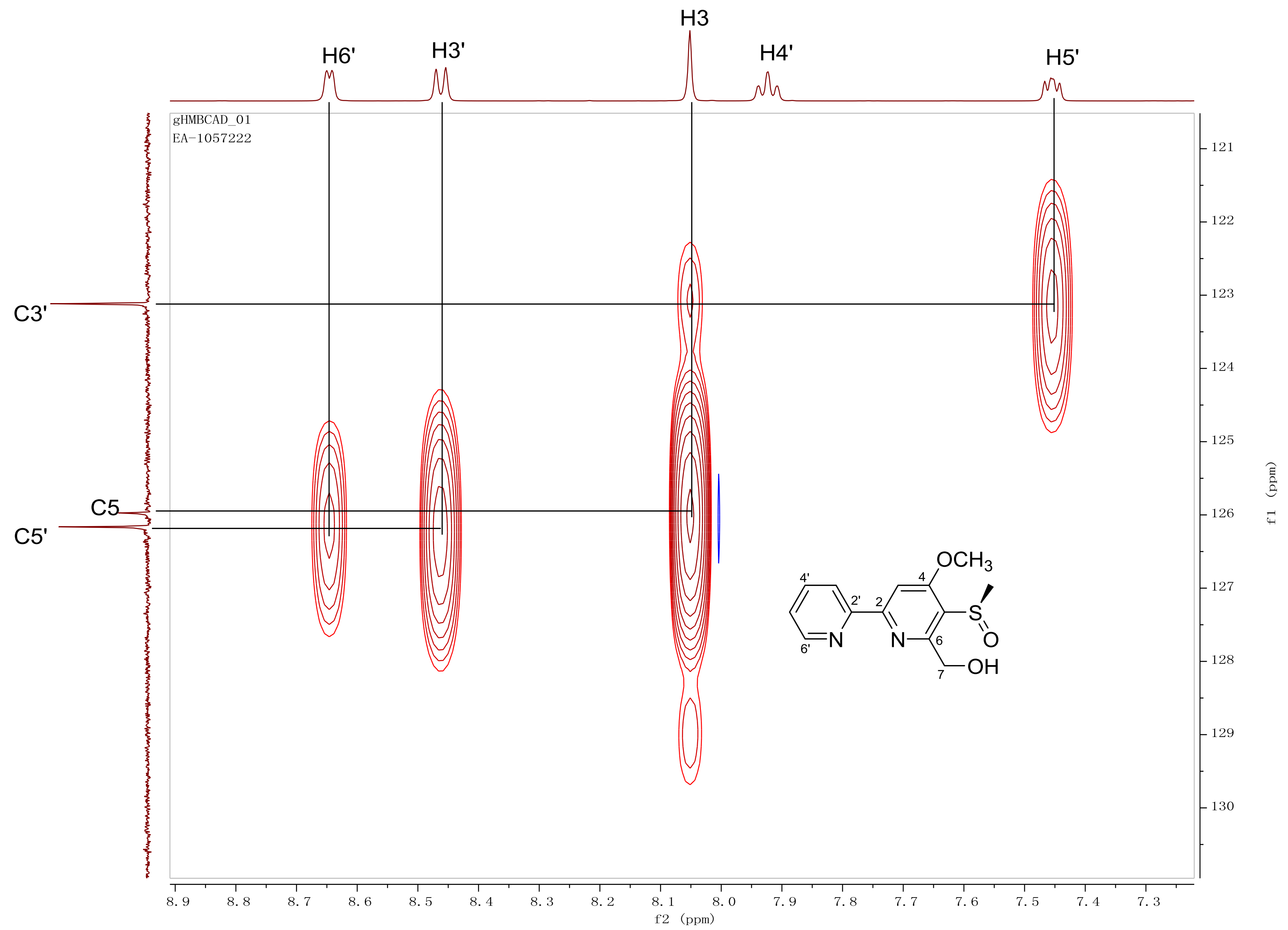

H6'
H3'
H3
H4'
H5'
gHMBCAD_01
EA-1057222
C3'
C5
C5'
121
122
123
124
125
126
127
128
129
130
f1 (ppm)
8.9
8.8
8.7
8.6
8.5
8.4
8.3
8.2
8.1
8.0
7.9
7.8
7.7
7.6
7.5
7.4
7.3
f2 (ppm)
OCH3
4'
4
2'
2
S
O
N
N
6
6'
7
OH

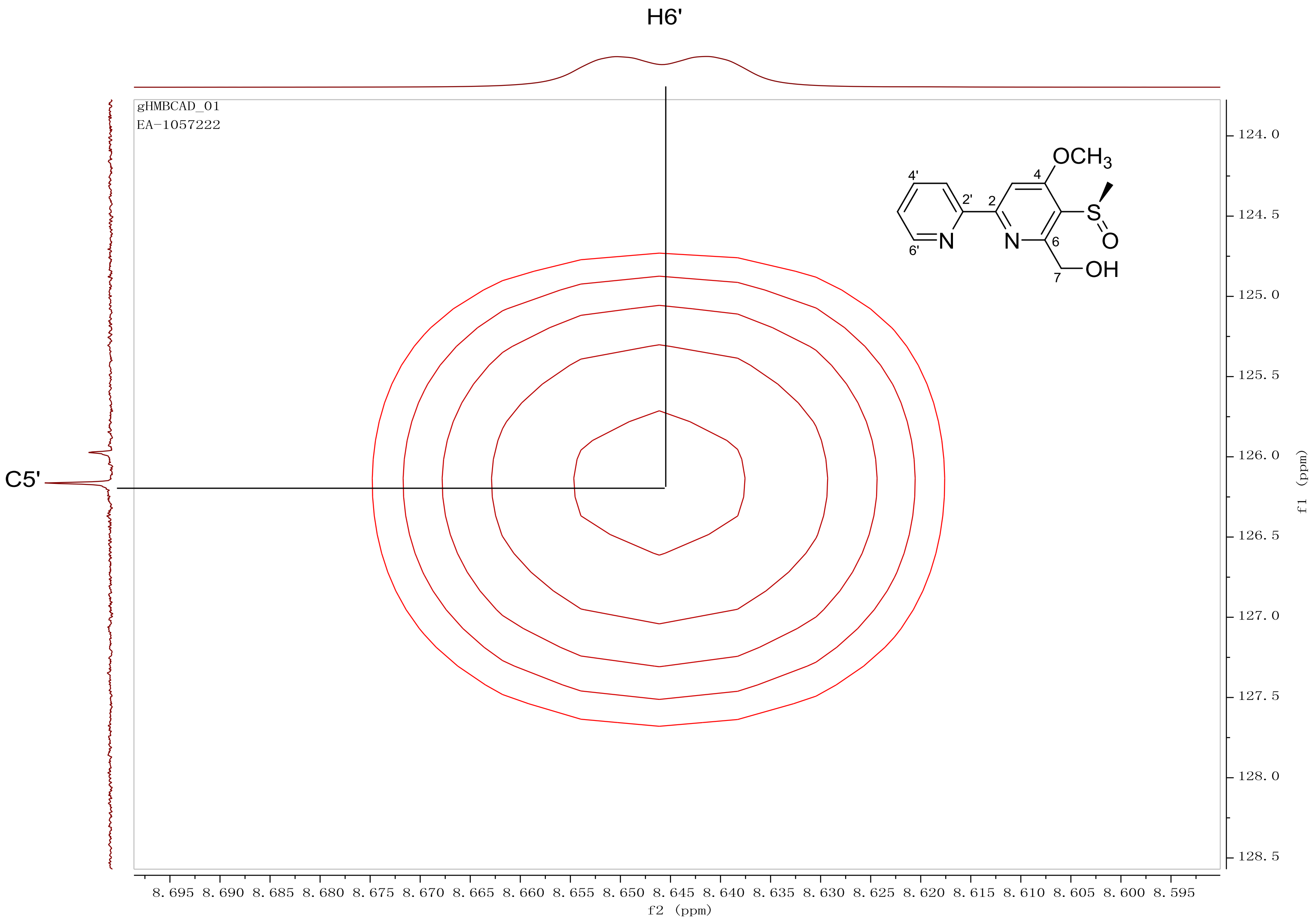
H6'
C5'
gHMBCAD_01
EA-1057222
OCH3
4'
2'
2
4
S
O
6'
N
N
6
7
OH
124.0
124.5
125.0
125.5
126.0
126.5
127.0
127.5
128.0
128.5
f1 (ppm)
8.695 8.690 8.685 8.680 8.675 8.670 8.665 8.660 8.655 8.650 8.645 8.640 8.635 8.630 8.625 8.620 8.615 8.610 8.605 8.600 8.595
f2 (ppm)

Fig. S11. HRESIMS spectrum of (±)-**2**.

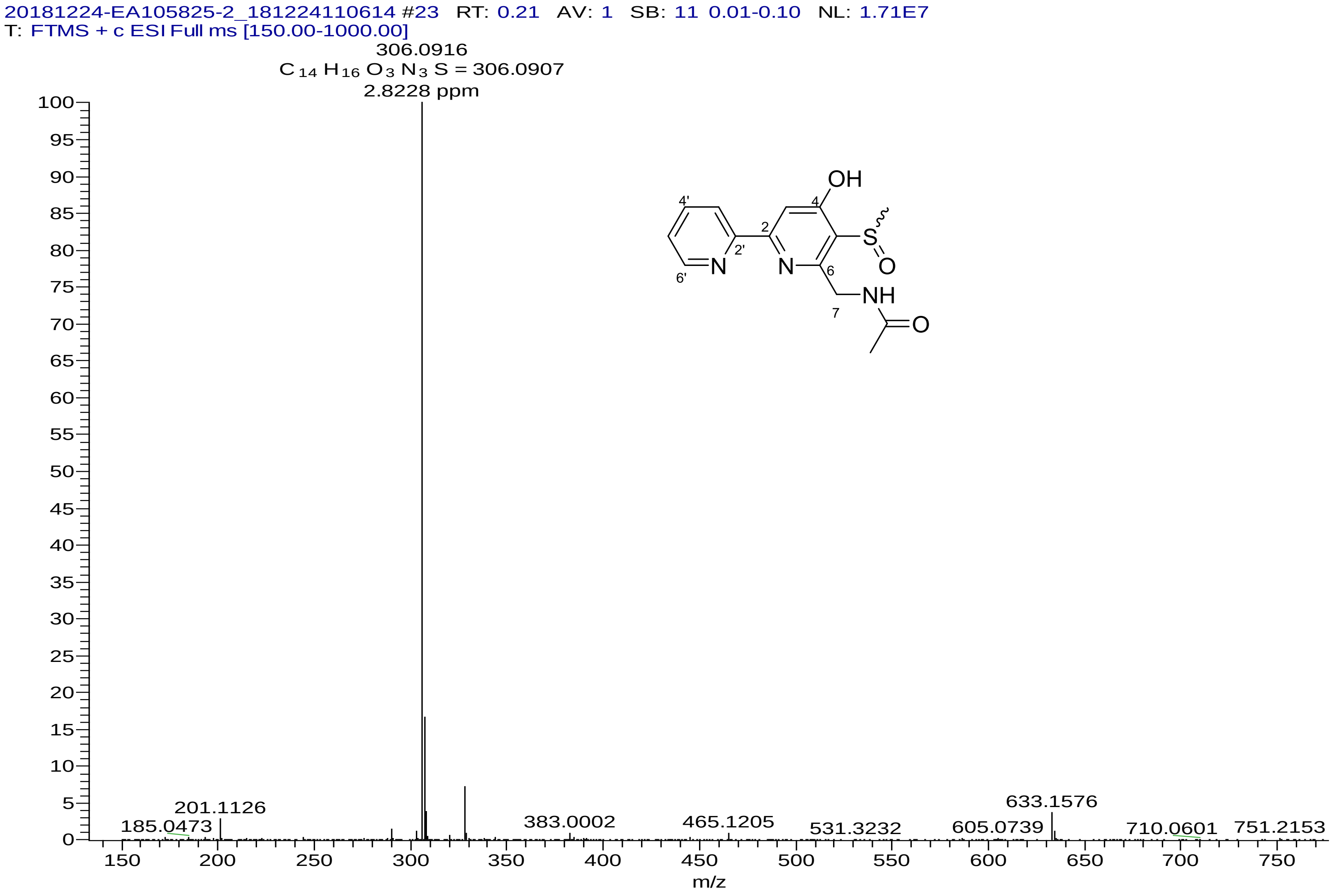

Fig. S12. ^{1}H NMR spectrum of (±)-**2** in DMSO-d_6.

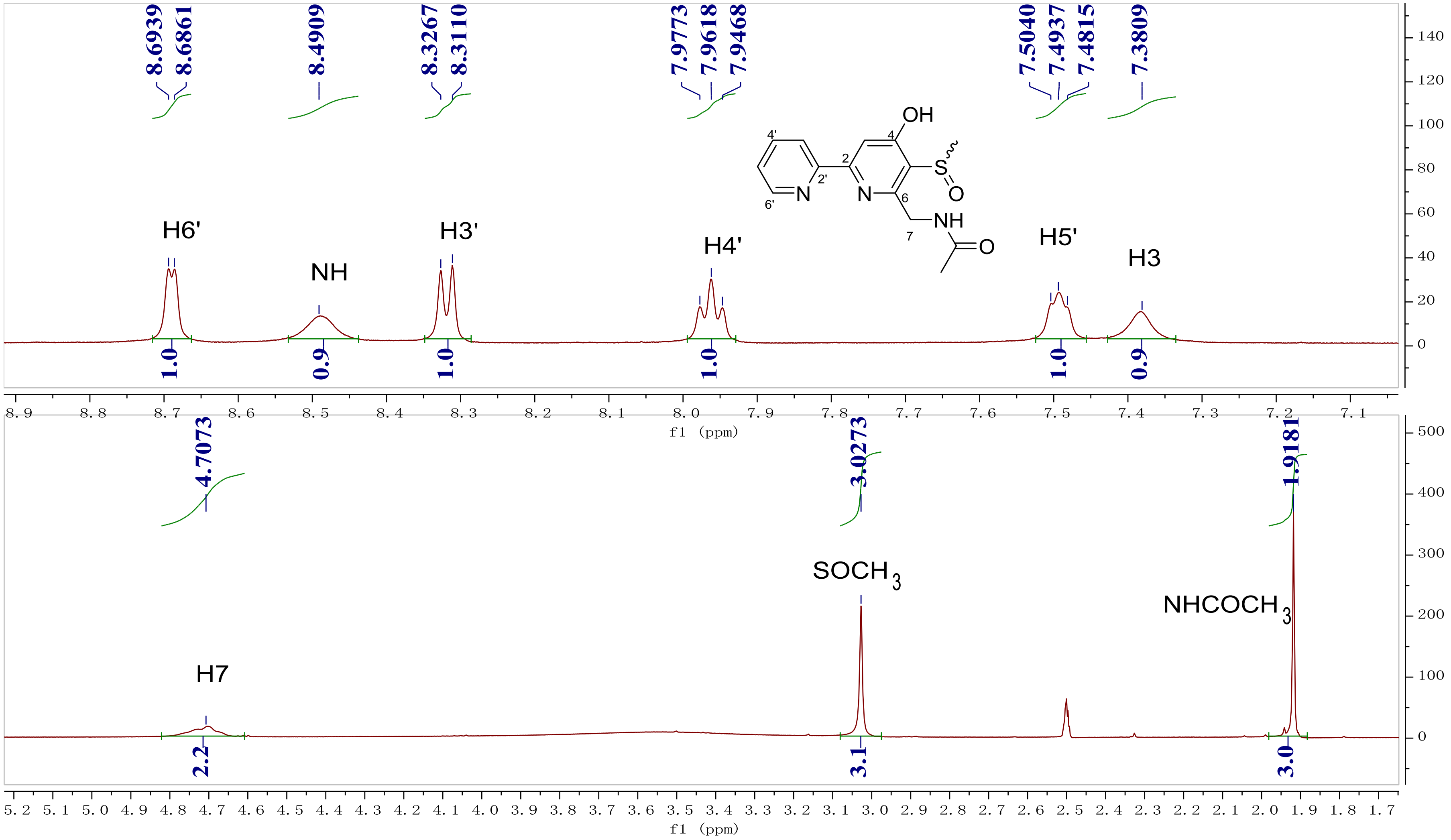

Fig. S13. Quantitative DEPT (Q-DEPT) NMR spectrum of (±)-**2** in DMSO-d_6.

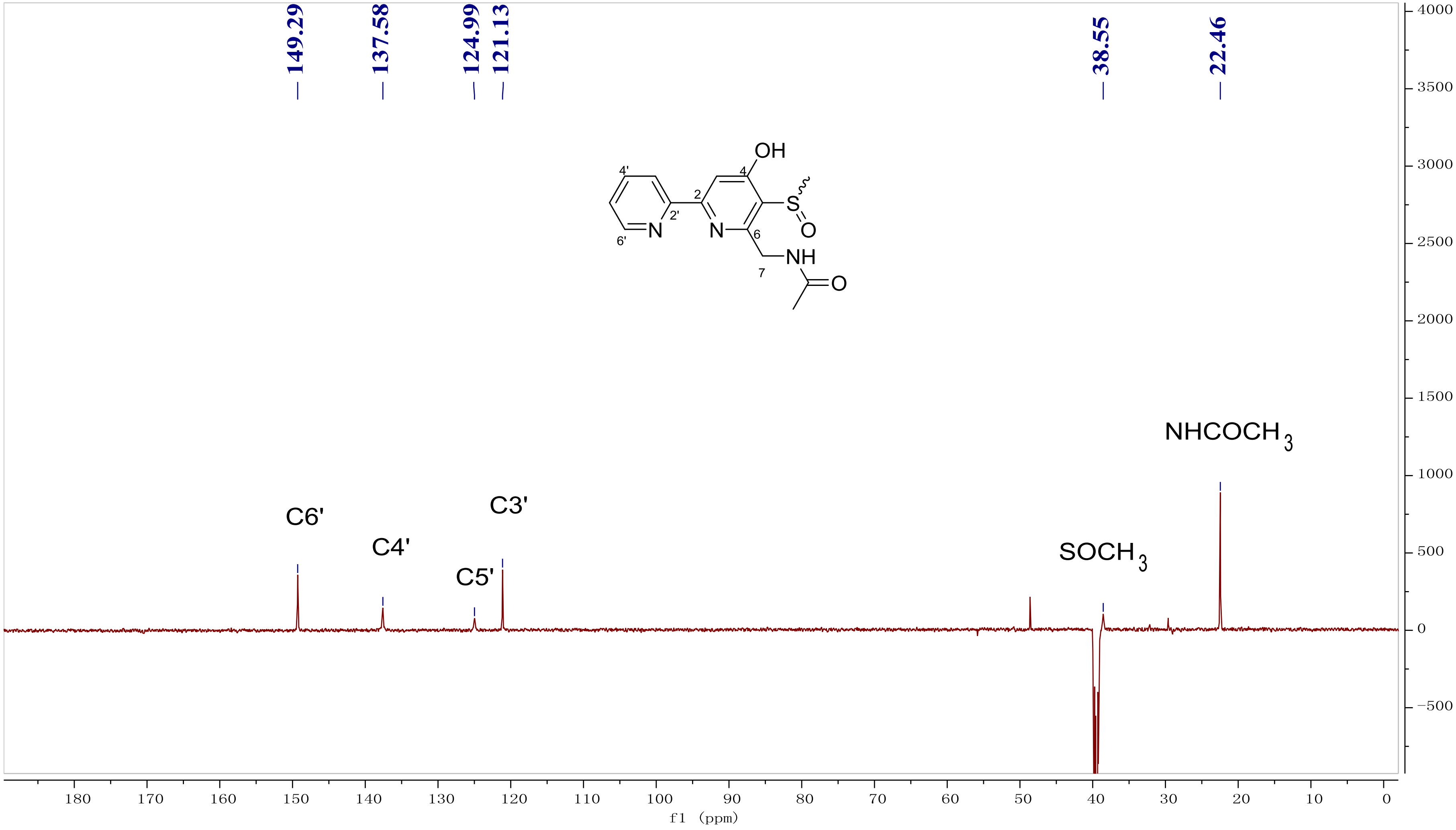

Fig. S14. HSQC spectrum of (±)-**2** in DMSO-d_6.

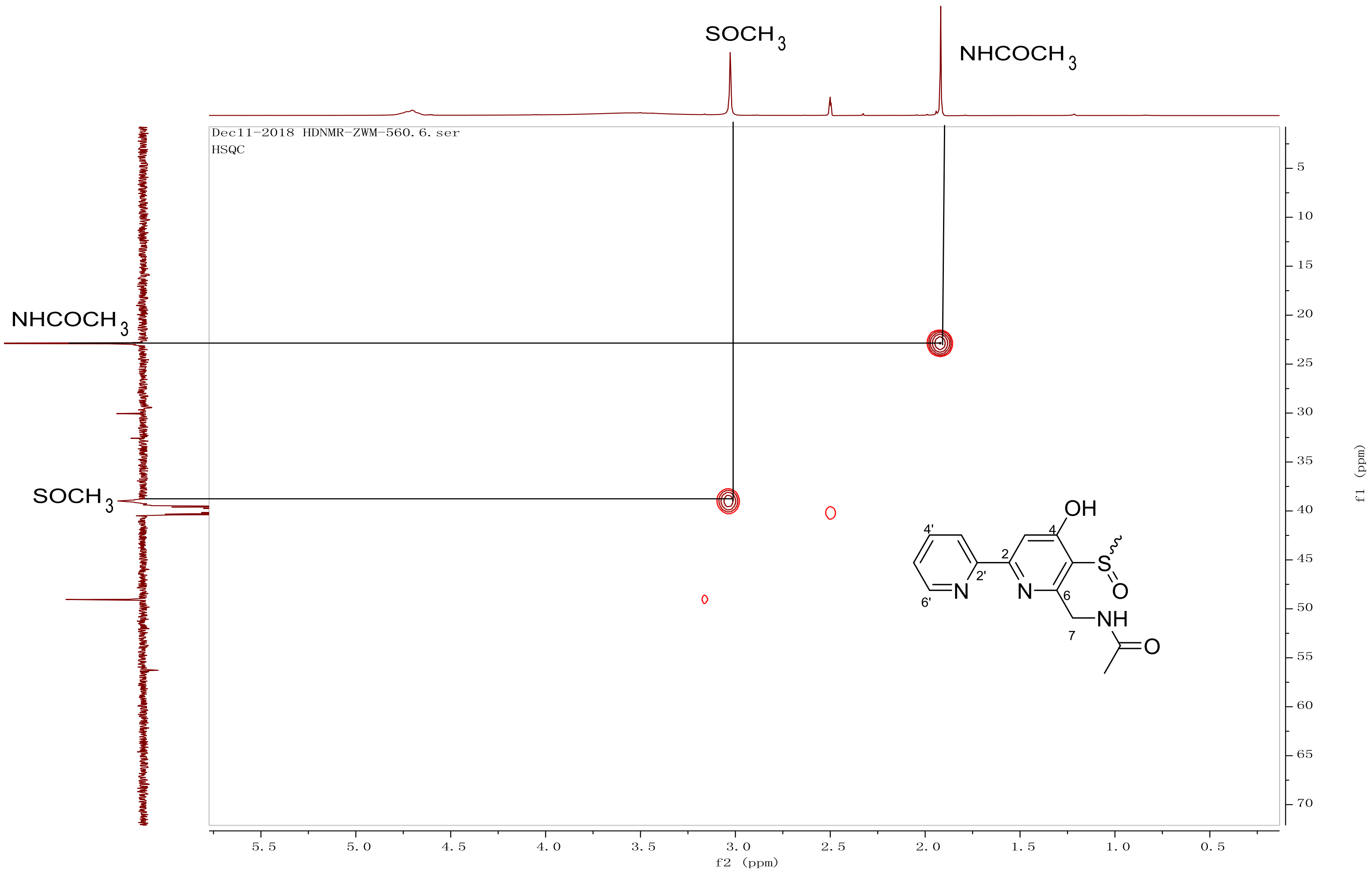

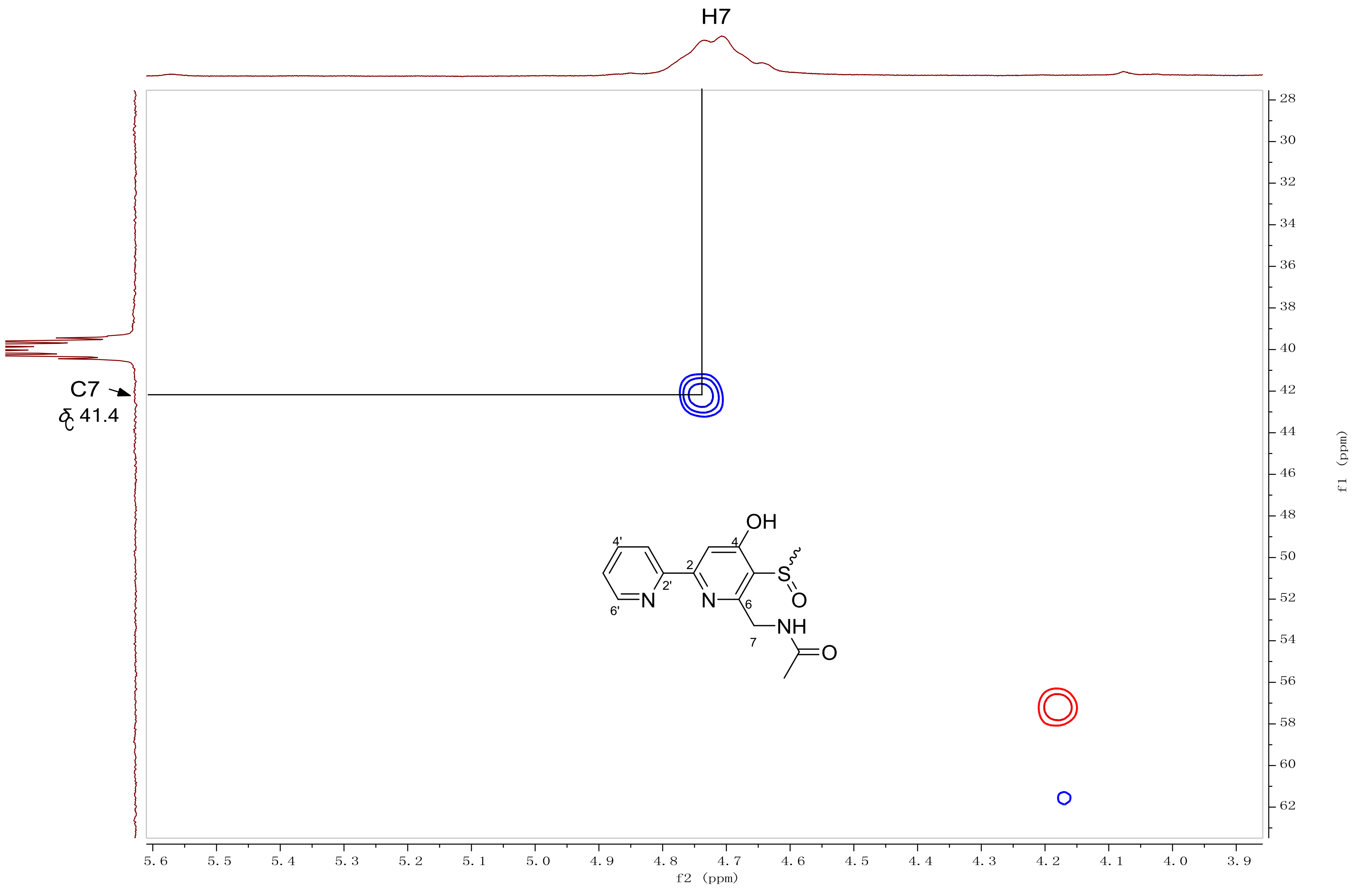
H7
C7
δC 41.4
f1 (ppm)
f2 (ppm)
OH
S
O
N
NH
O
4'
2'
6'
2
4
6
7

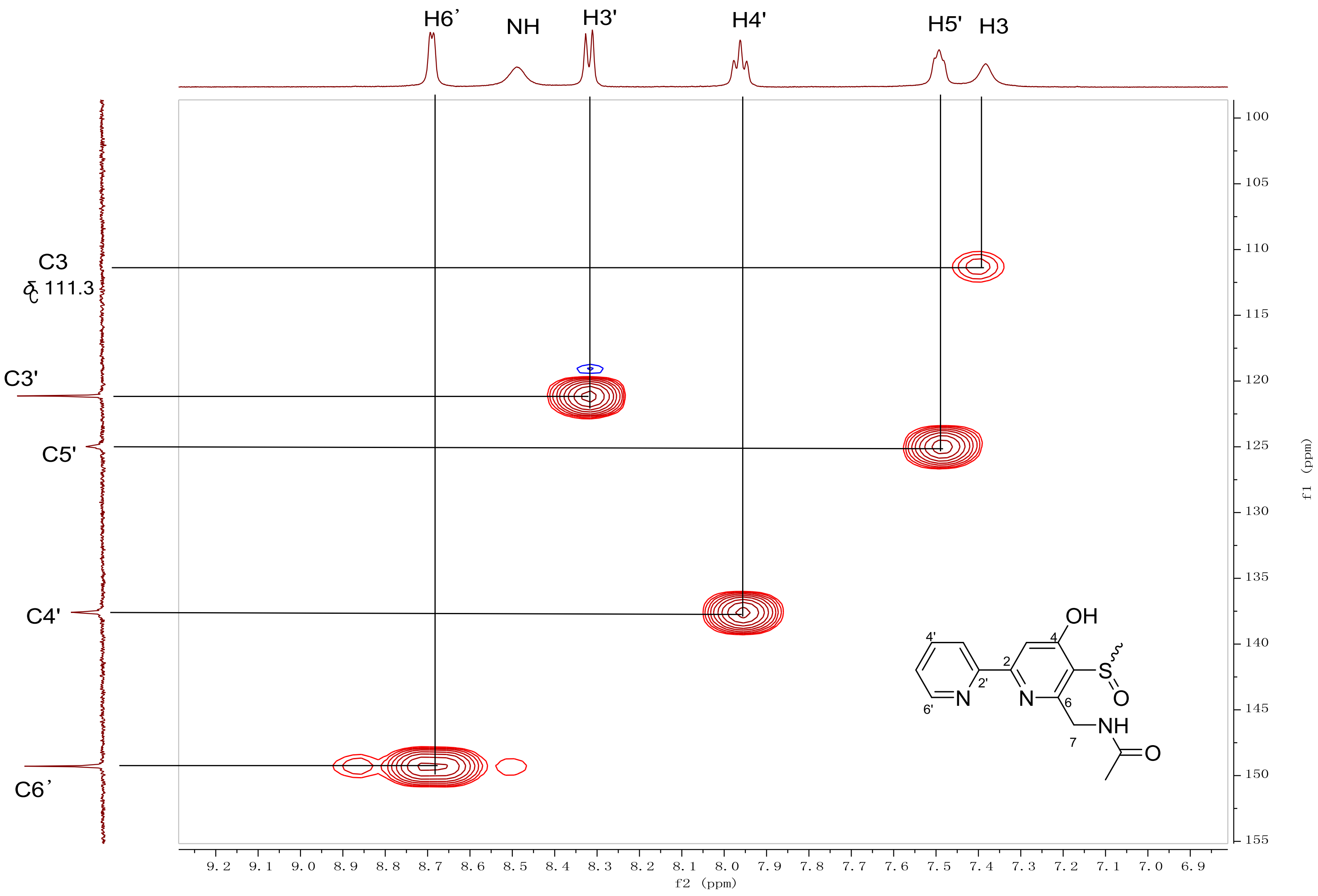

H6'
NH
H3'
H4'
H5'
H3
C3
δC 111.3
C3'
C5'
C4'
C6'
OH
4'
4
2
2'
N
N
6
6'
S
O
NH
7
O
f2 (ppm)
f1 (ppm)

Fig. S15. $^1H-^1H$ COSY spectrum of (±)-**2** in DMSO-d_6.

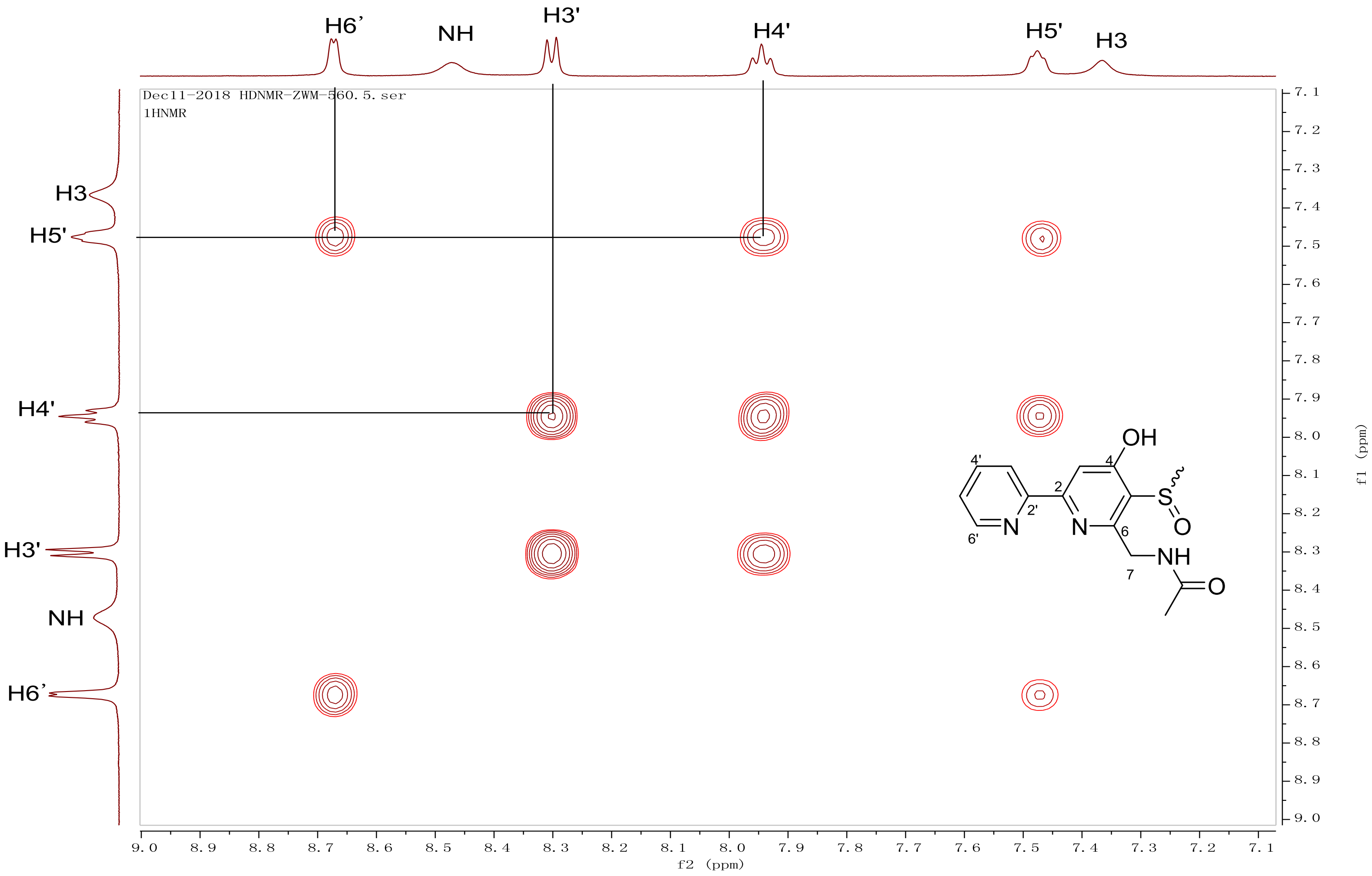

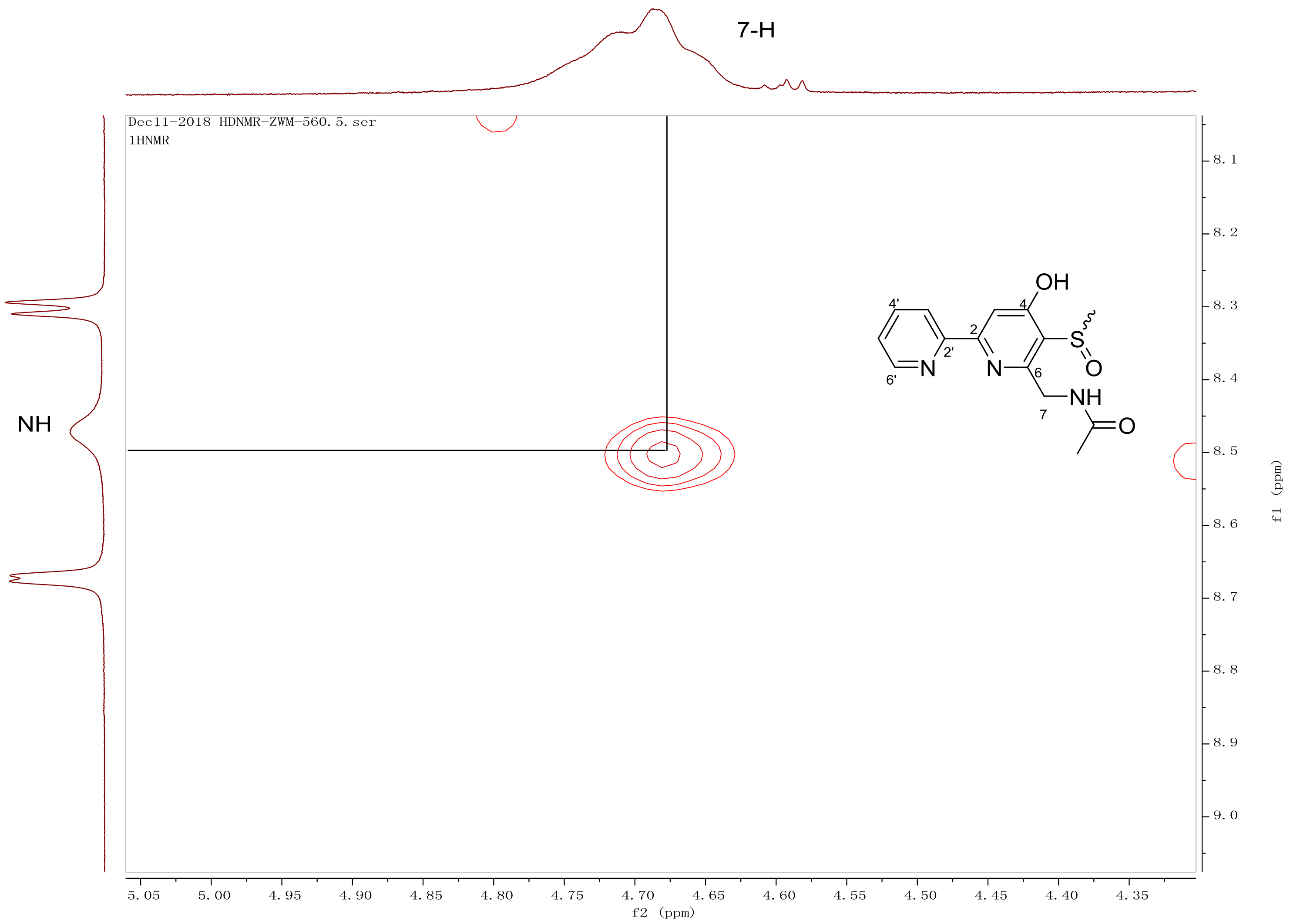
7-H
NH
Dec11-2018 HDNMR-ZWM-560.5.ser
1HNMR
OH
4'
4
2
2'
6'
N
N
6
S
O
NH
7
O
f2 (ppm)
f1 (ppm)

Fig. S16. HMBC spectrum of (±)-**2** in DMSO-d_6.

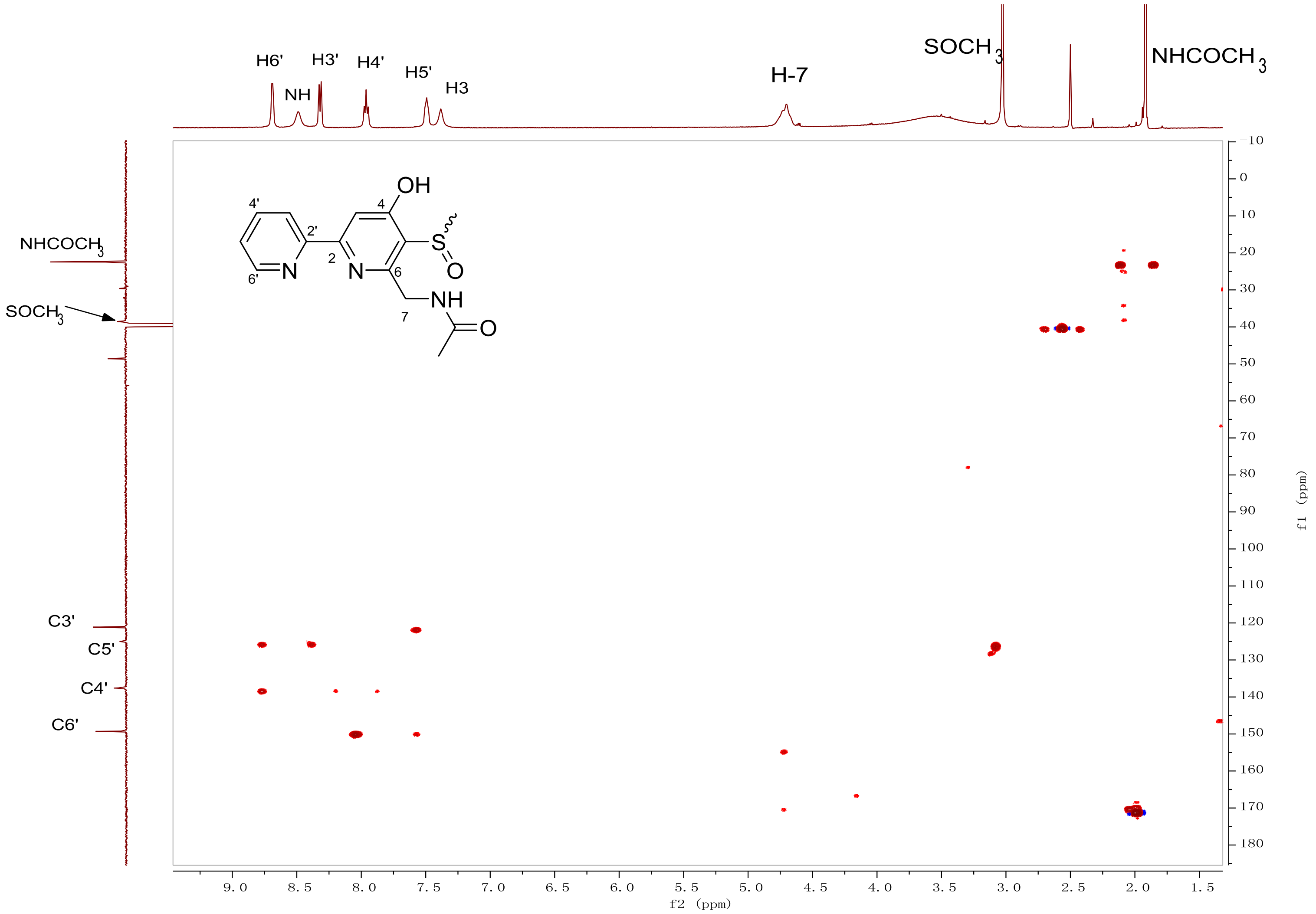

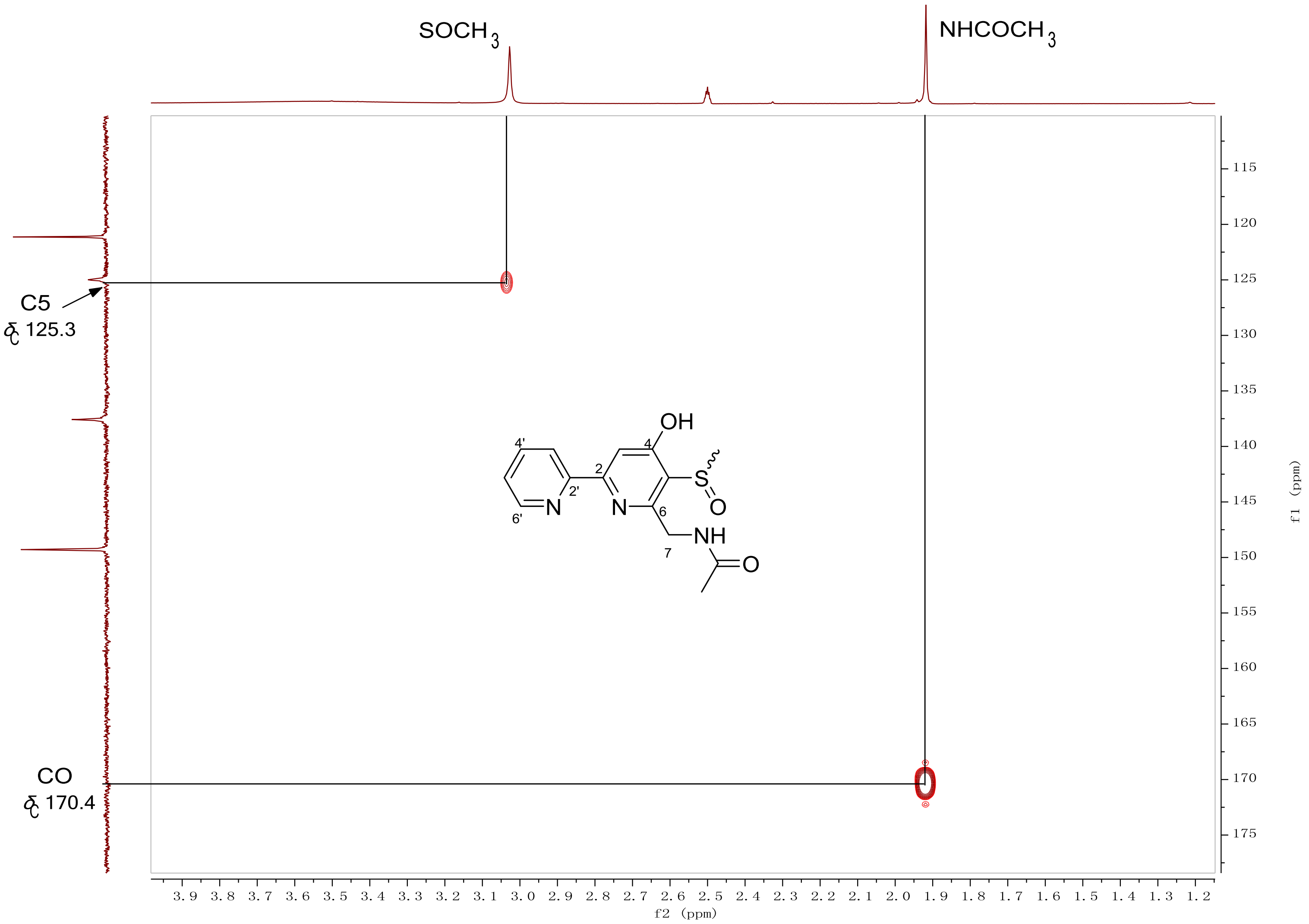
SOCH3
NHCOCH3
C5
δC 125.3
CO
δC 170.4
OH
4'
2
4
2'
S
O
N
N
6'
6
NH
7
O
f2 (ppm)
f1 (ppm)

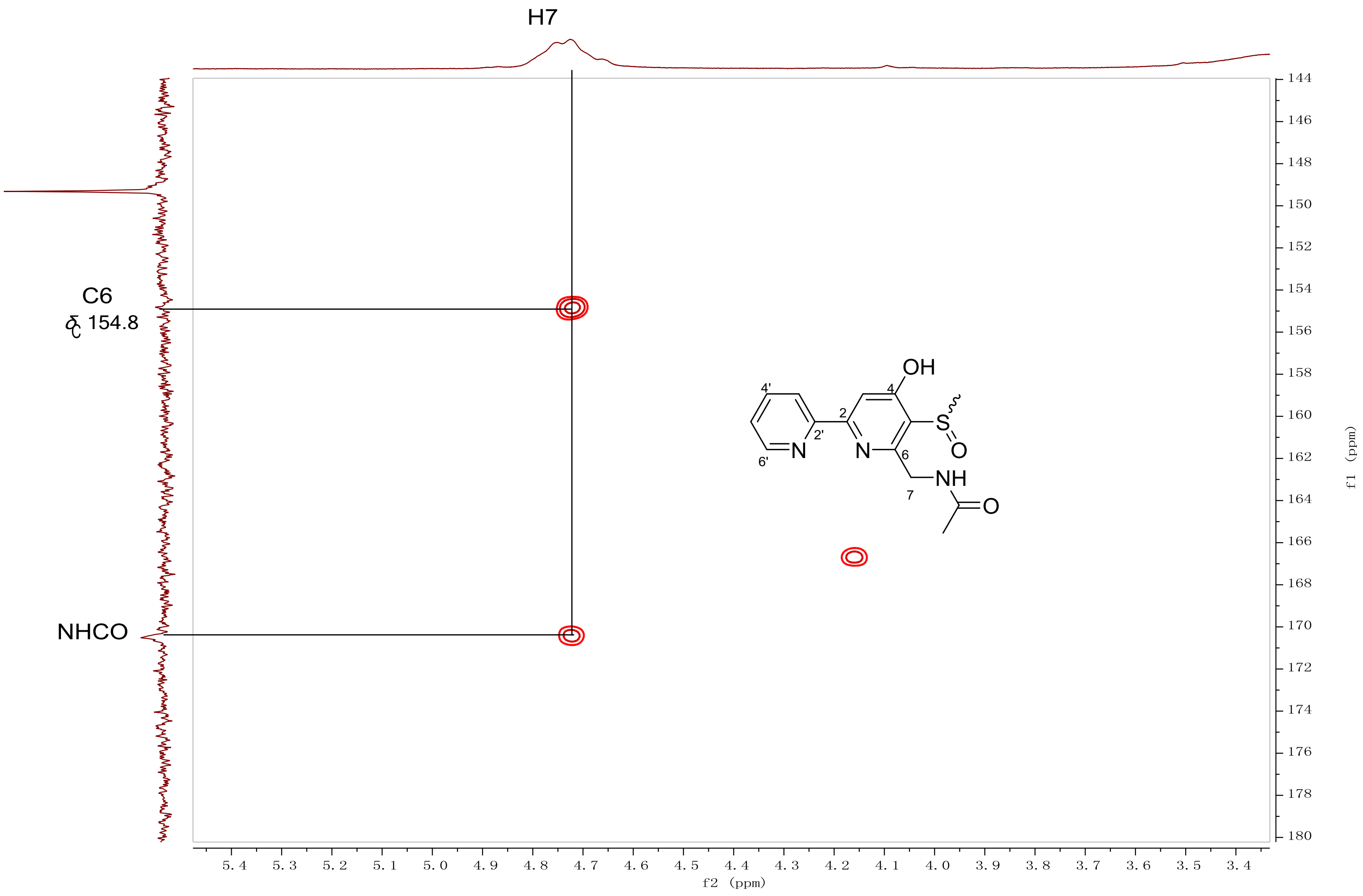
H7
C6
δC 154.8
NHCO
OH
4'
4
2
2'
N
S
O
6'
6
NH
7
O
f2 (ppm)
f1 (ppm)

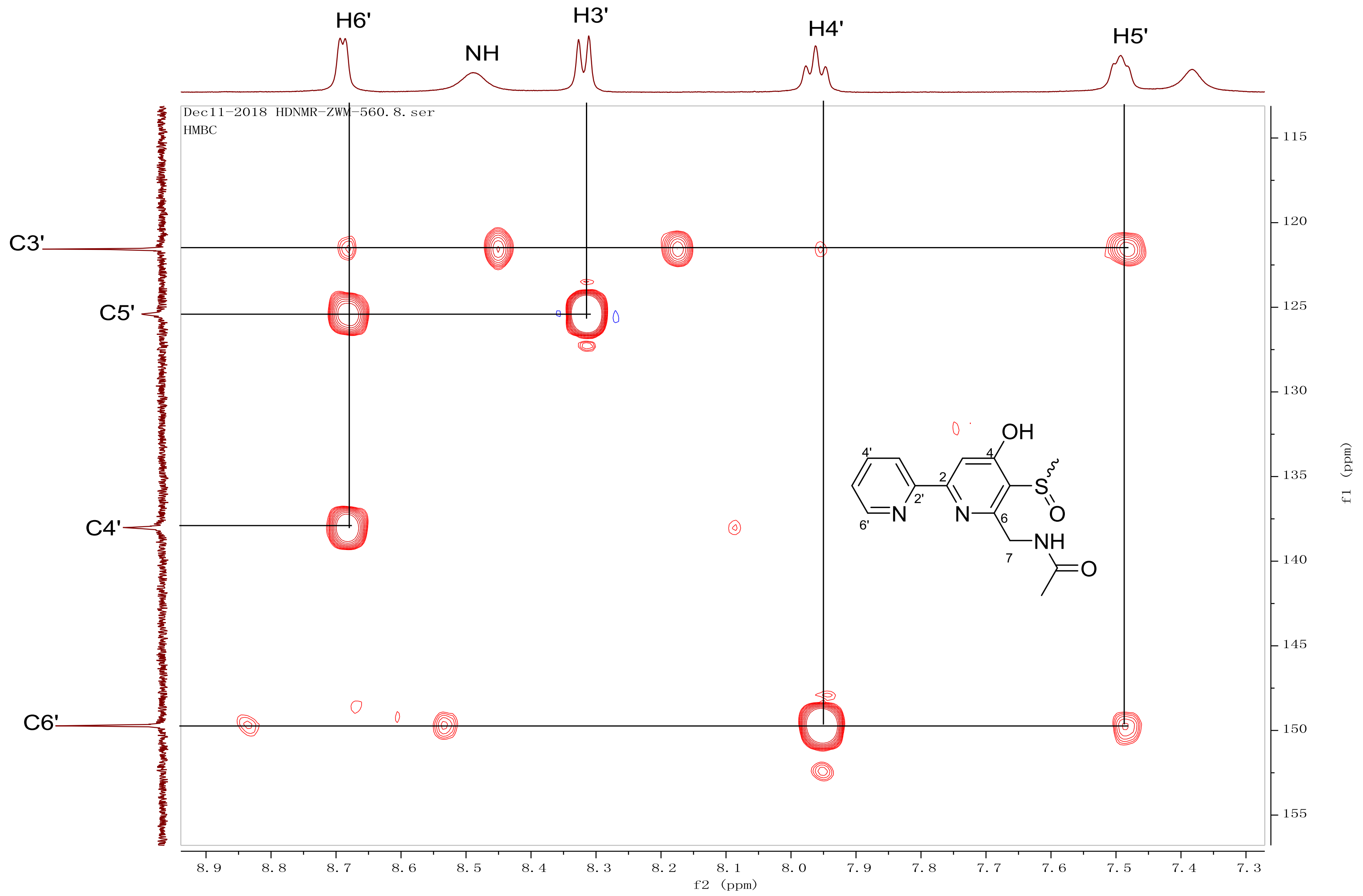

H6'
NH
H3'
H4'
H5'
Dec11-2018 HDNMR-ZWM-560.8.ser
HMBC
C3'
C5'
C4'
C6'
OH
4'
4
2
2'
S
O
N
N
6
6'
NH
7
O
f2 (ppm)
f1 (ppm)

Fig. S17. ^{1}H NMR spectrum of (±)-**2** in CD_3OD.

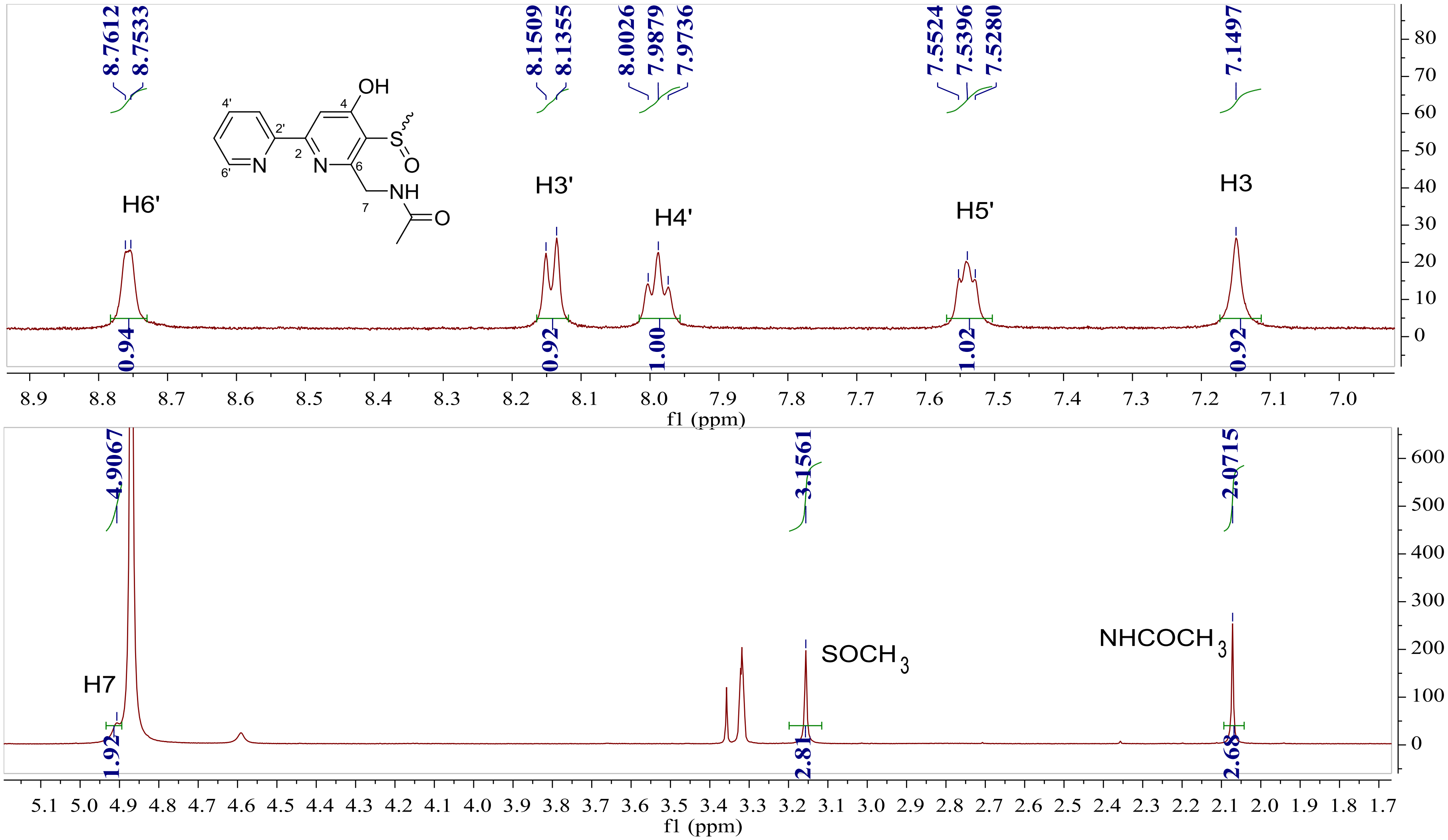

Fig. S18. ^{13}C NMR spectrum of (±)-**2** in CD_3OD.

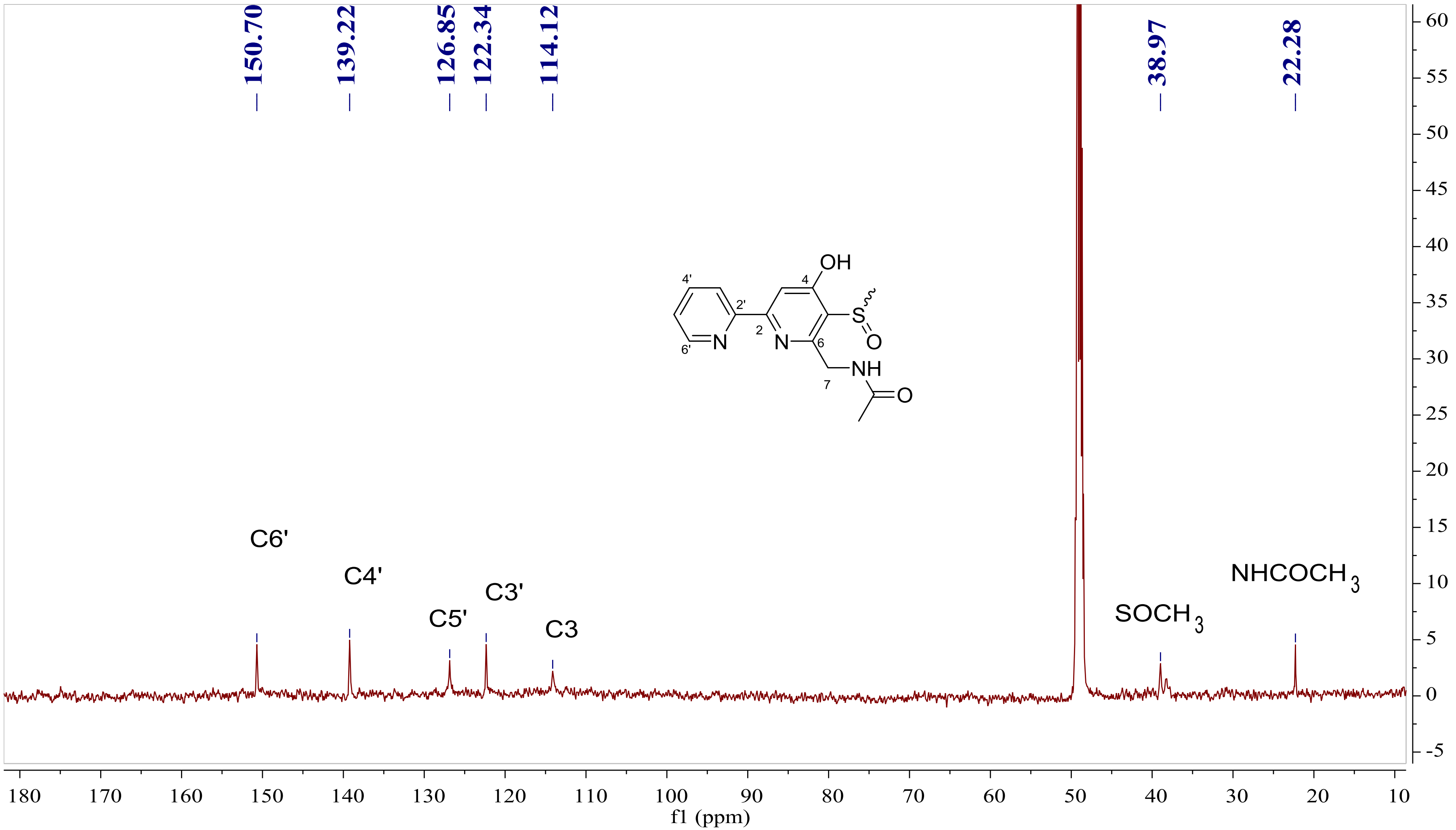

Fig. S19. HSQC spectrum of (±)-**2** in CD_3OD.

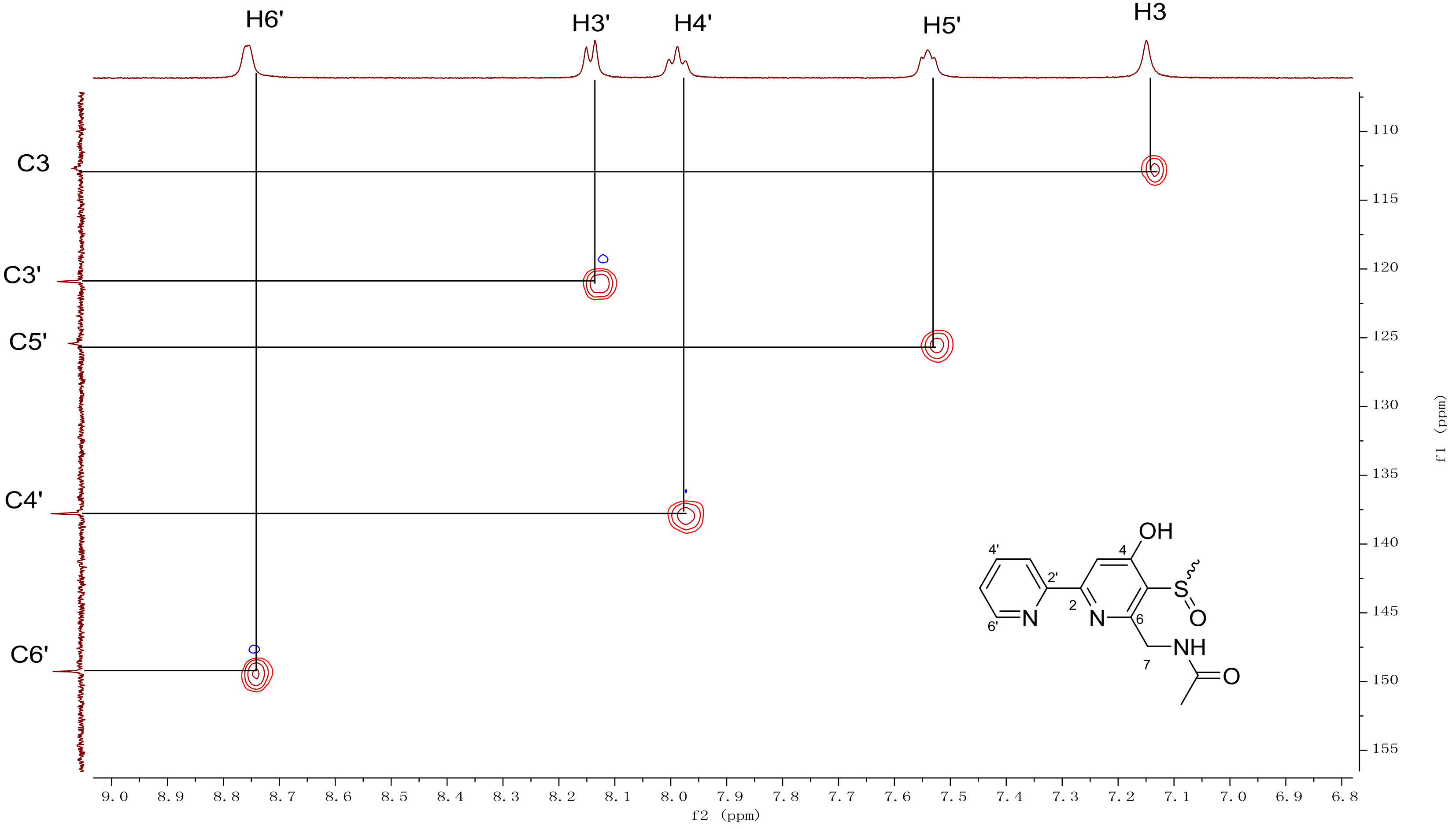

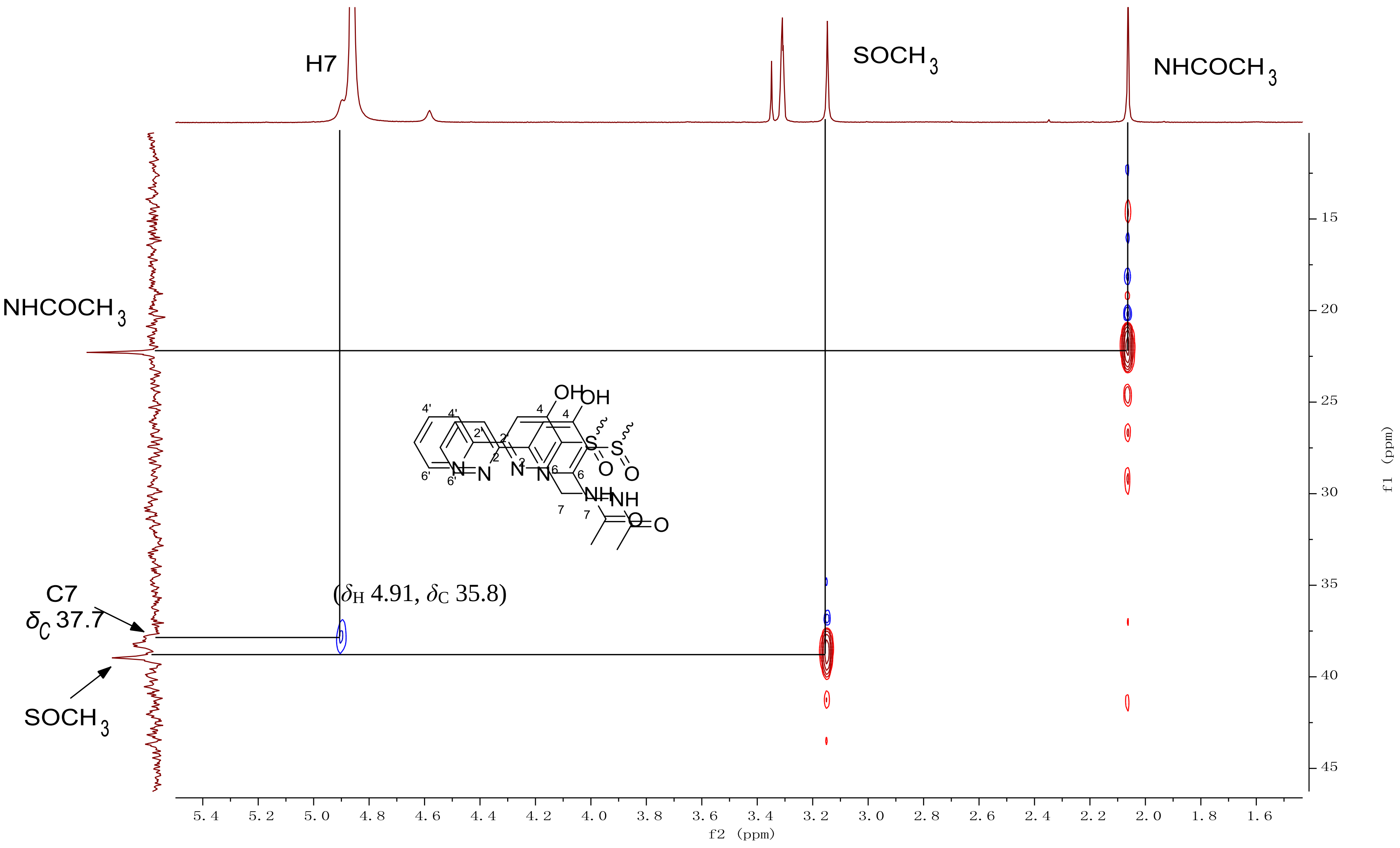
H7
SOCH3
NHCOCH3
NHCOCH3
C7
δC 37.7
SOCH3
(δH 4.91, δC 35.8)
OH
S
O
N
NH
O
f2 (ppm)
f1 (ppm)

Fig. S20. ^{1}H-^{1}H COSY spectrum of (±)-**2** in CD_3OD.

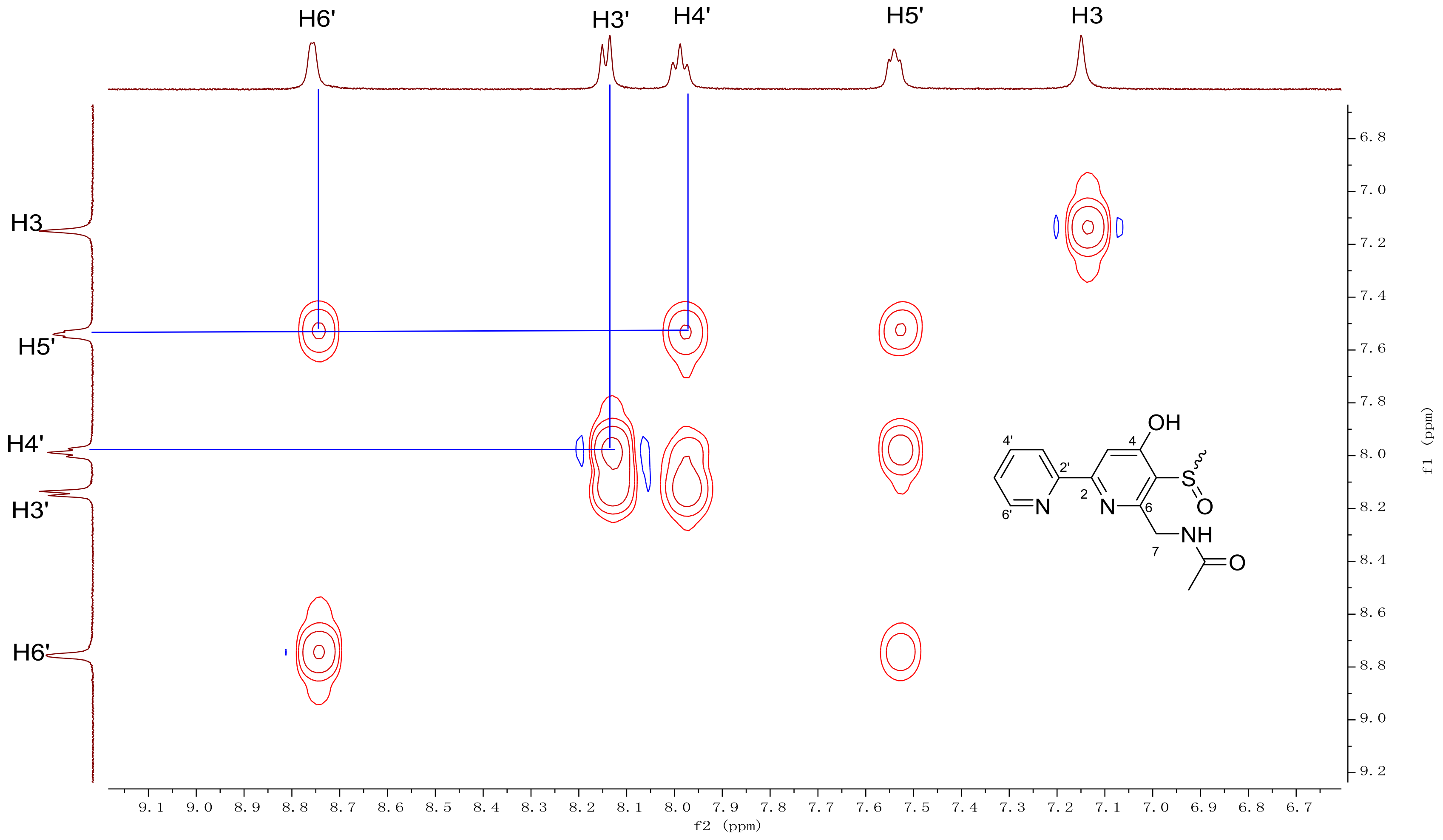

Fig. S21. HMBC spectrum of (±)-**2** in CD_3OD.

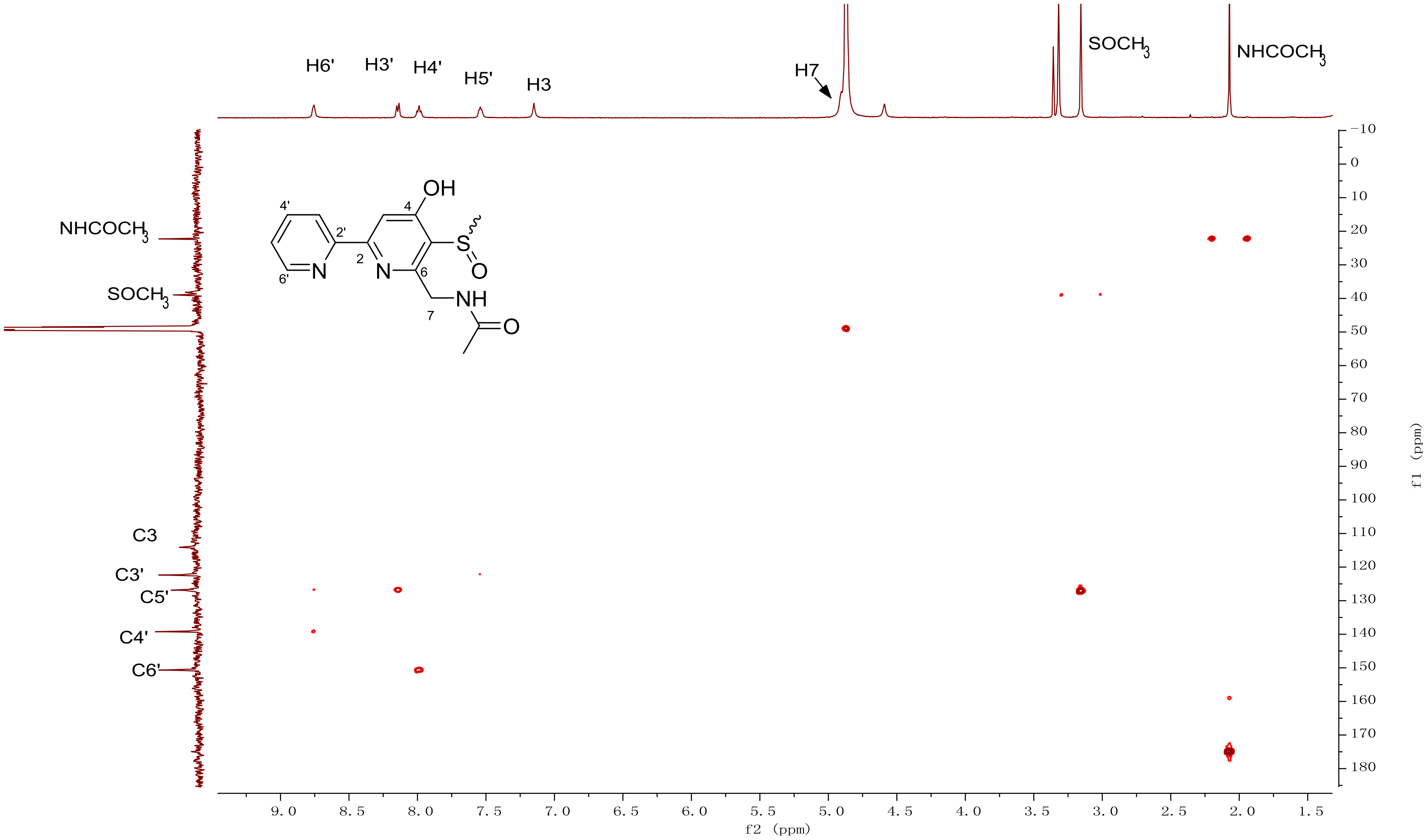

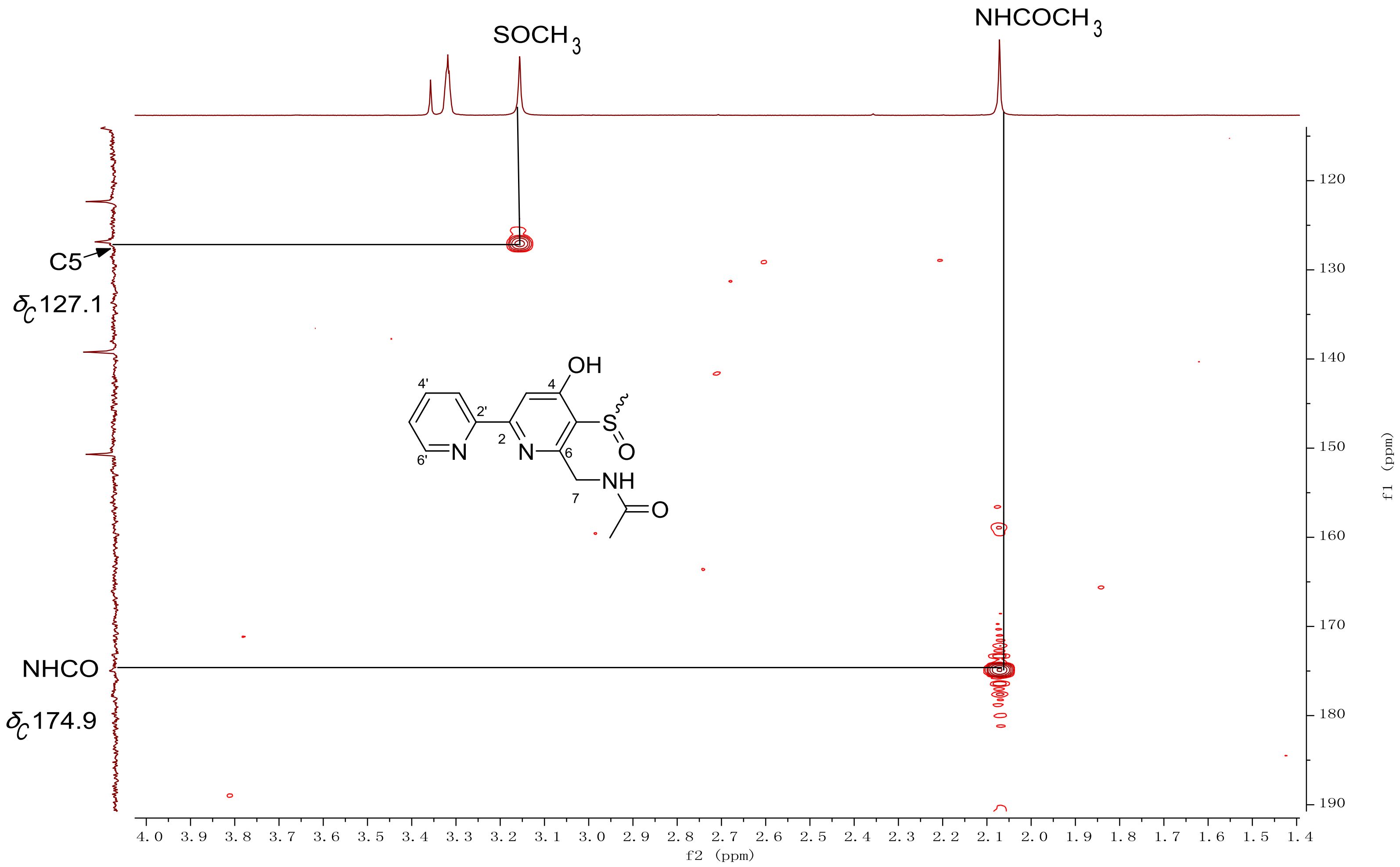
SOCH3
NHCOCH3
C5
δC 127.1
NHCO
δC 174.9
OH
4
4'
2'
2
6
6'
N
S
O
NH
7
f2 (ppm)
f1 (ppm)

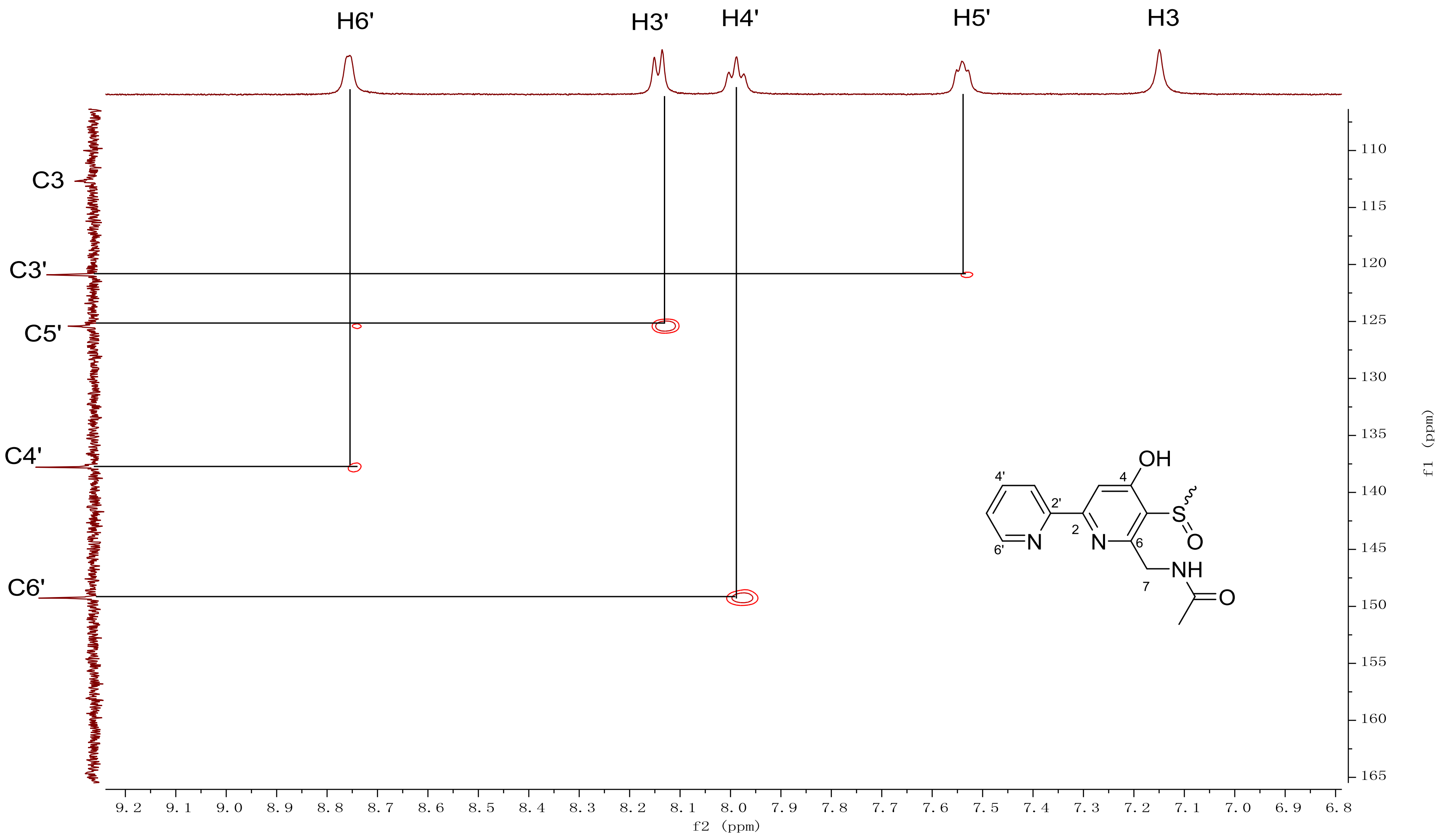

H6'
H3'
H4'
H5'
H3
C3
C3'
C5'
C4'
C6'
f2 (ppm)
f1 (ppm)
OH
NH
O
S
N

Fig. S22. ESIMS spectrum of synthetic (±)-**2**.

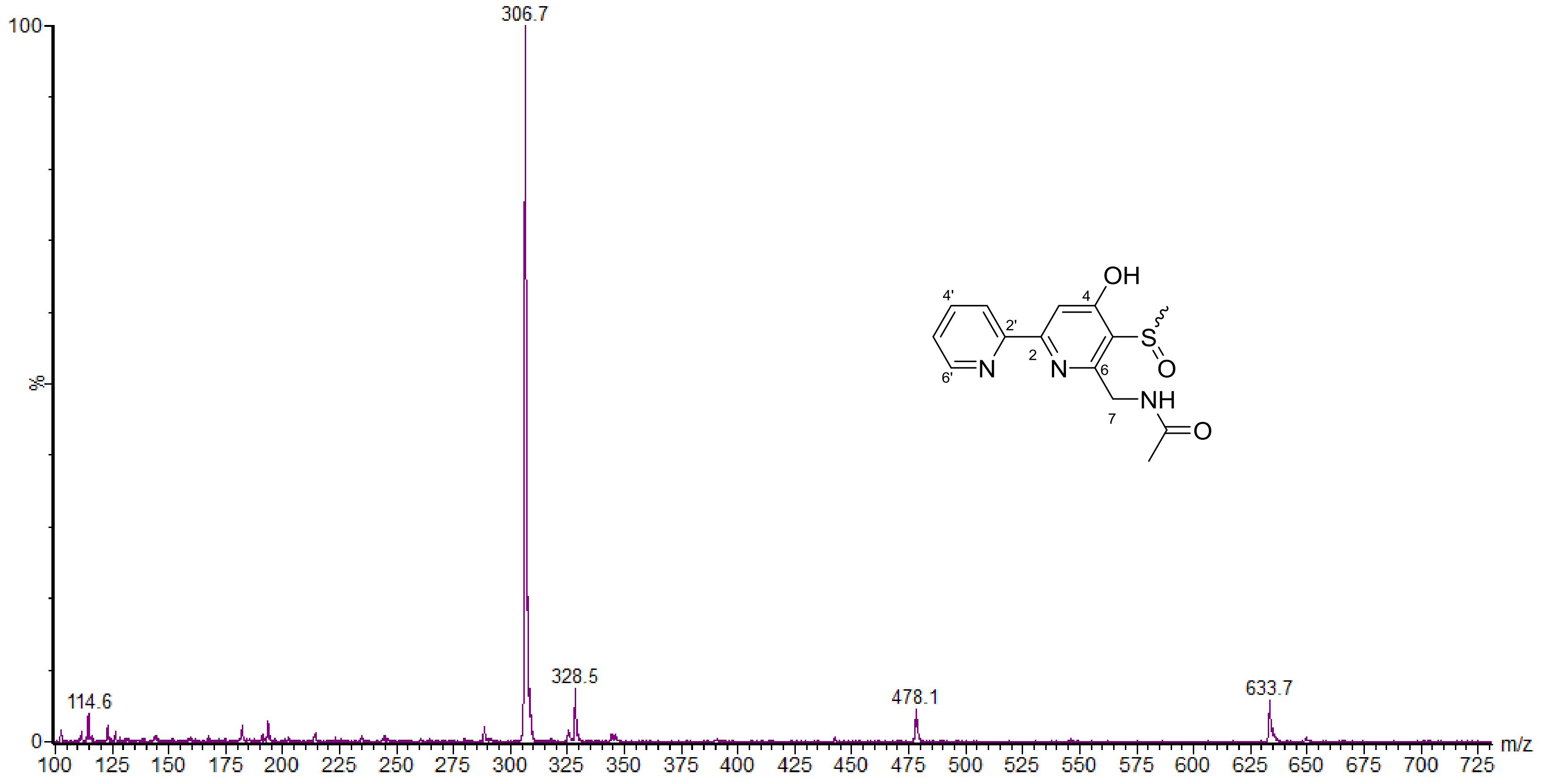

Fig. S23. ^{1}H NMR spectrum of synthetic (±)-**2** in DMSO-d_6.

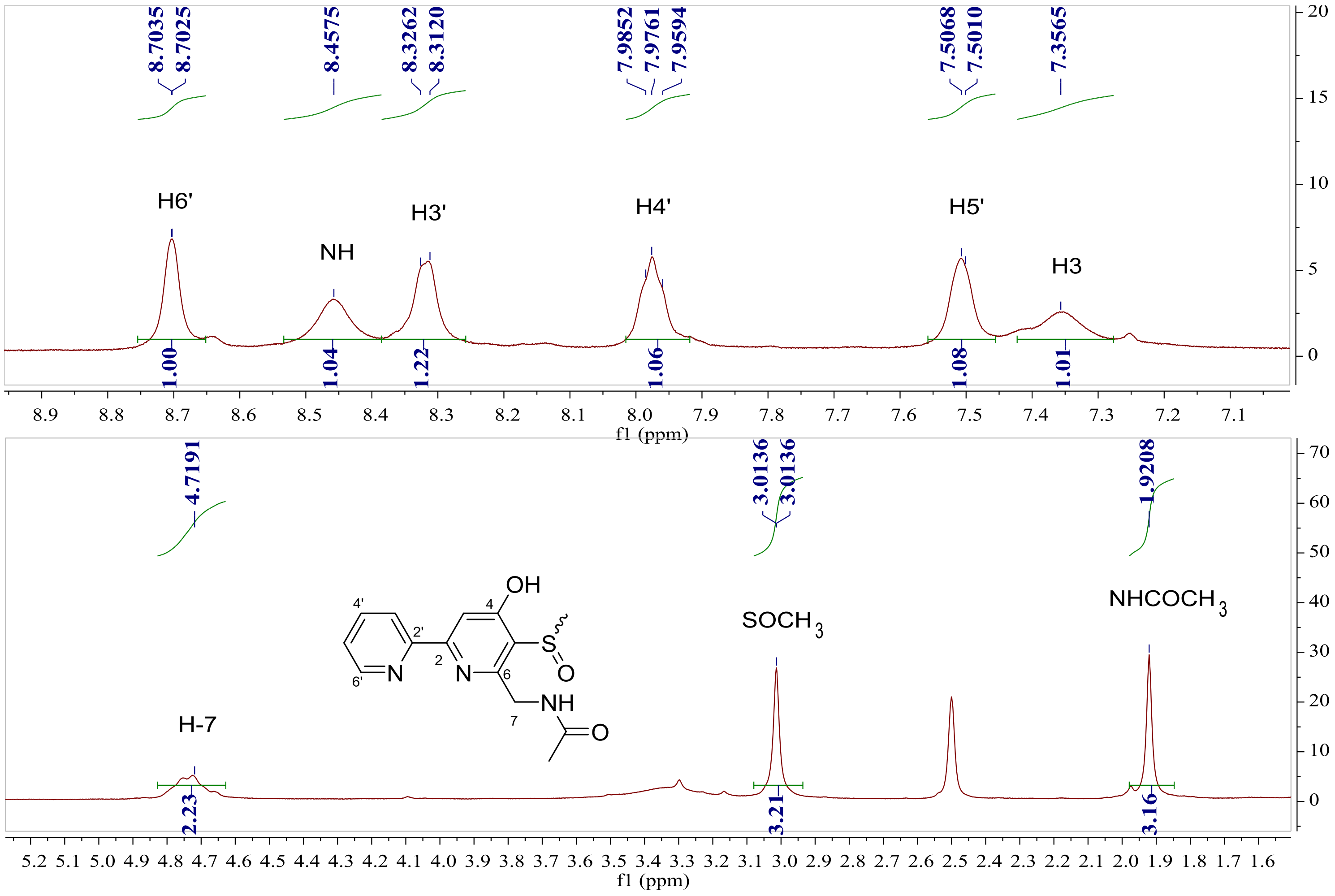

Fig. S24. ^{13}C NMR spectrum of synthetic (±)-**2** in DMSO-d_6.

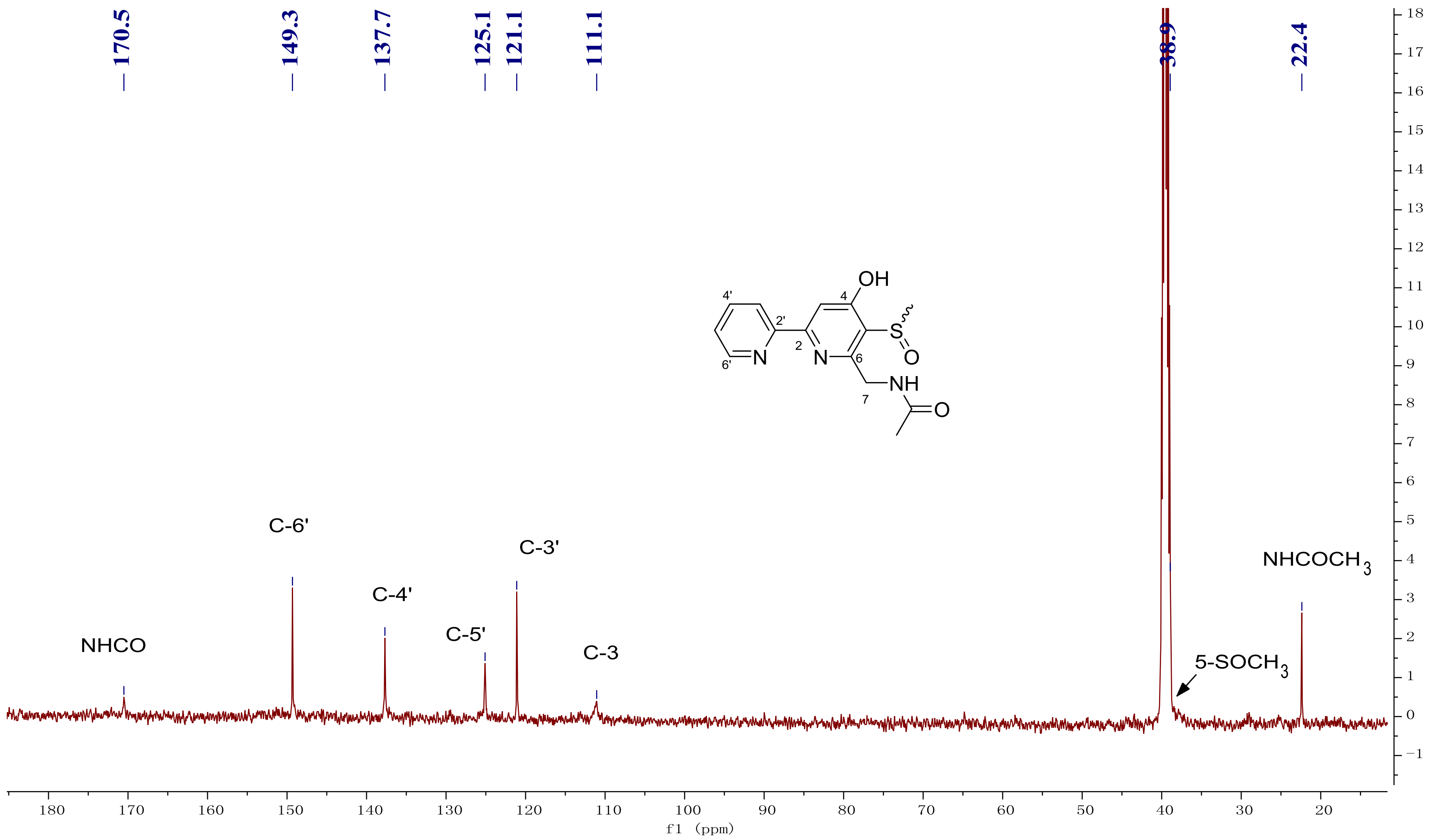

Fig. S25. HRESIMS spectrum of (±)-**2a**.

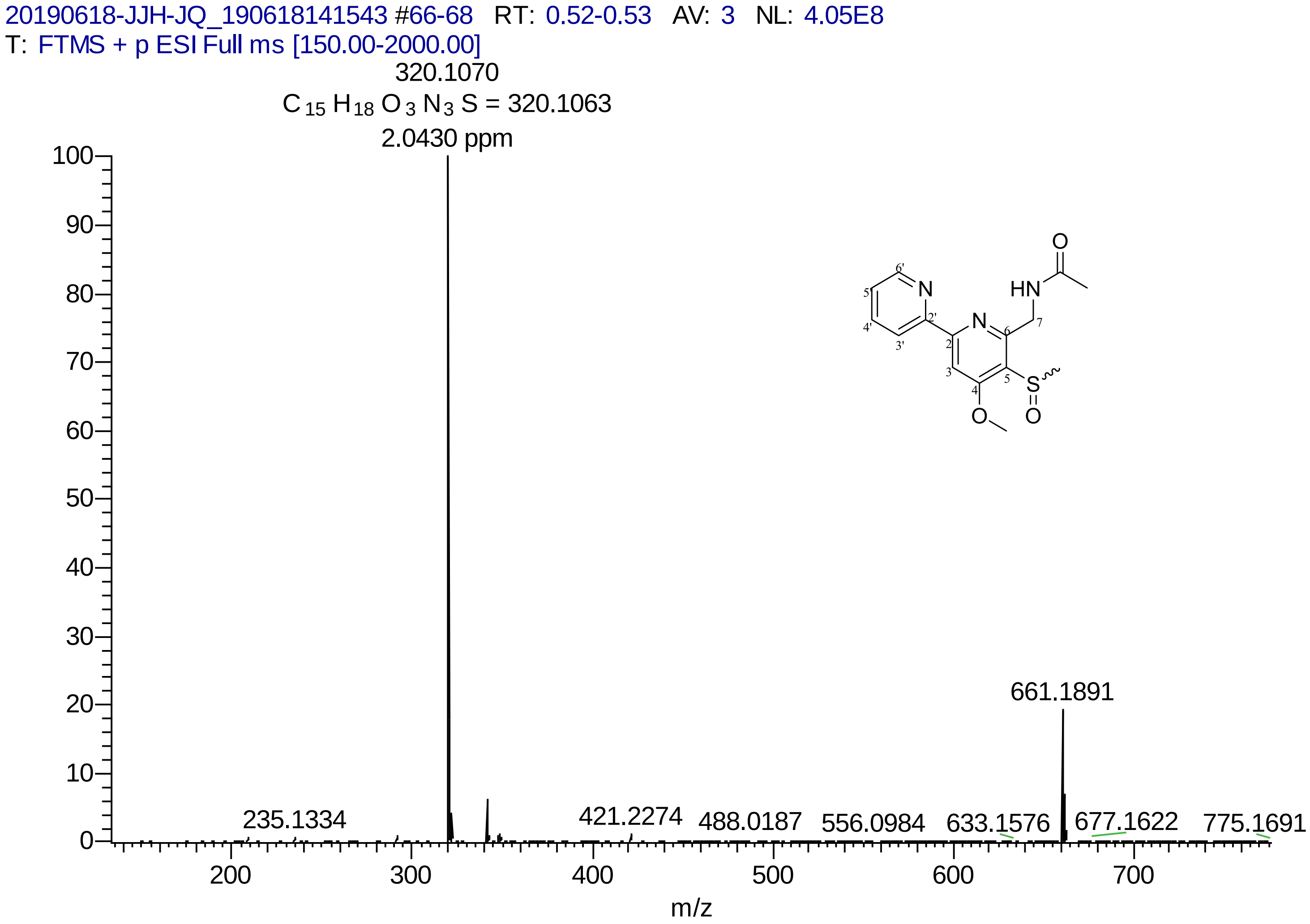

Fig. S26. ^{1}H NMR spectrum of (±)-**2a** in DMSO-d_6.

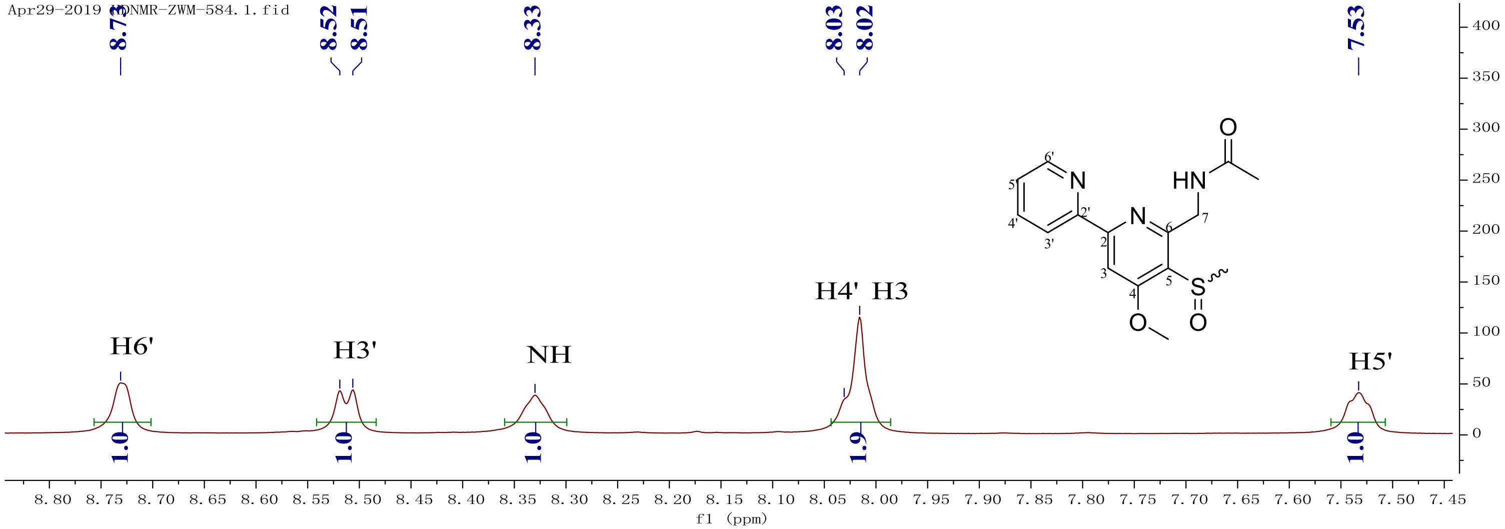

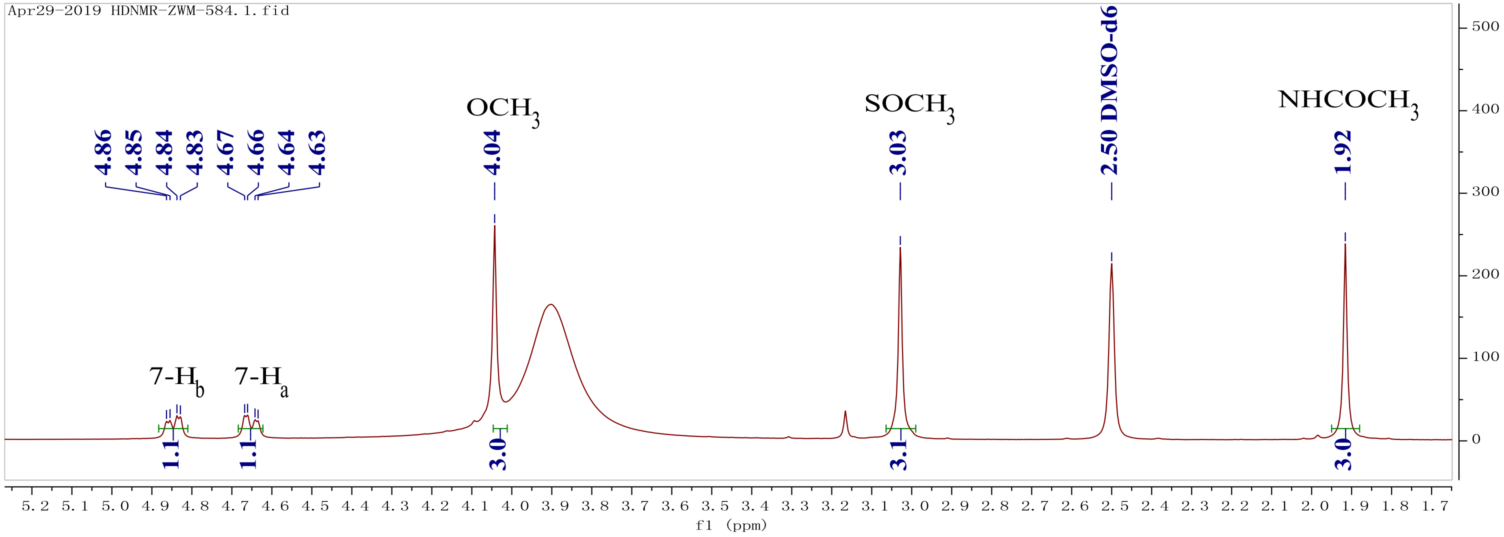

Fig. S27. ^{13}C NMR spectrum of (±)-**2a** in DMSO-d_6.

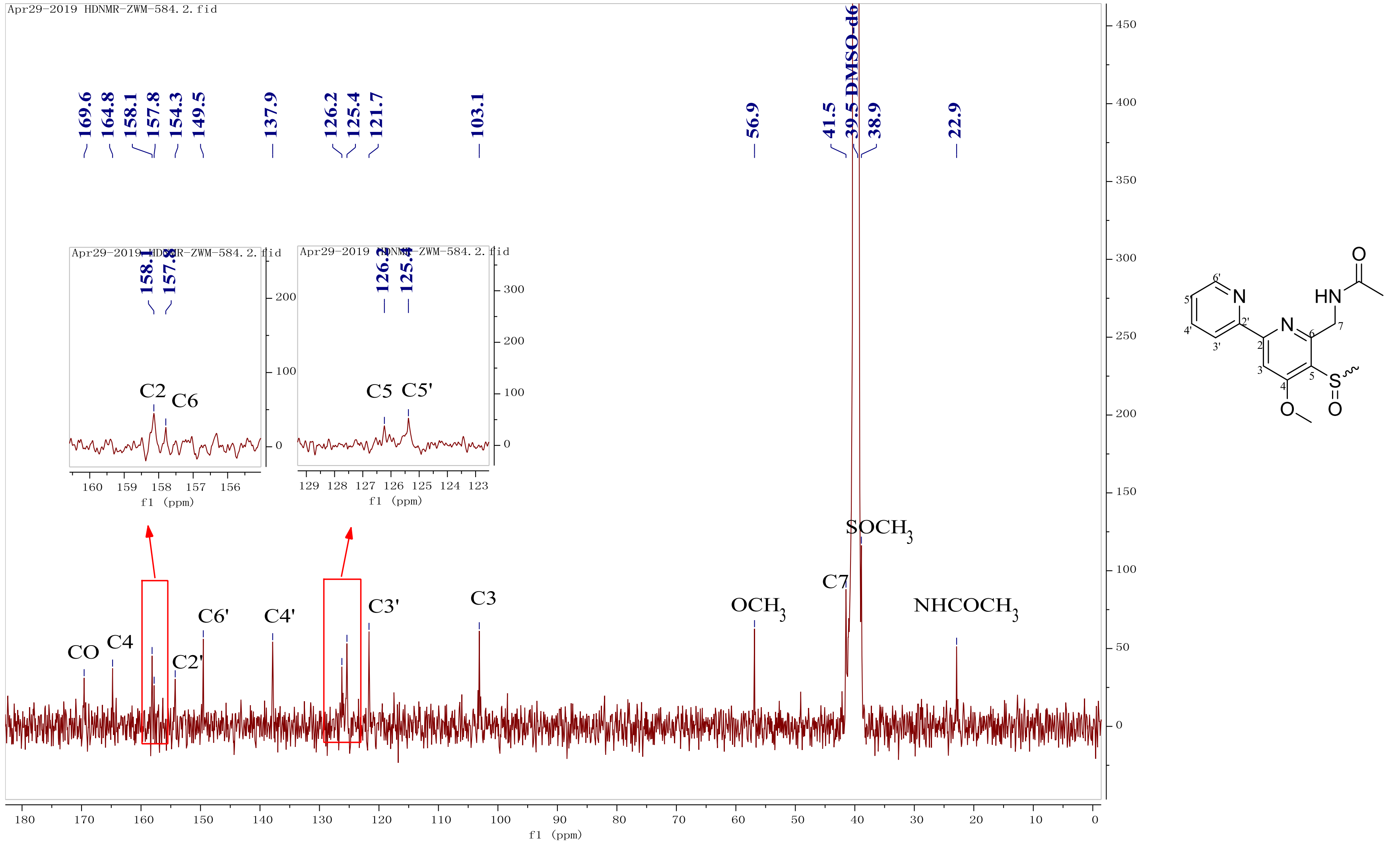

Fig. S28. HSQC spectrum of (±)-**2a** in DMSO-d_6

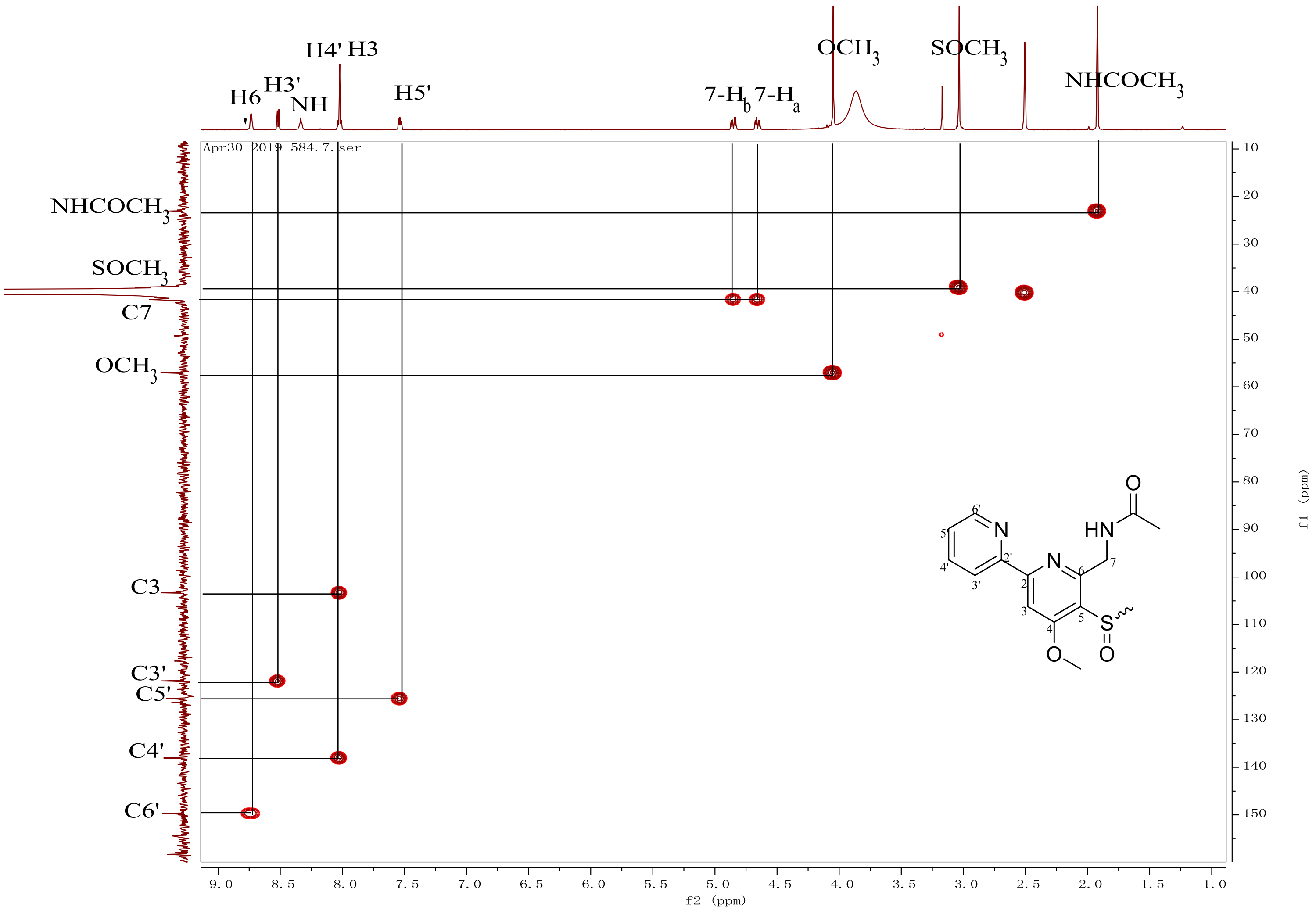

Fig. S29. ^{1}H-^{1}H COSY spectrum of (±)-**2a** in DMSO-d_6

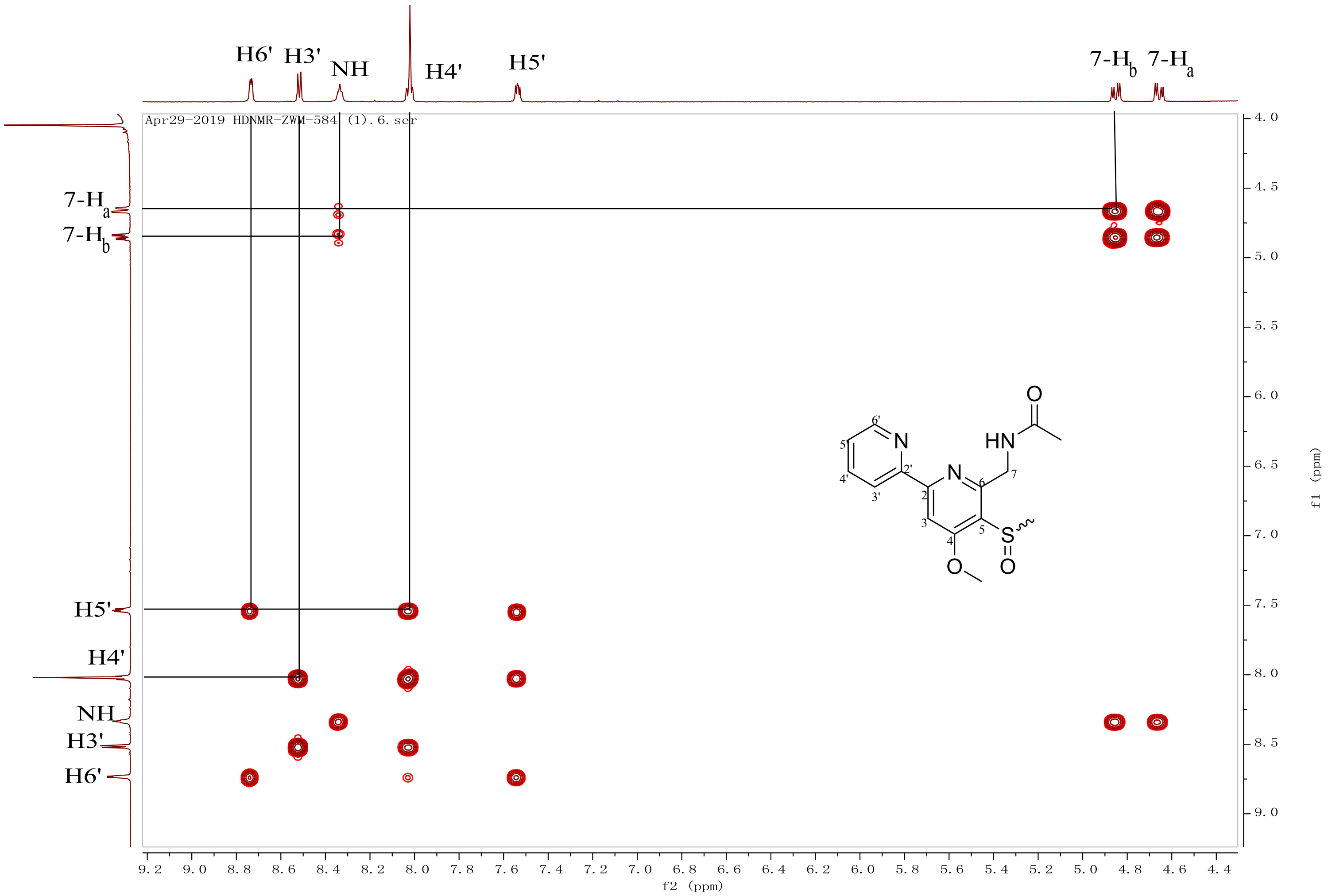

Fig. S30. HMBC spectrum of (±)-**2a** in DMSO-d_6

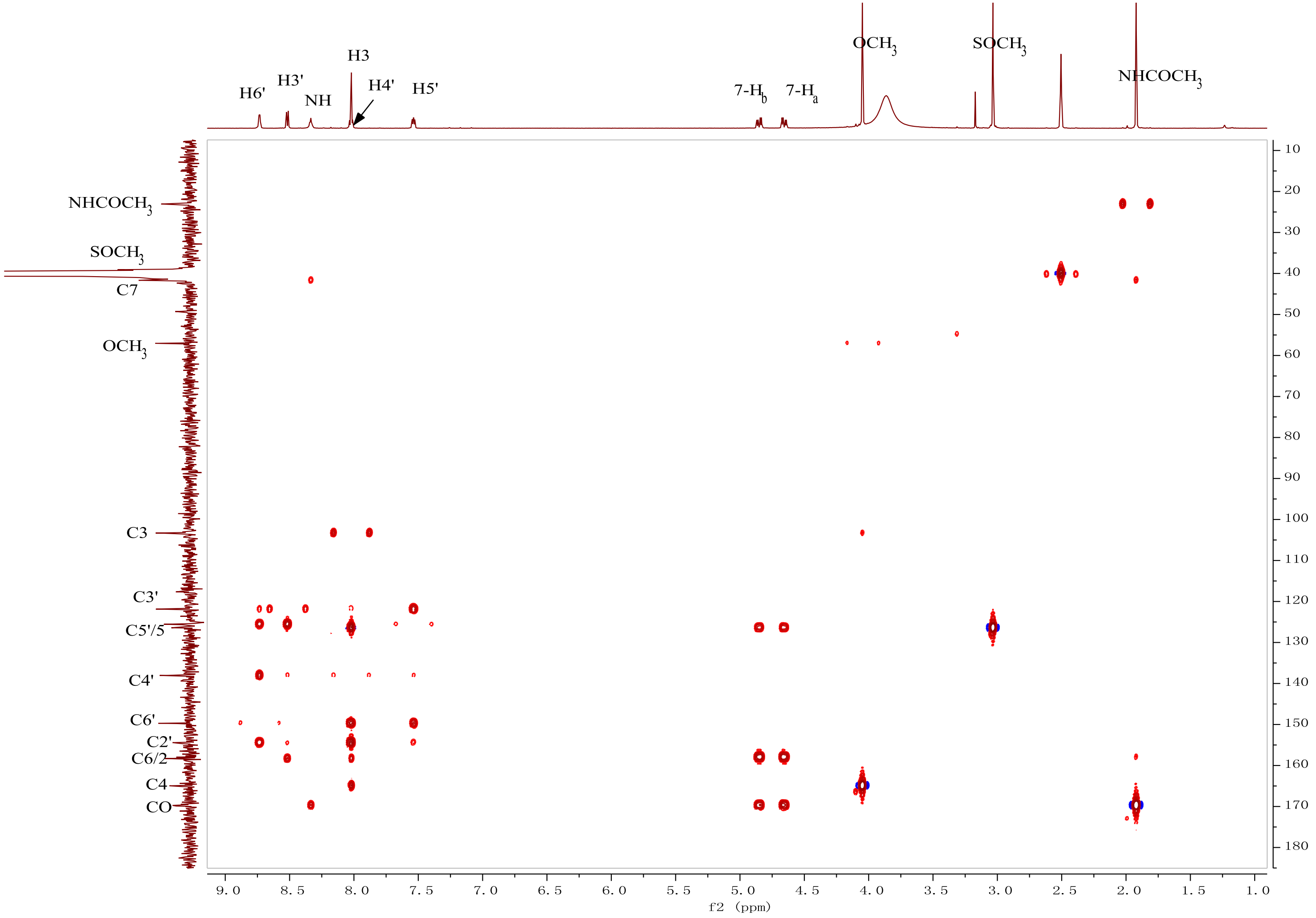

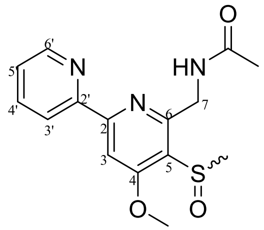

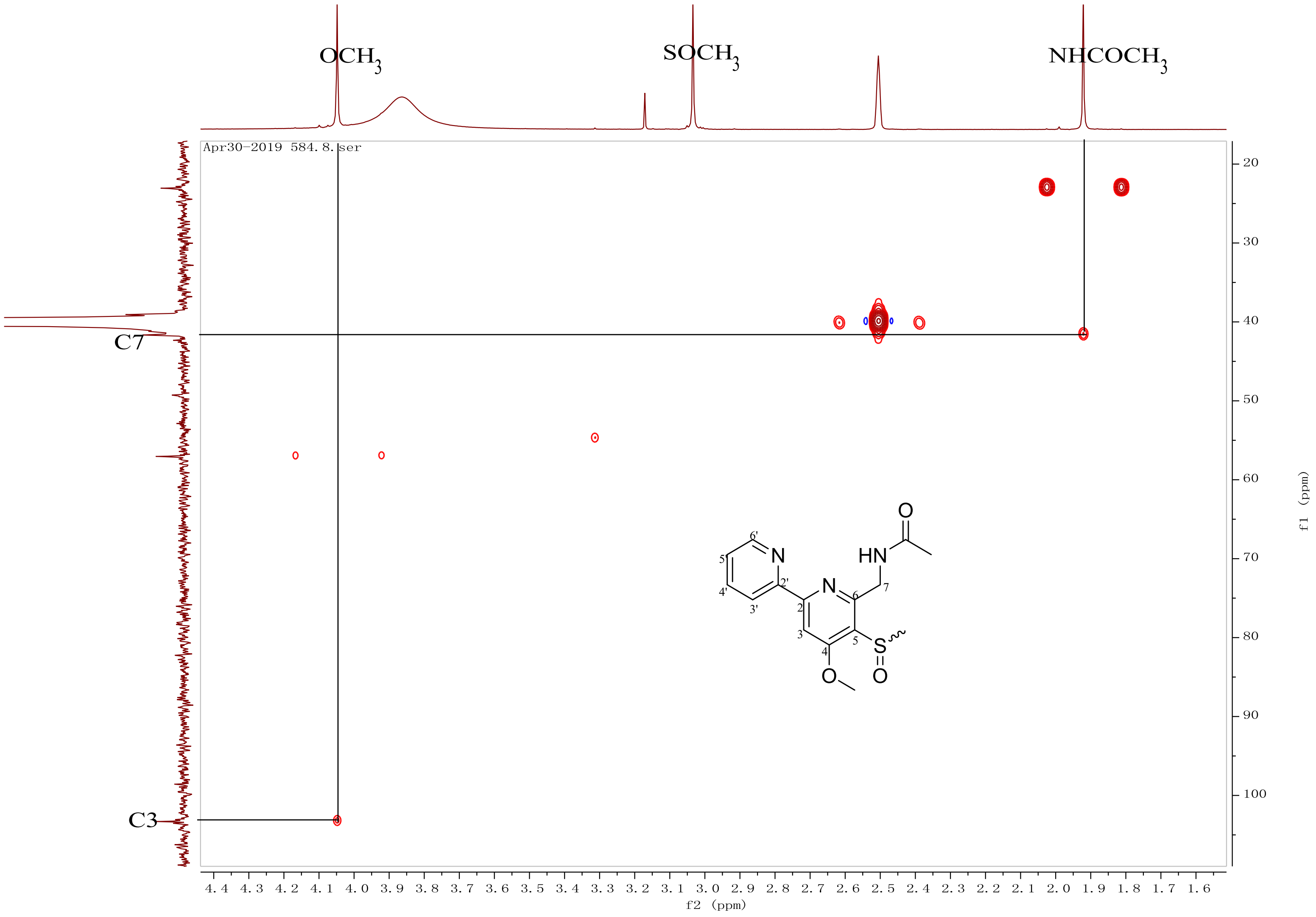

OCH3
SOCH3
NHCOCH3
Apr30-2019 584.8.ser
C7
C3
f2 (ppm)
f1 (ppm)

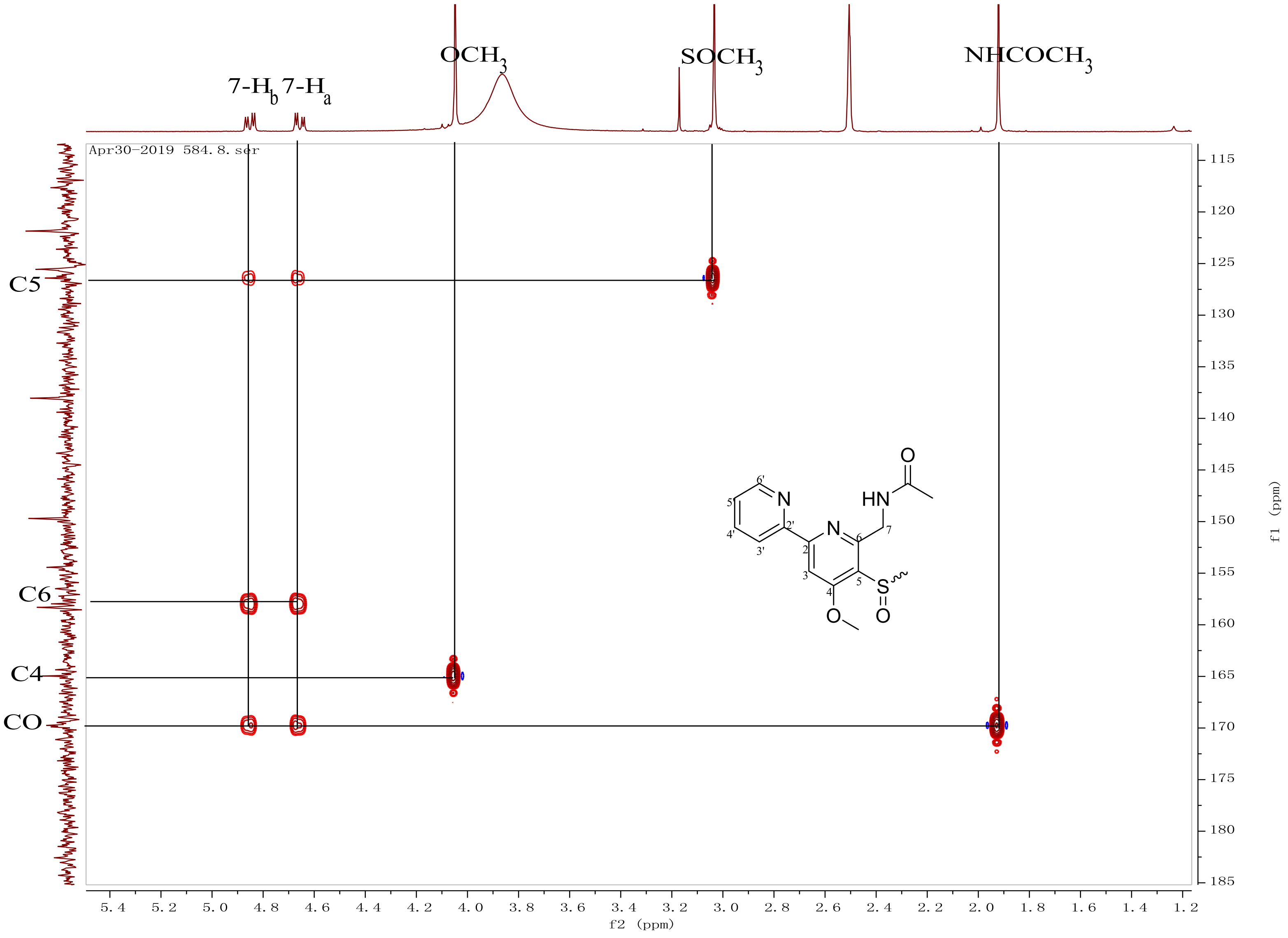
7-Hb 7-Ha
OCH3
SOCH3
NHCOCH3
Apr30-2019 584.8.ser
C5
C6
C4
CO
f2 (ppm)
f1 (ppm)

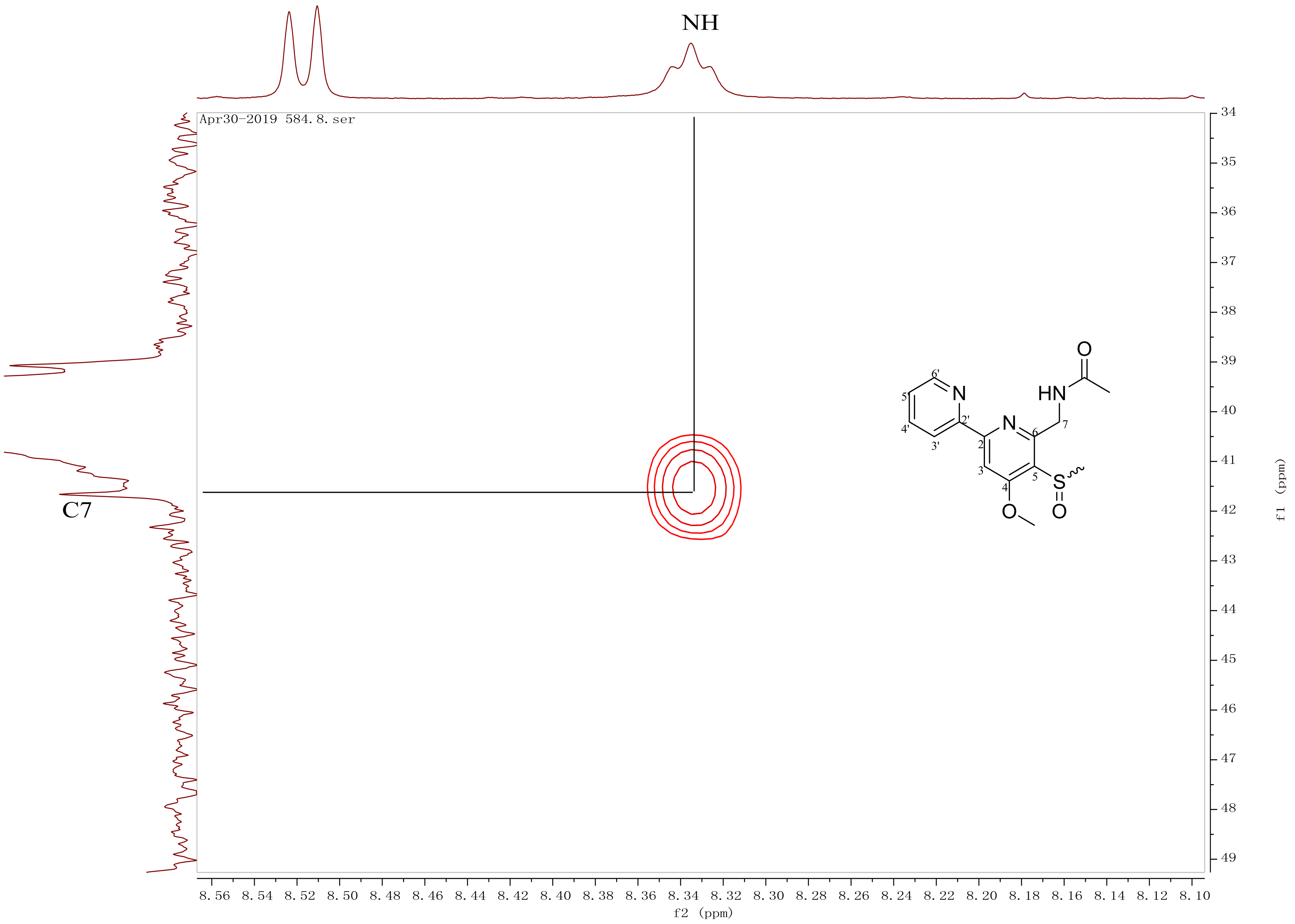
NH
C7
Apr30-2019 584.8.ser
f2 (ppm)
f1 (ppm)
HN
O
N
S

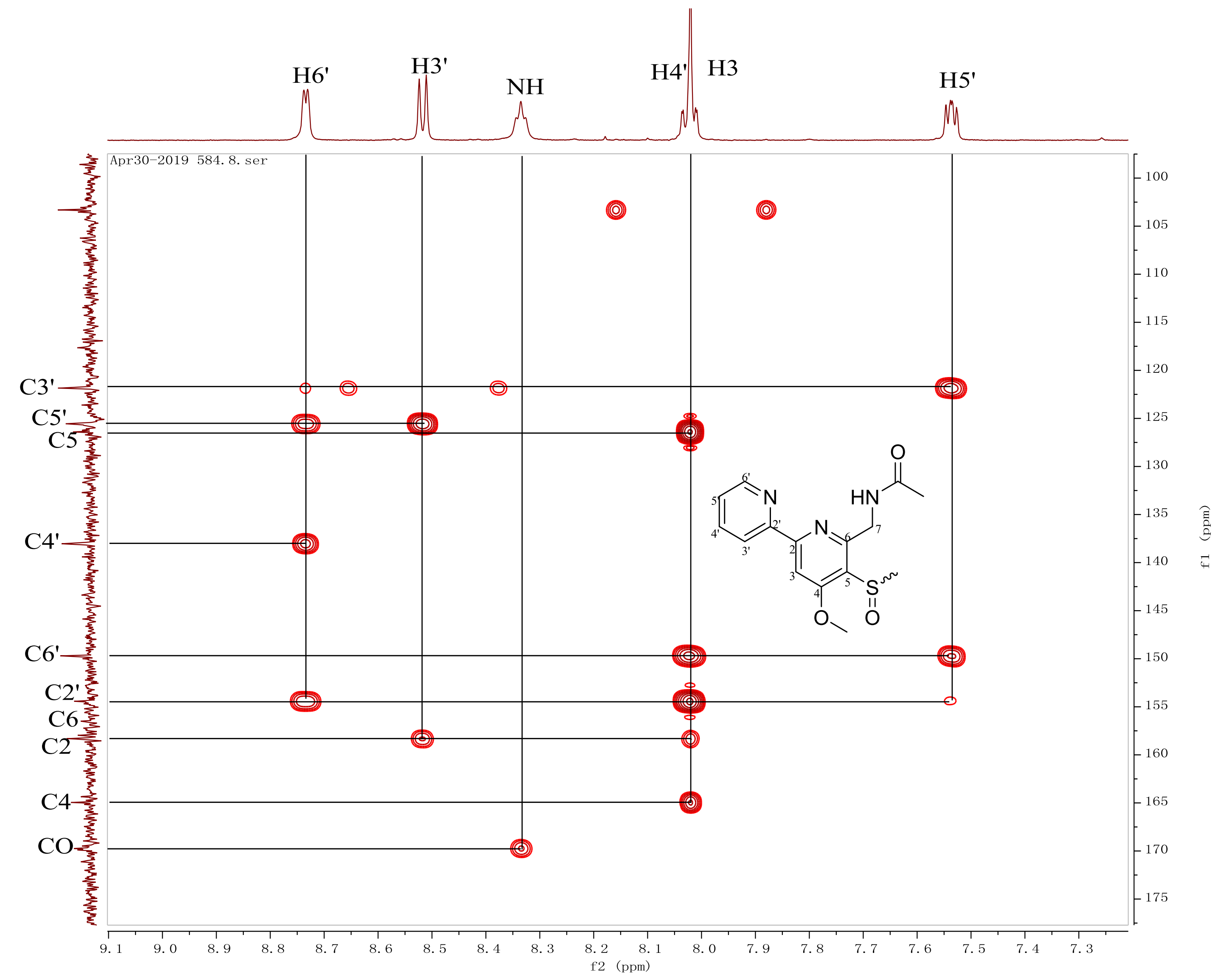
H6'
H3'
NH
H4'
H3
H5'
Apr30-2019 584.8.ser
C3'
C5'
C5
C4'
C6'
C2'
C6
C2
C4
CO
f2 (ppm)
f1 (ppm)

Fig. S31. HRESIMS spectrum of (+)-**3**.

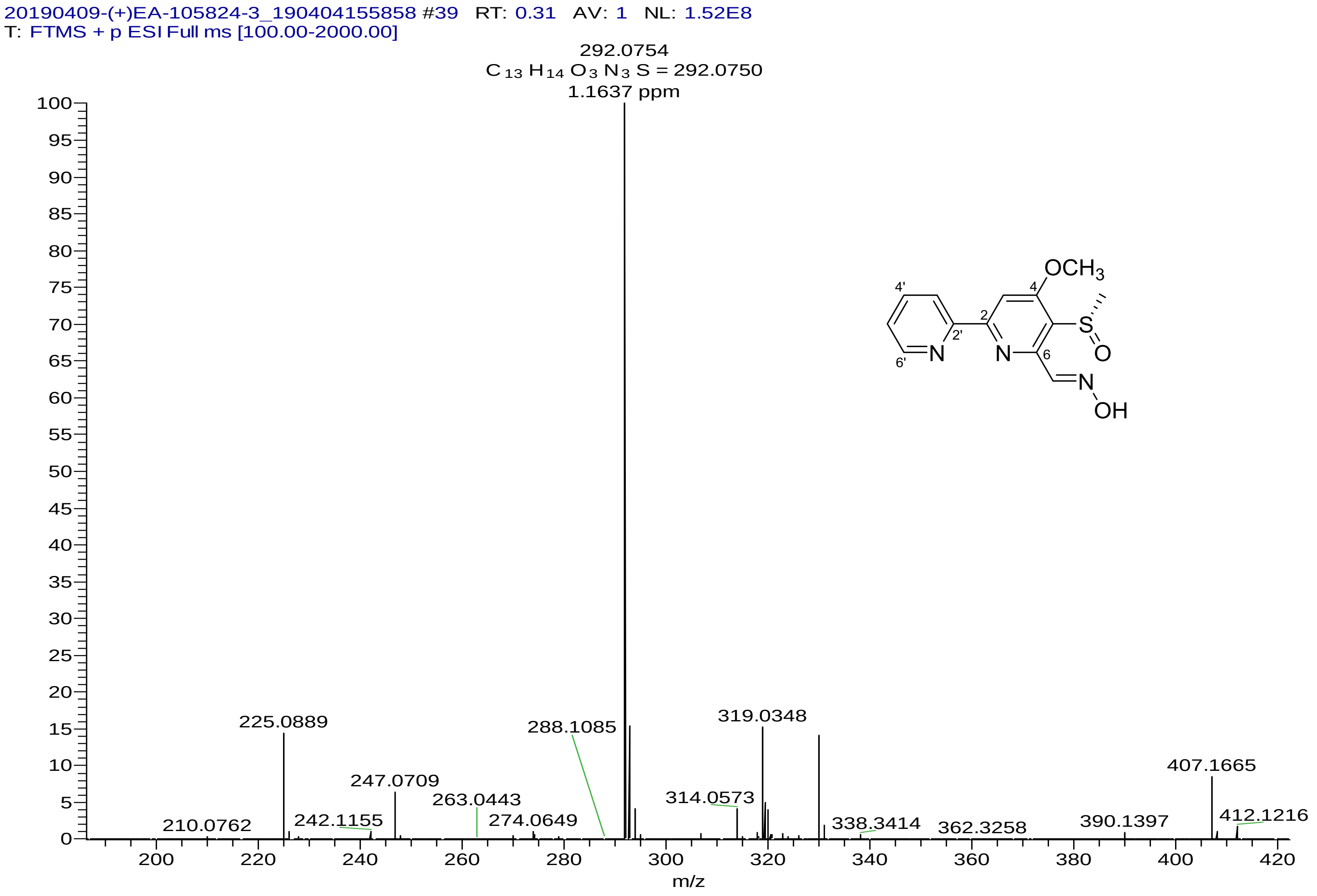

Fig. S32. HRESIMS spectrum of (–)-**3**.

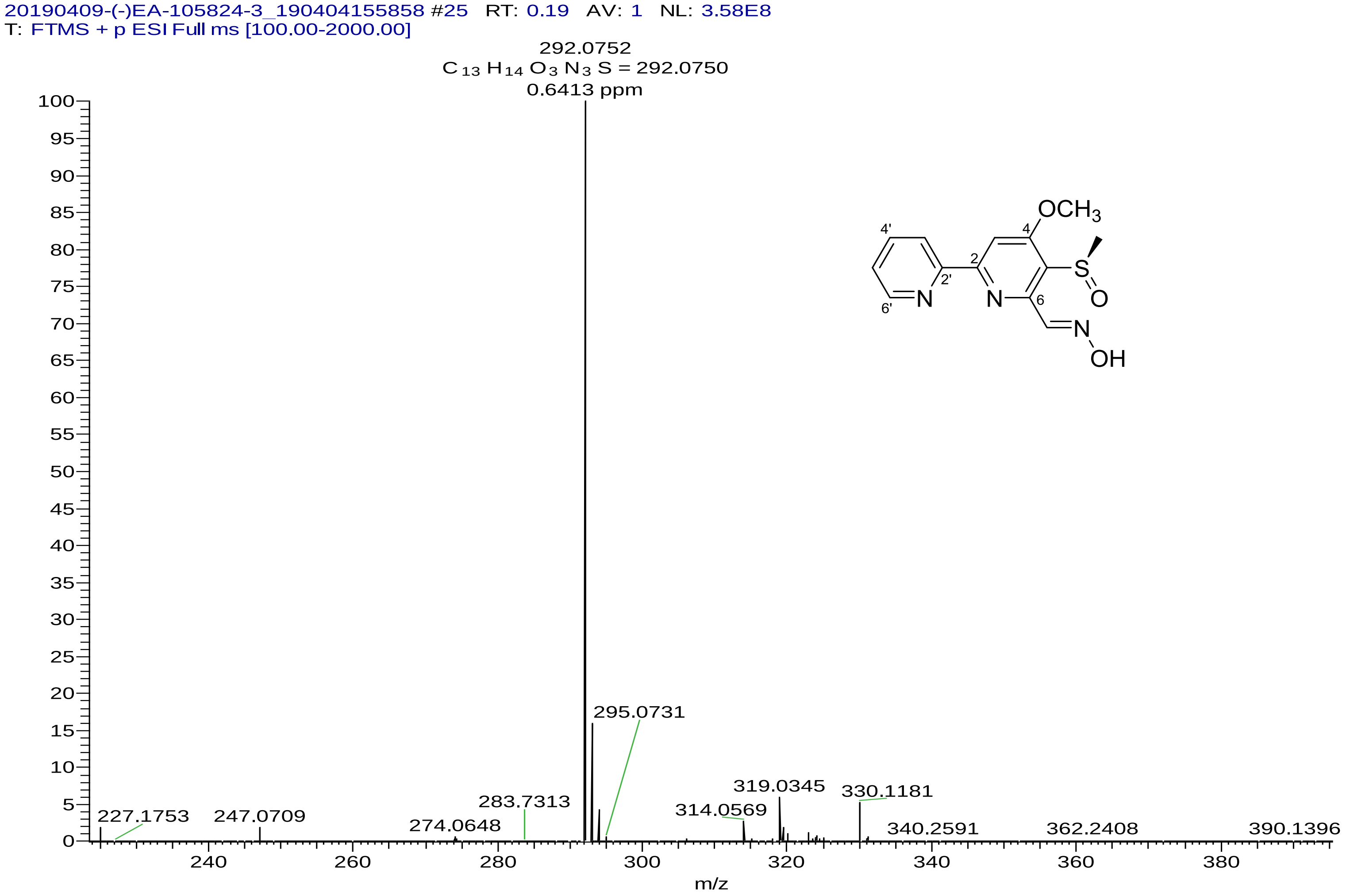

Fig. S33. ^{1}H NMR spectrum of (±)-**3** in CD_3OD.

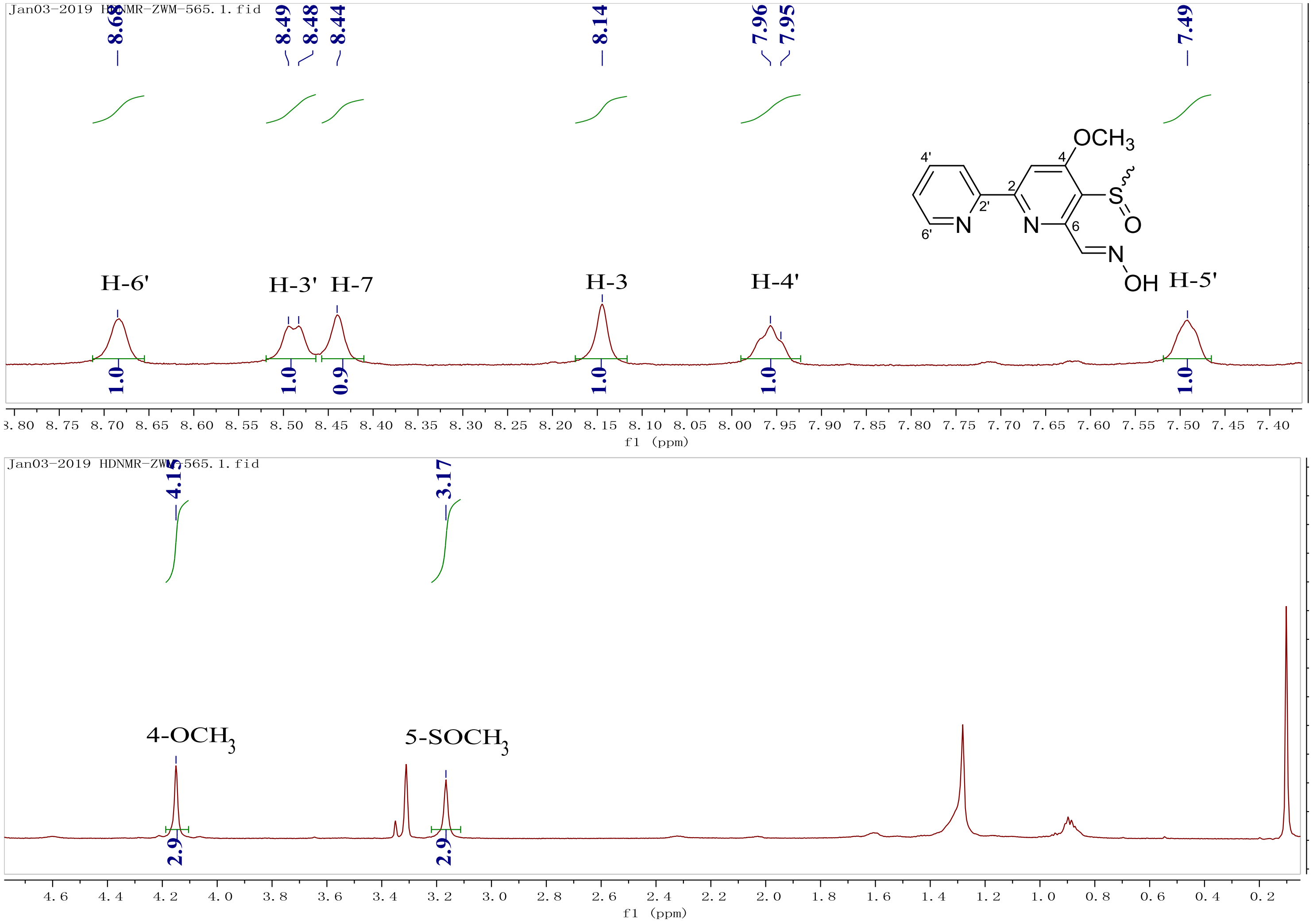

Fig. S34. Q-DEPT NMR spectrum of (±)-**3** in CD_3OD.

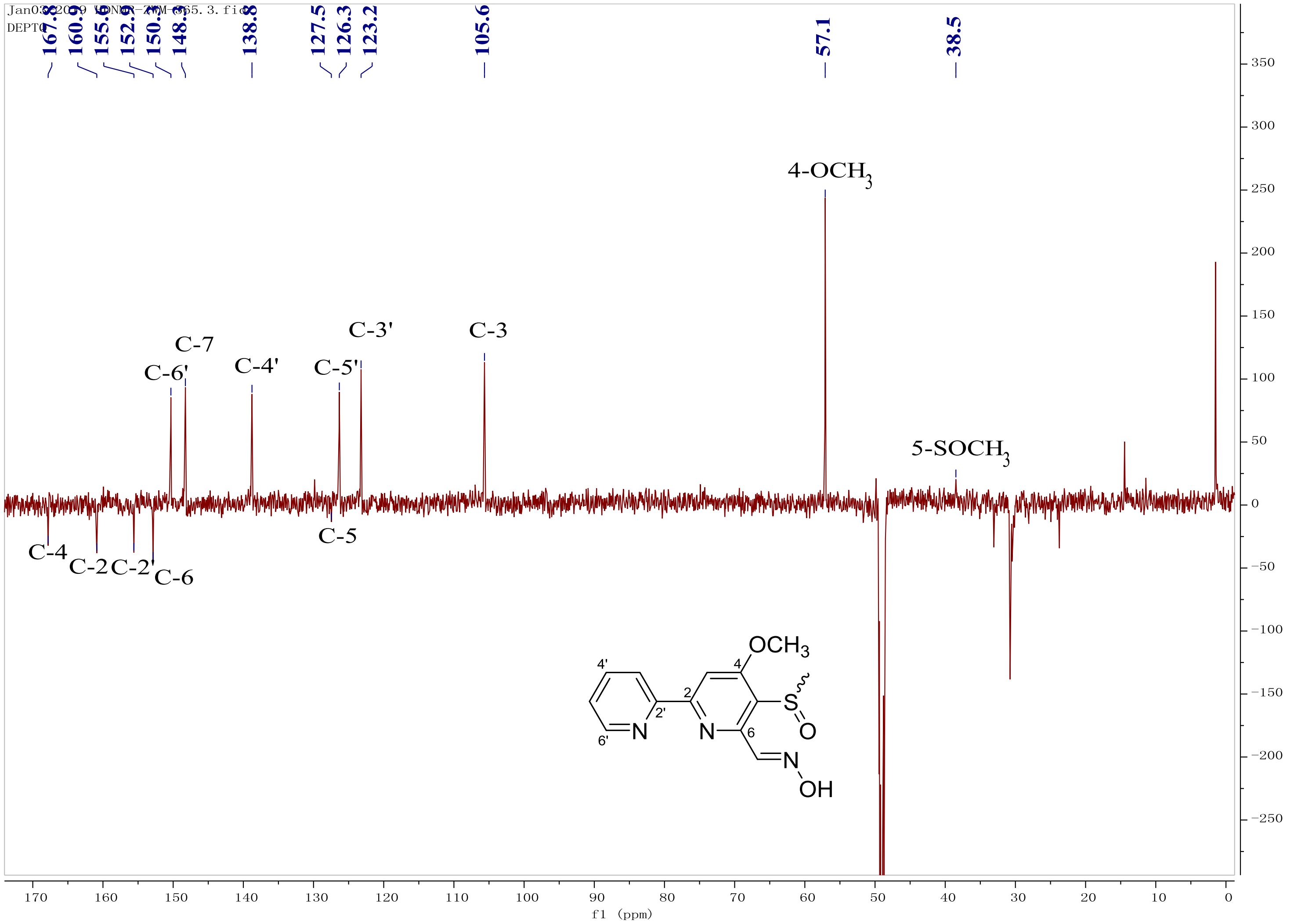

Fig. S35. HRESIMS spectrum of (+)-**4**.

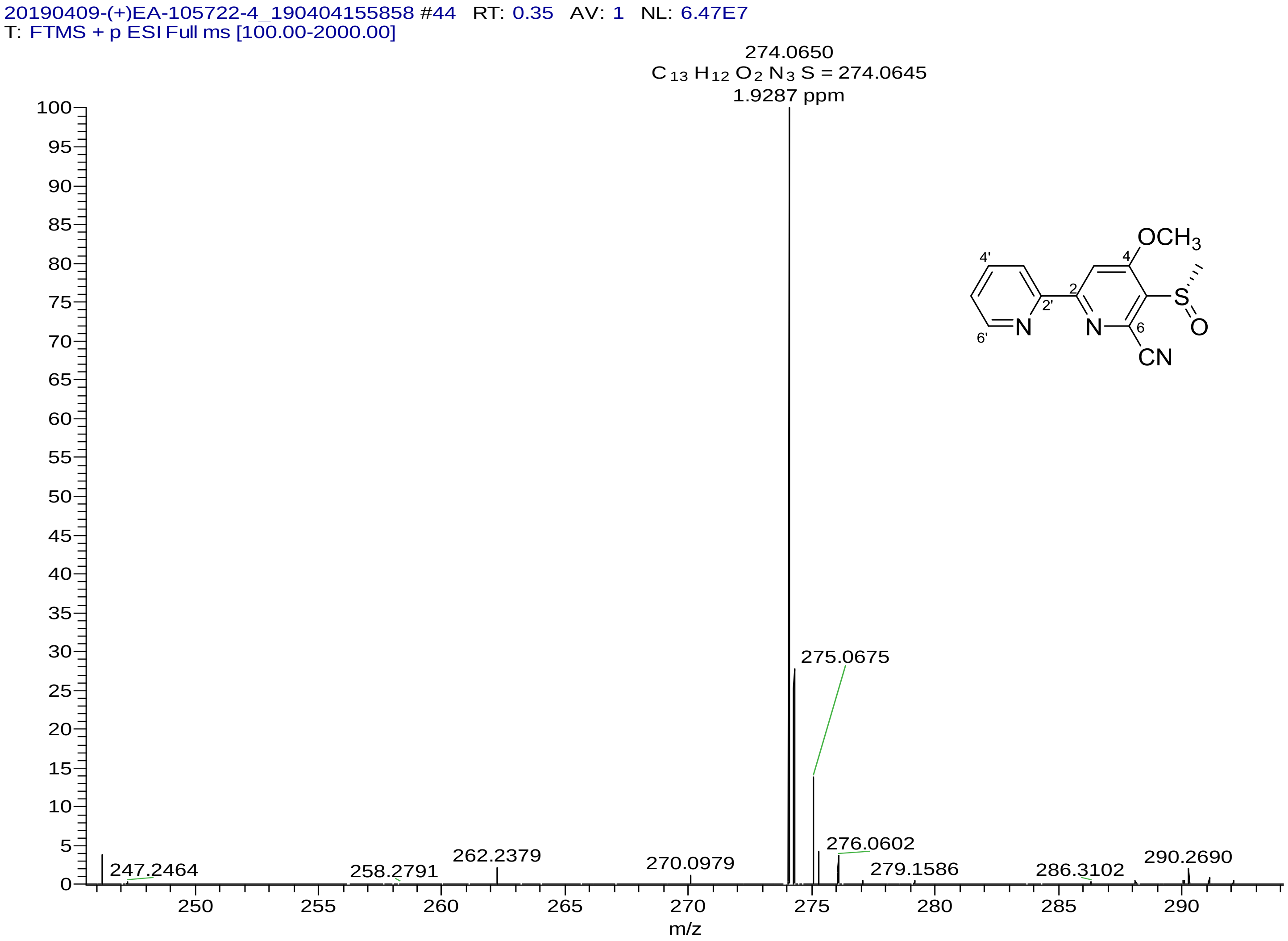

Fig. S36. HRESIMS spectrum of (–)-**4**.

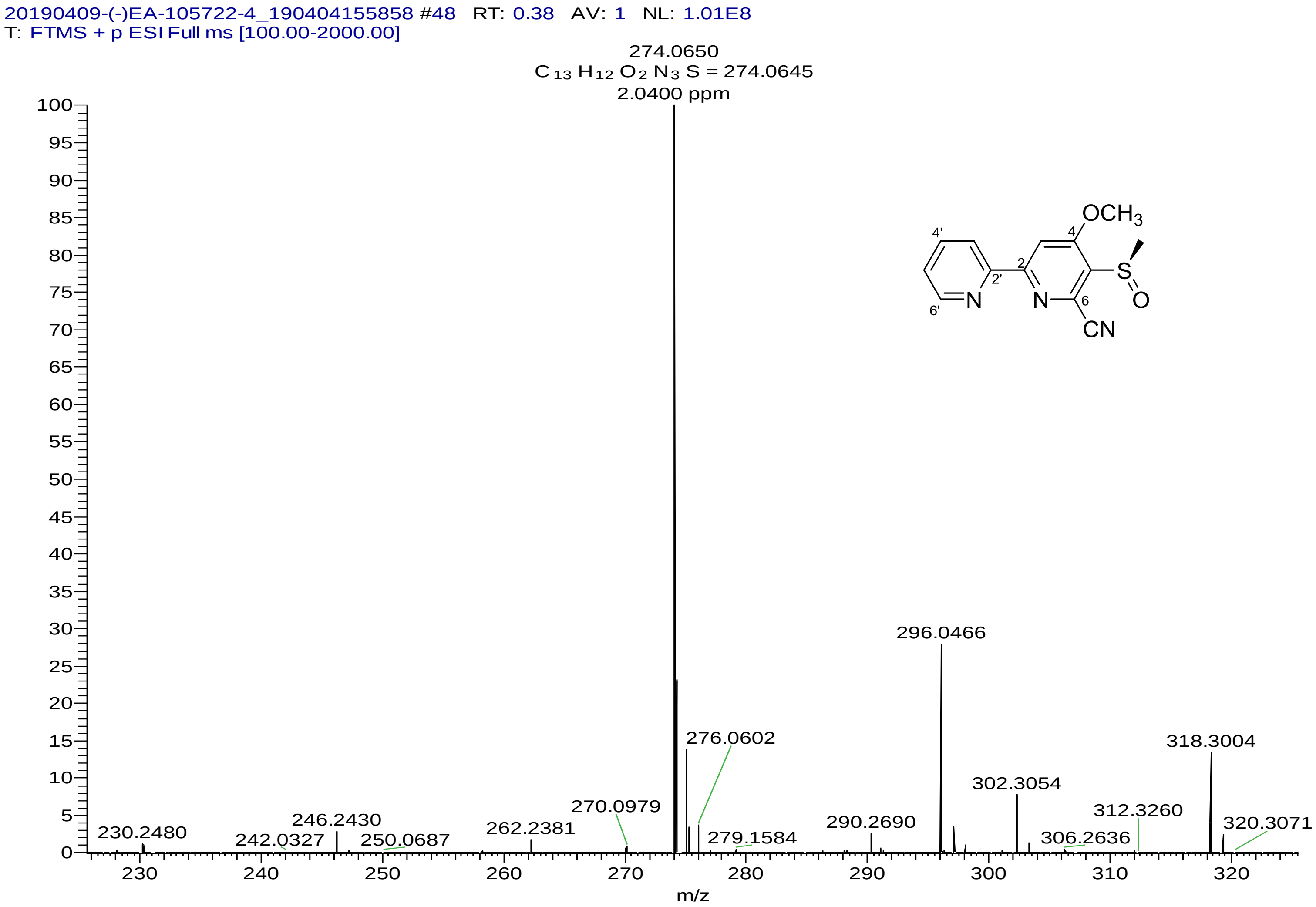

Fig. S37. ^{1}H NMR spectrum of (±)-**4** in $CDCl_3$.

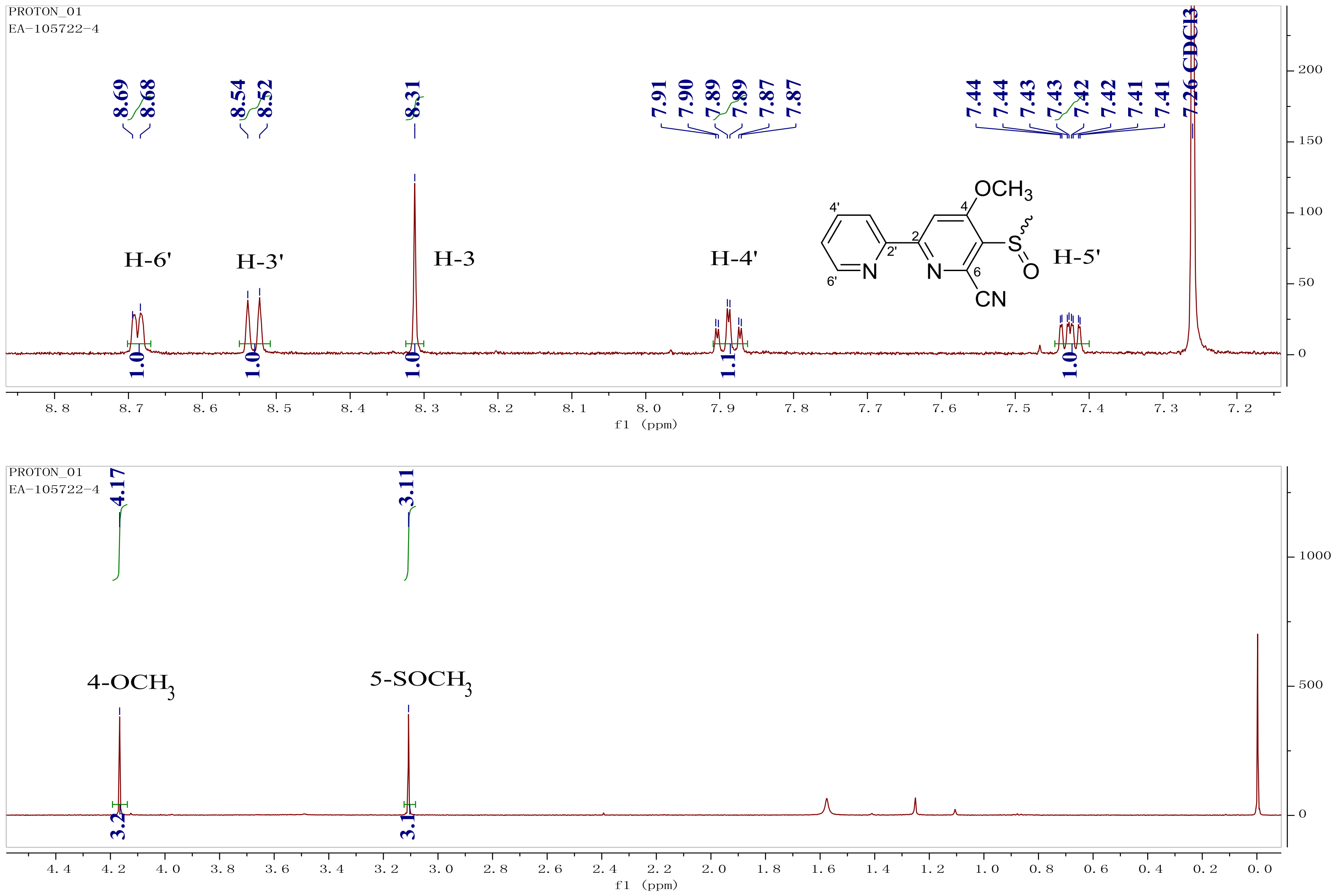

Fig. S38. ^{13}C NMR spectrum of (±)-**4** in $CDCl_3$.

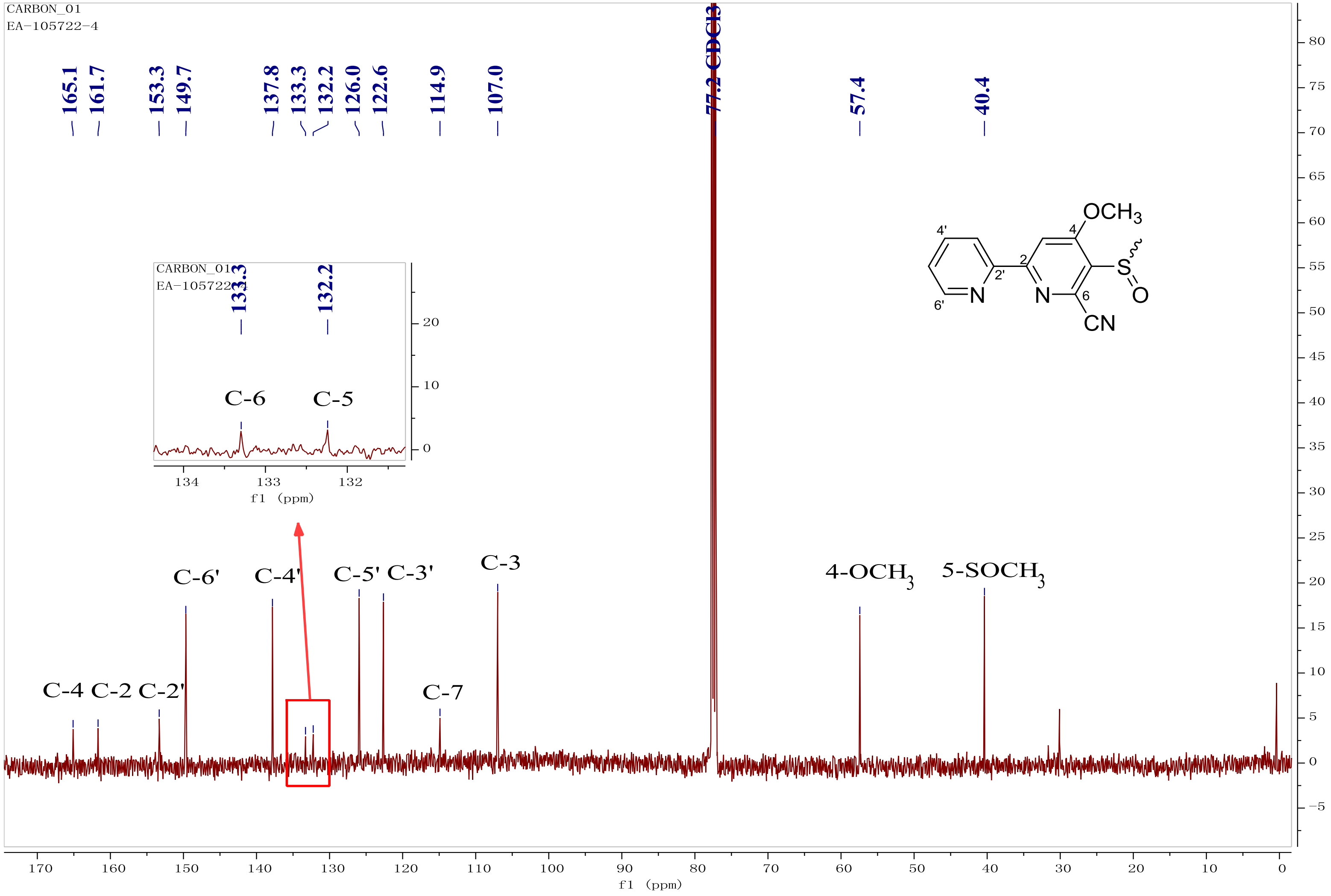

Fig. S39. Co-HPLC profiles of the synthetic and the natural (±)-**2**.

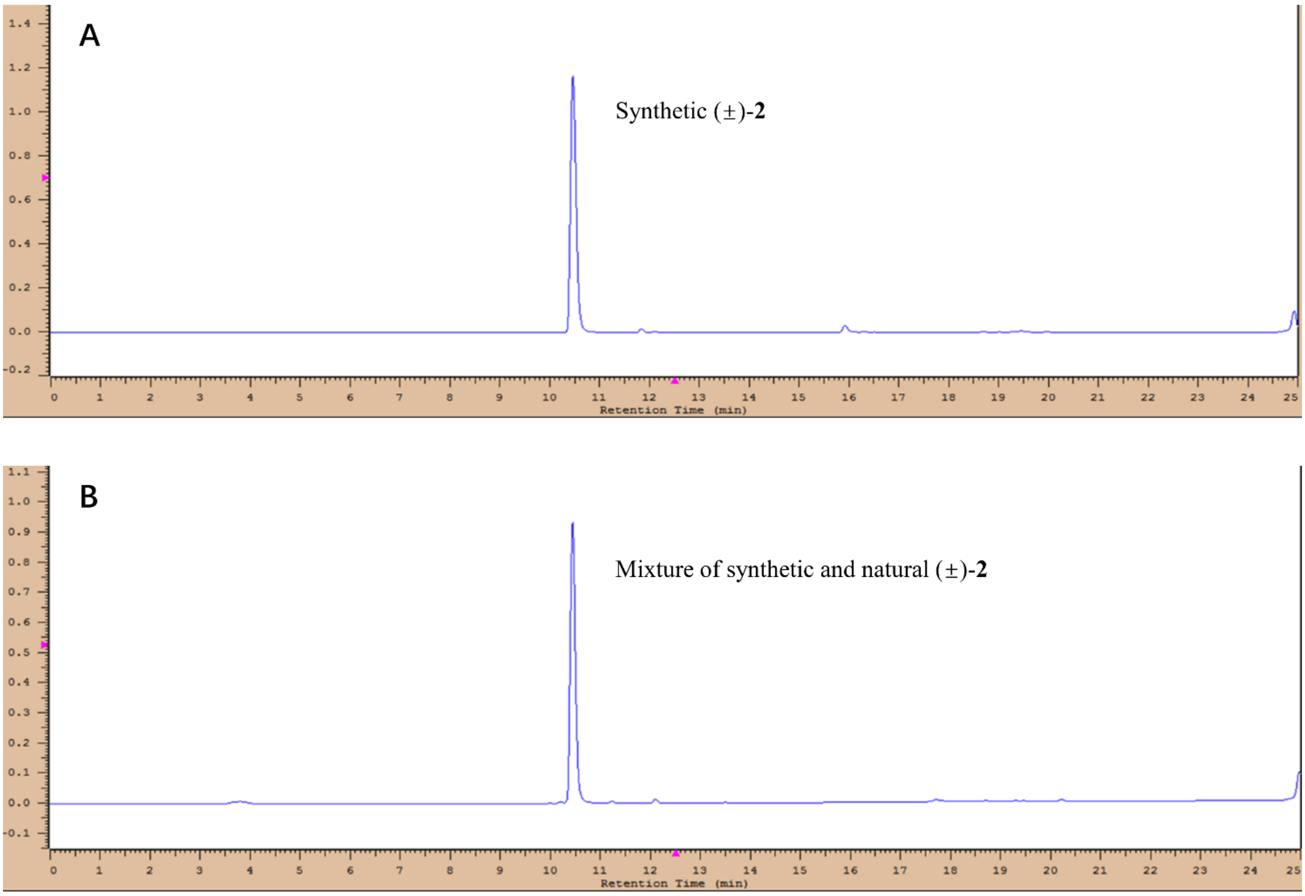

Co-HPLC profiles of the synthetic and natural (±)-**2** on an YMC ODS-C18 column (flow rate: 1 mL/min; solvent: MeOH/H_2O gradient from 0–100%; detection: 275 nm; temperature: 30 °C). (**A**) HPLC profile of the synthetic (±)-**2**; (**B**) mixed HPLC profile of the synthetic and natural (±)-**2**.

Fig. S40. HRESIMS spectrum of **13**.

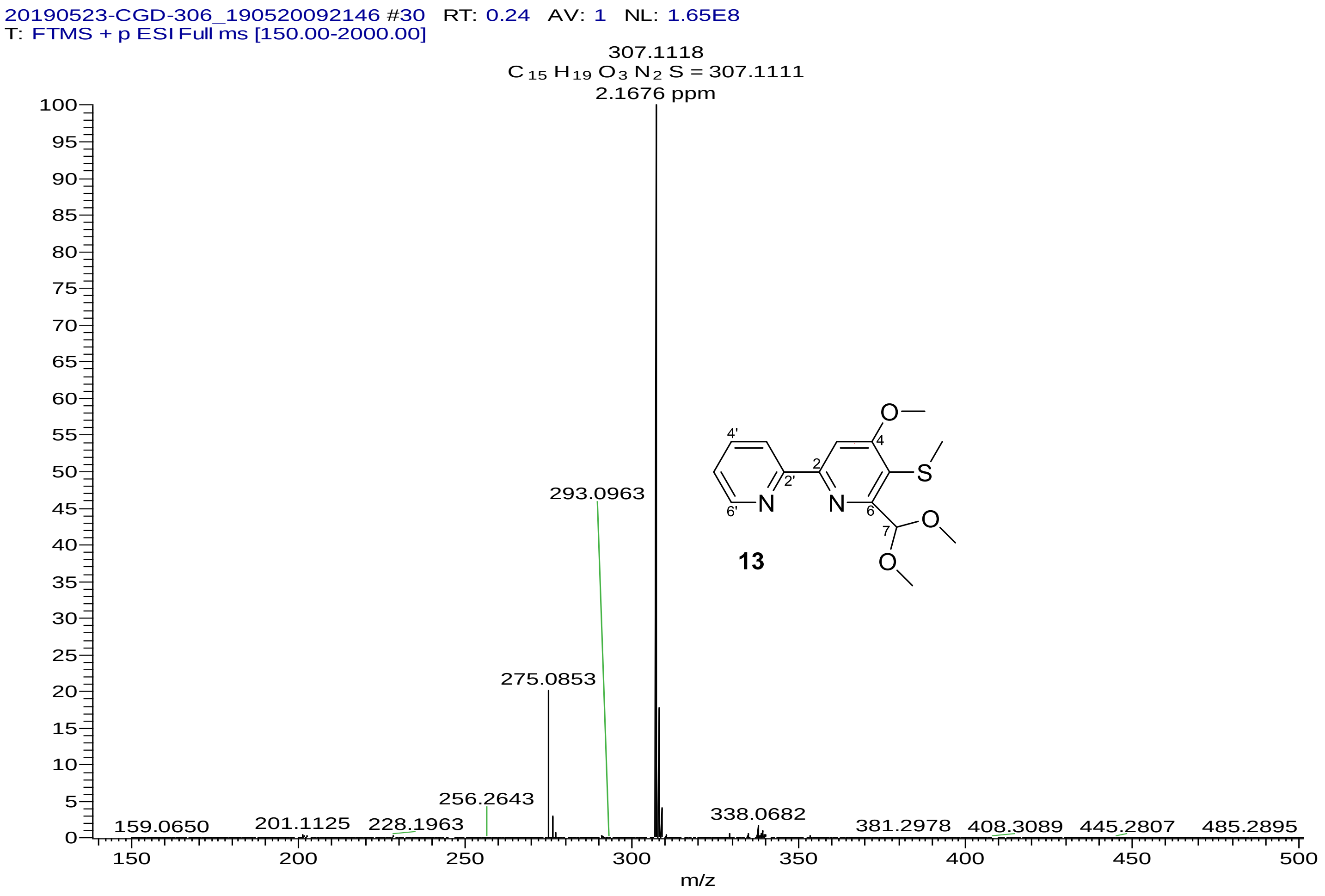

Fig. S41. ^{1}H NMR spectrum of **13** in $CDCl_3$.

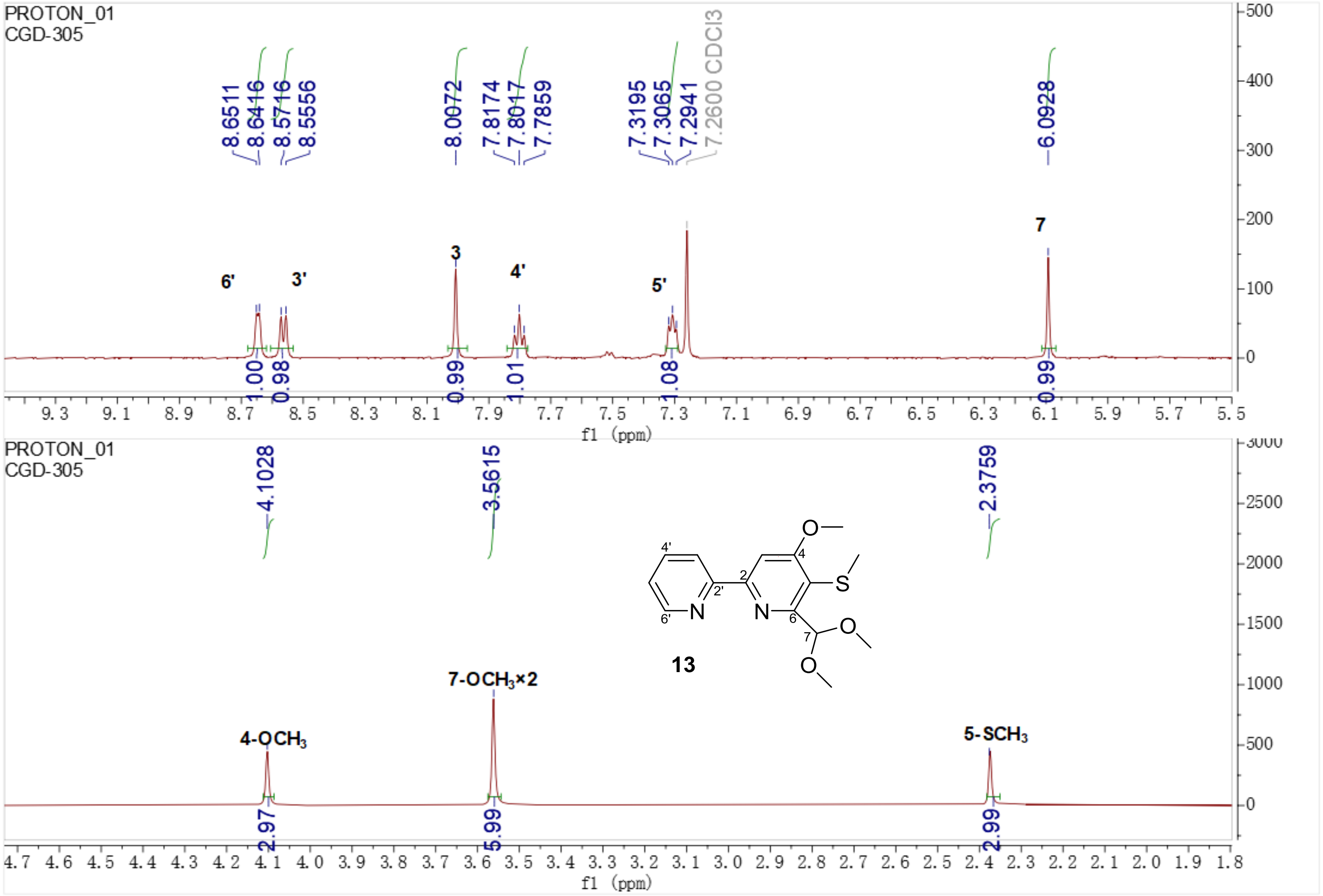

Fig. S42. ^{13}C NMR spectrum of **13** in $CDCl_3$.

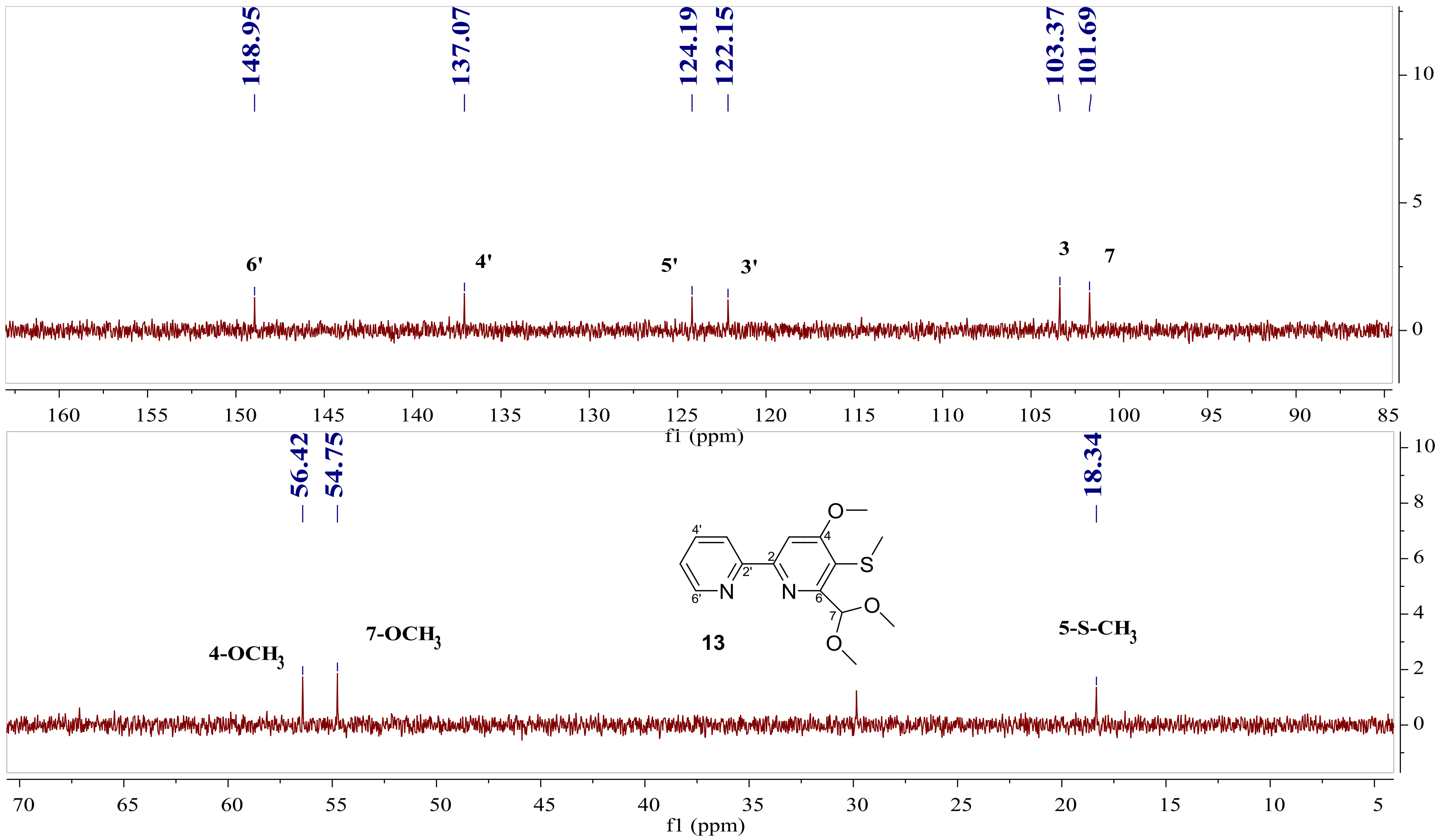

Fig. S43. HSQC spectrum of **13** in $CDCl_3$.

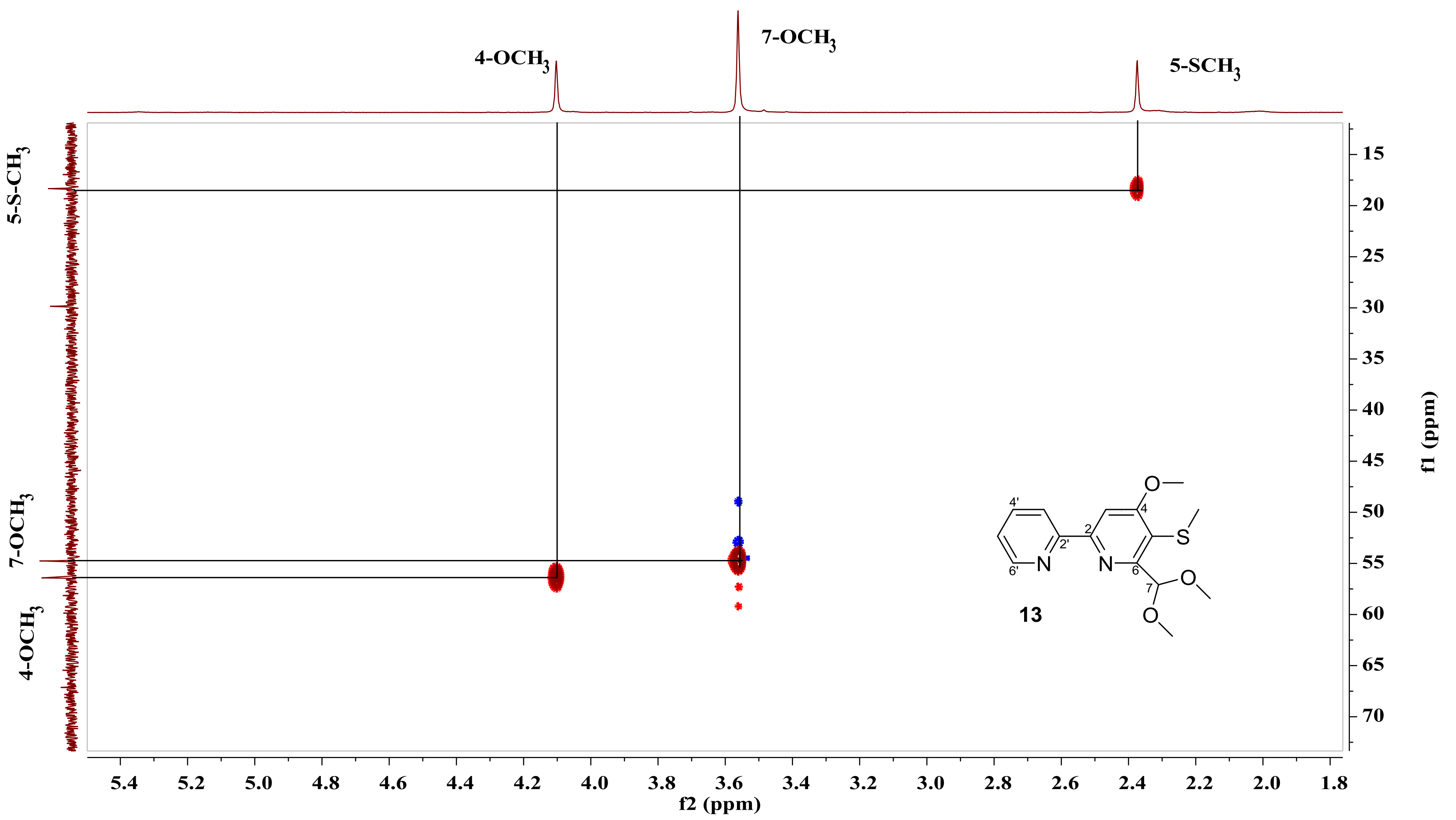

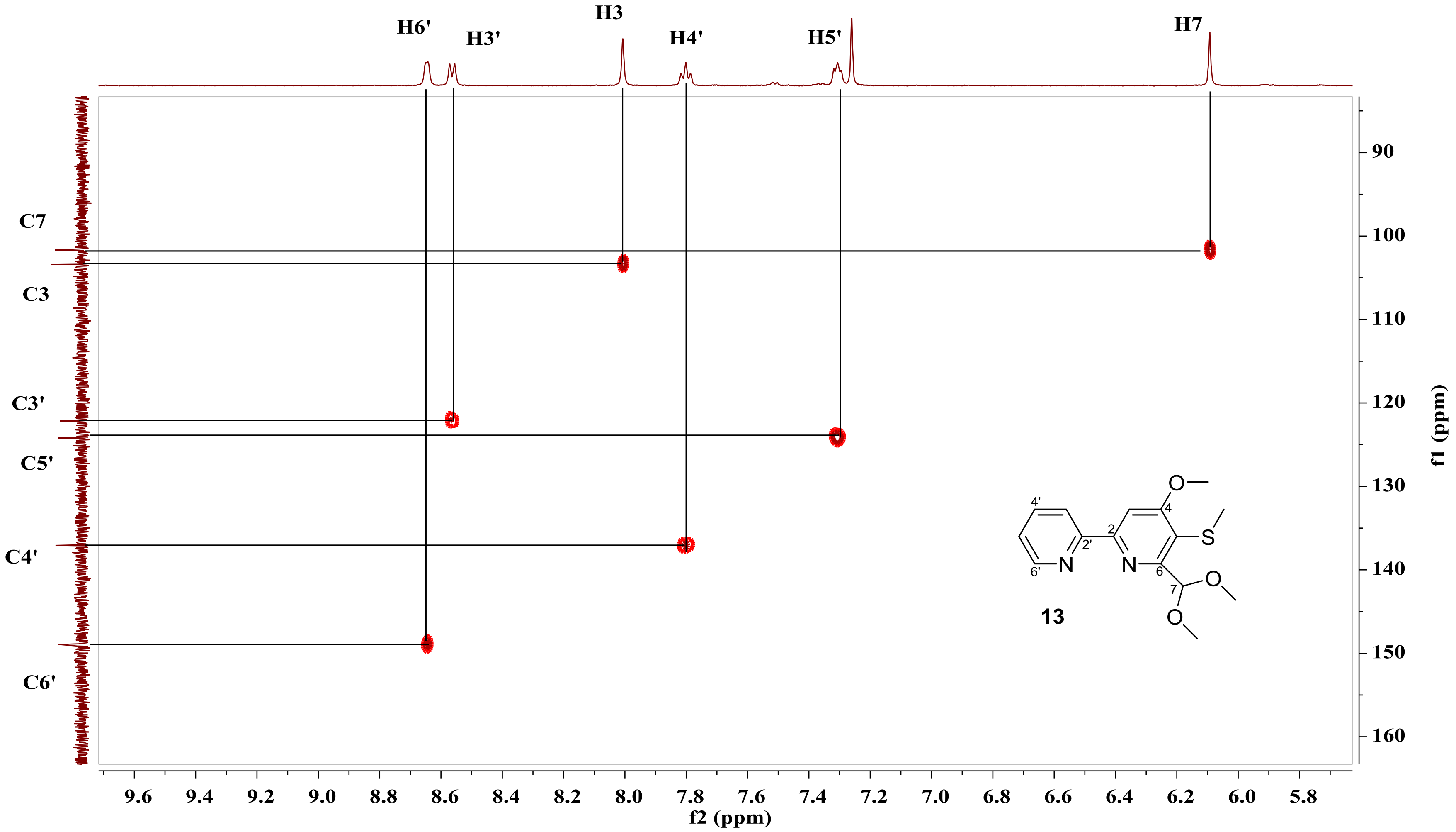
H6'
H3'
H3
H4'
H5'
H7
C7
C3
C3'
C5'
C4'
C6'
90
100
110
120
130
140
150
160
f1 (ppm)
9.6
9.4
9.2
9.0
8.8
8.6
8.4
8.2
8.0
7.8
7.6
7.4
7.2
7.0
6.8
6.6
6.4
6.2
6.0
5.8
f2 (ppm)
13

Fig. S44. ^{1}H-^{1}H COSY spectrum of **13** in $CDCl_3$.

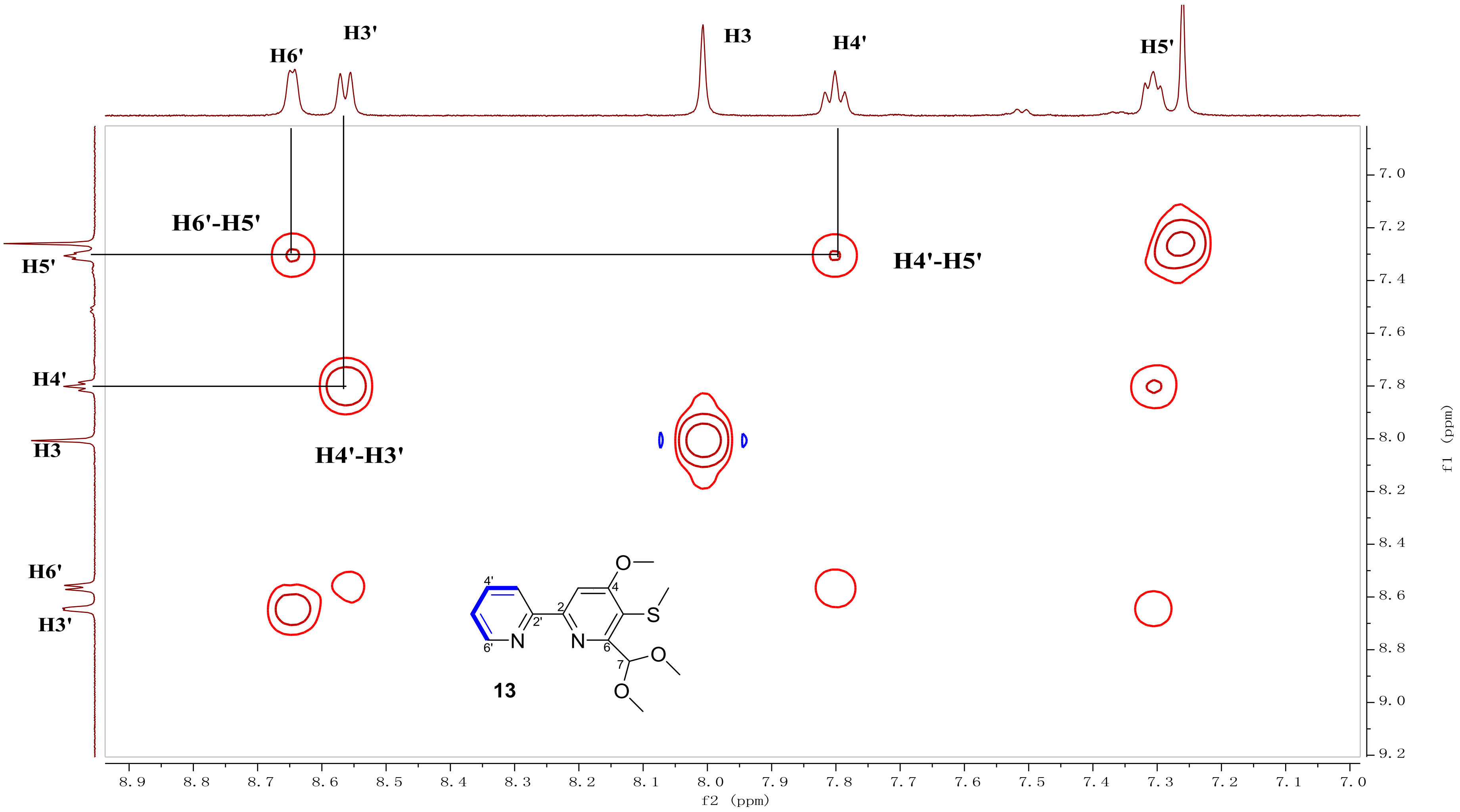

Fig. S45. HMBC spectrum of **13** in CDCl3.

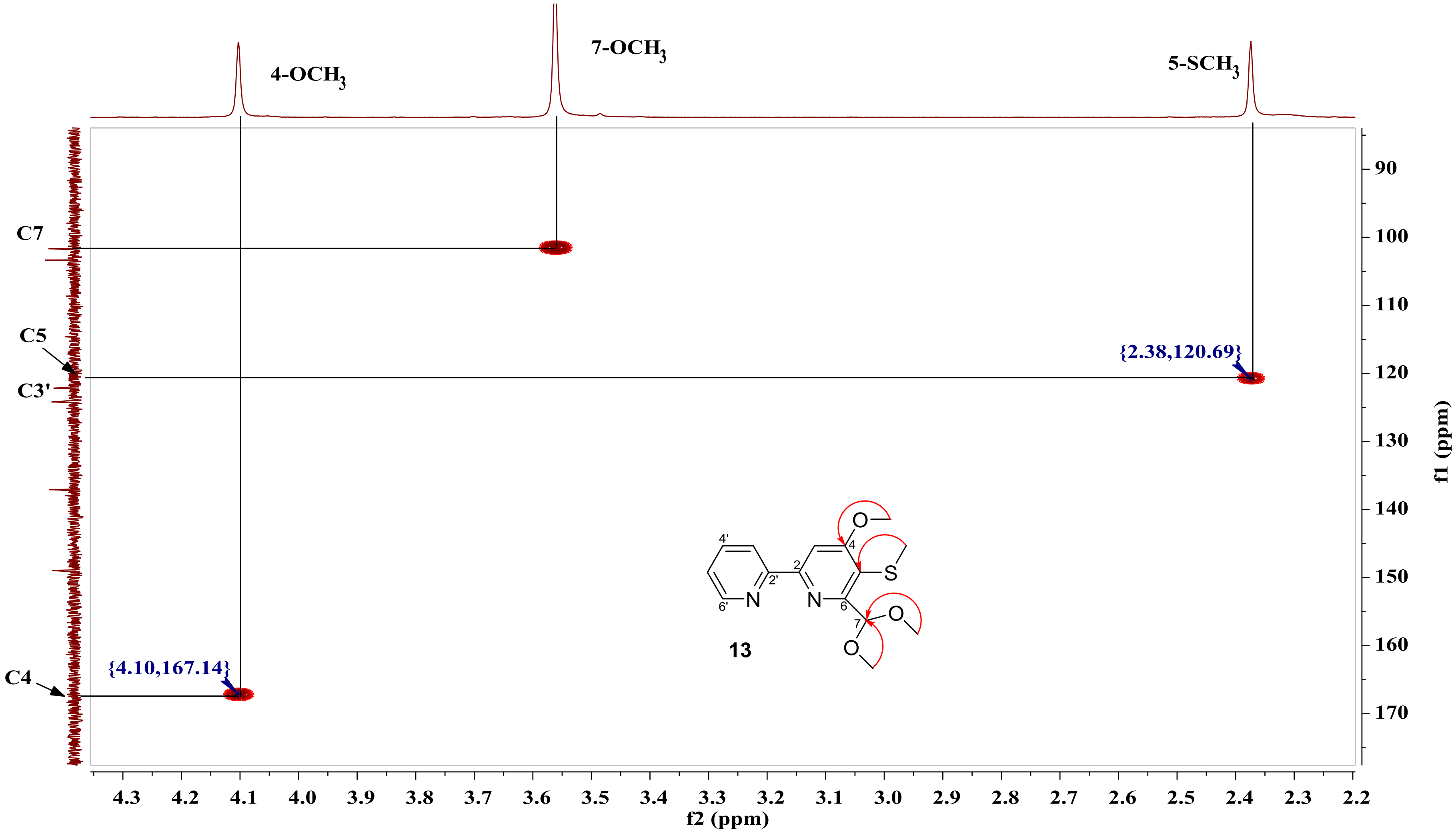

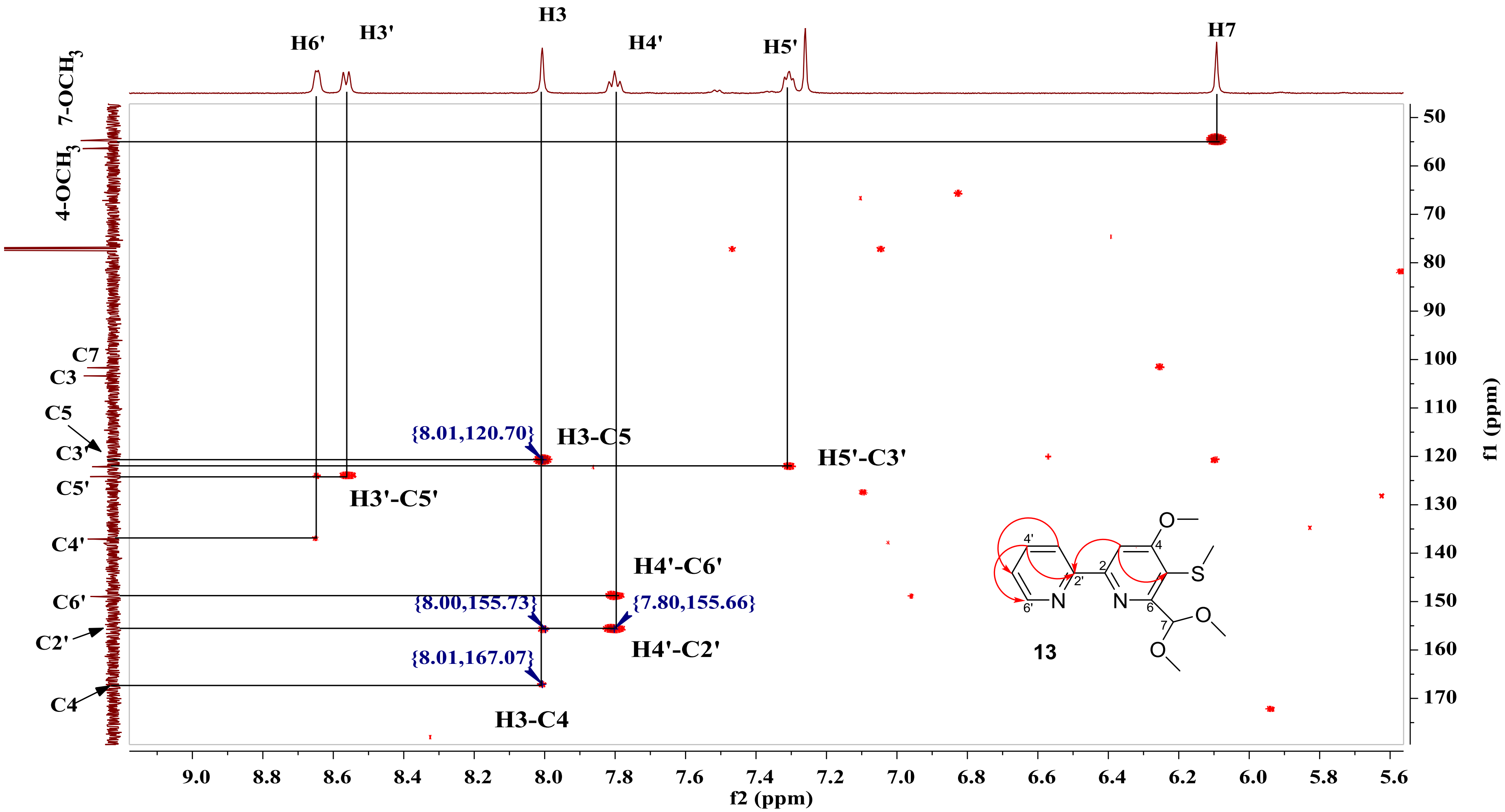

H6'
H3'
H3
H4'
H5'
H7
7-OCH3
4-OCH3
C7
C3
C5
C3'
C5'
C4'
C6'
C2'
C4
{8.01,120.70}
H3-C5
H5'-C3'
H3'-C5'
H4'-C6'
{8.00,155.73}
{7.80,155.66}
H4'-C2'
{8.01,167.07}
H3-C4
13
f2 (ppm)
f1 (ppm)

Fig. S46. HRESIMS spectrum of (±)-**14**.

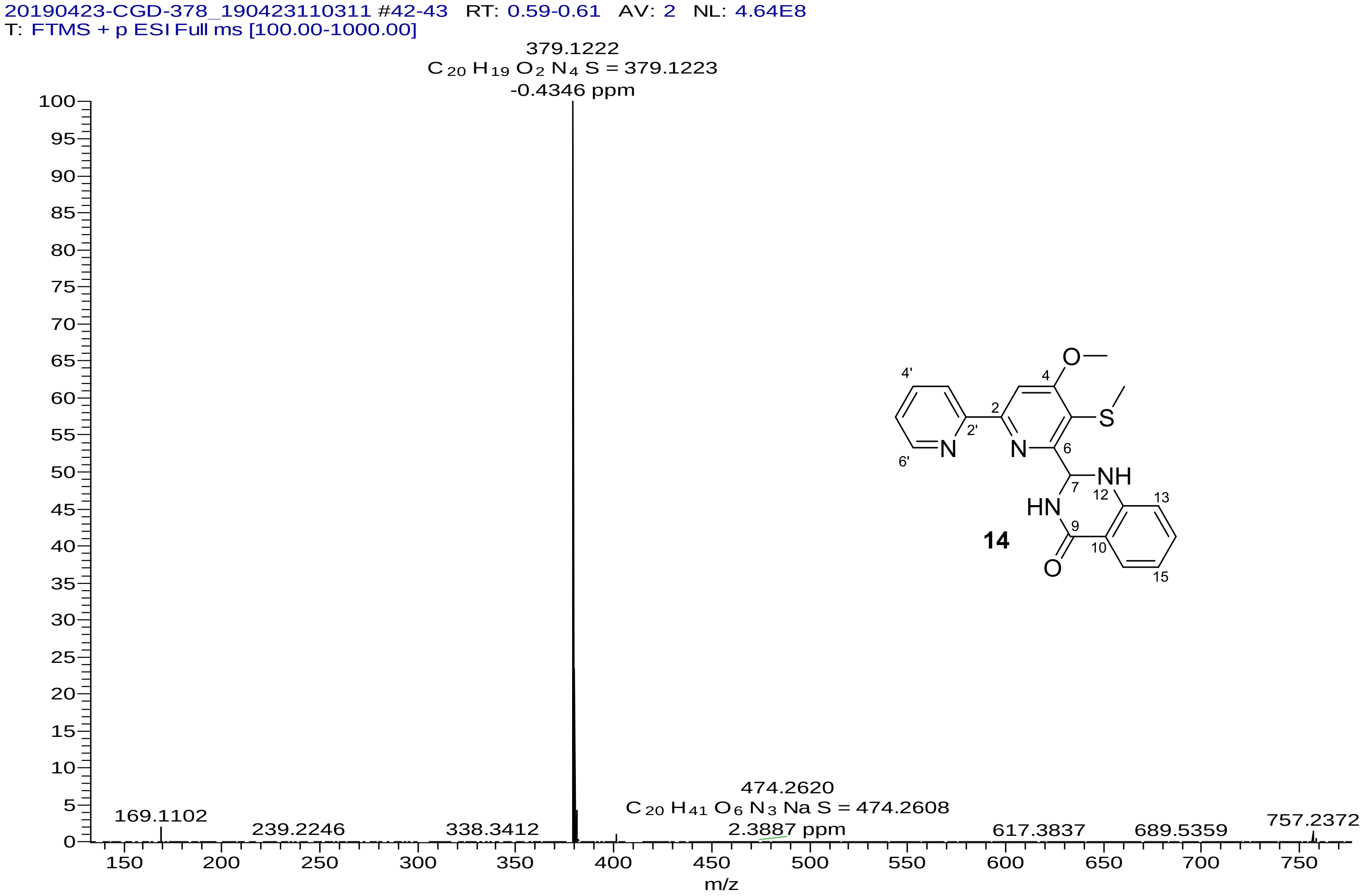

14

Fig. S47. ^{1}H NMR spectrum of (±)-**14** in $CDCl_3$.

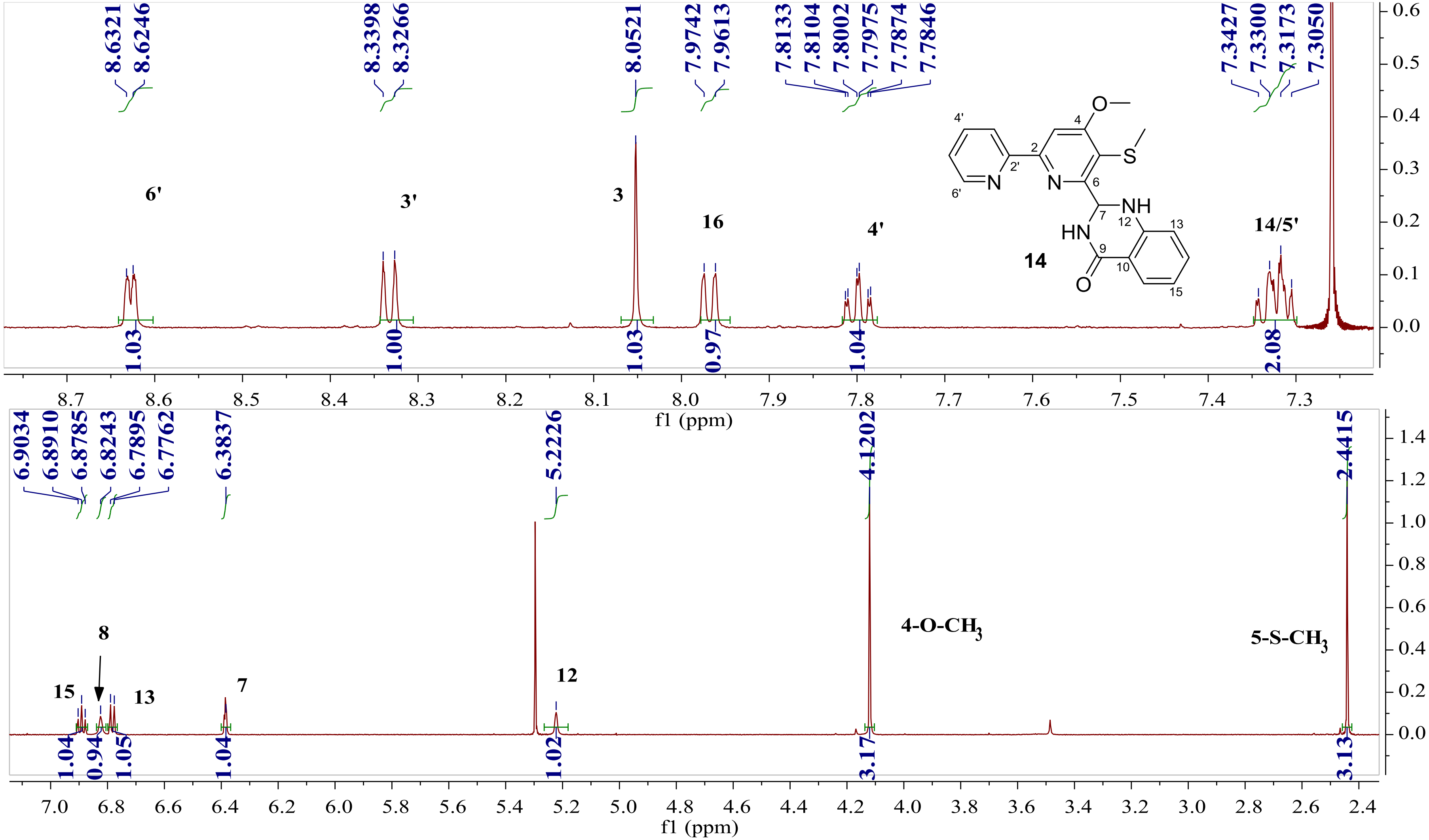

Fig. S48. ^{13}C NMR spectrum of (±)-**14** in $CDCl_3$.

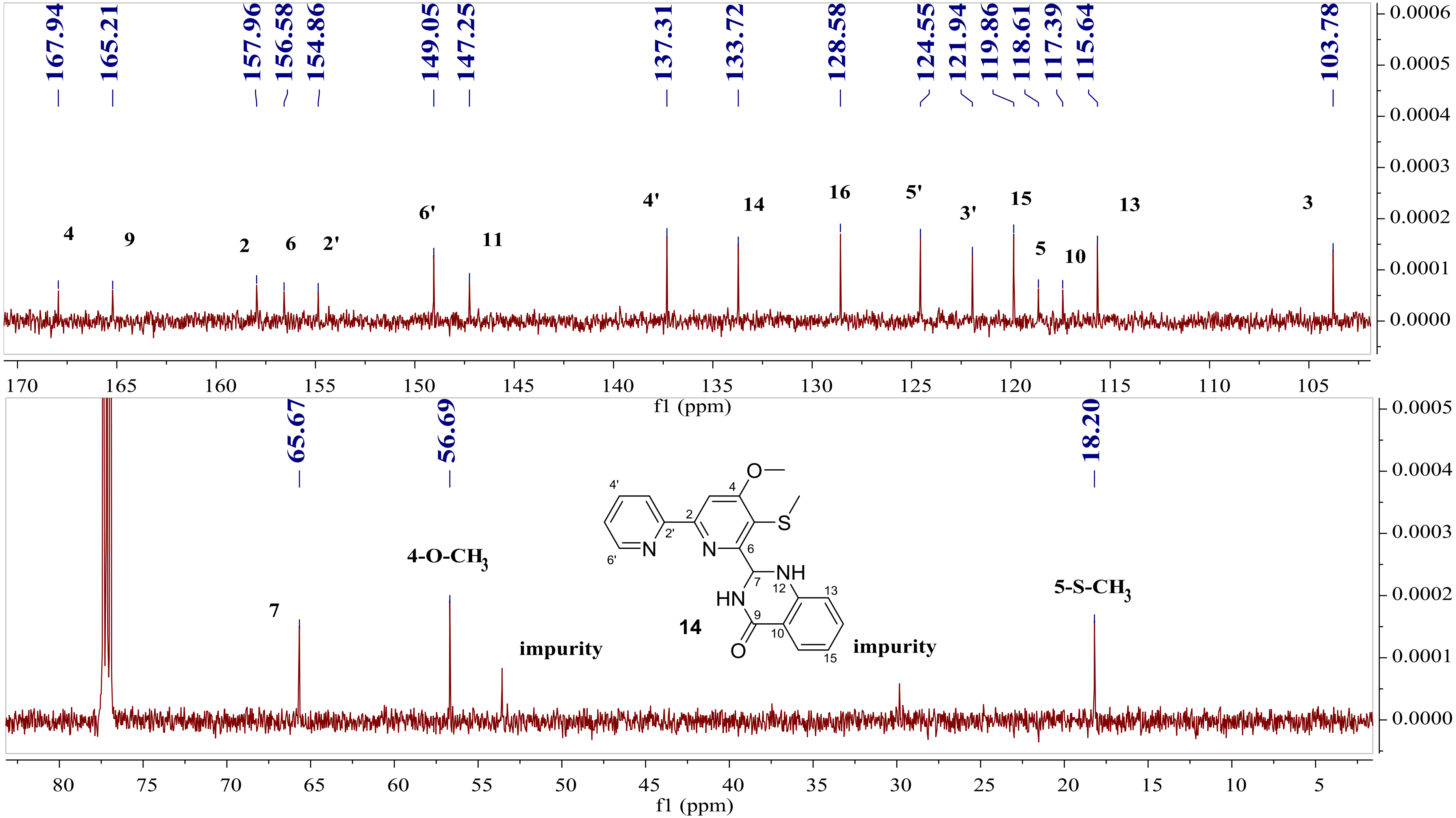

Fig. S49. HSQC spectrum of (±)-**14** in $CDCl_3$.

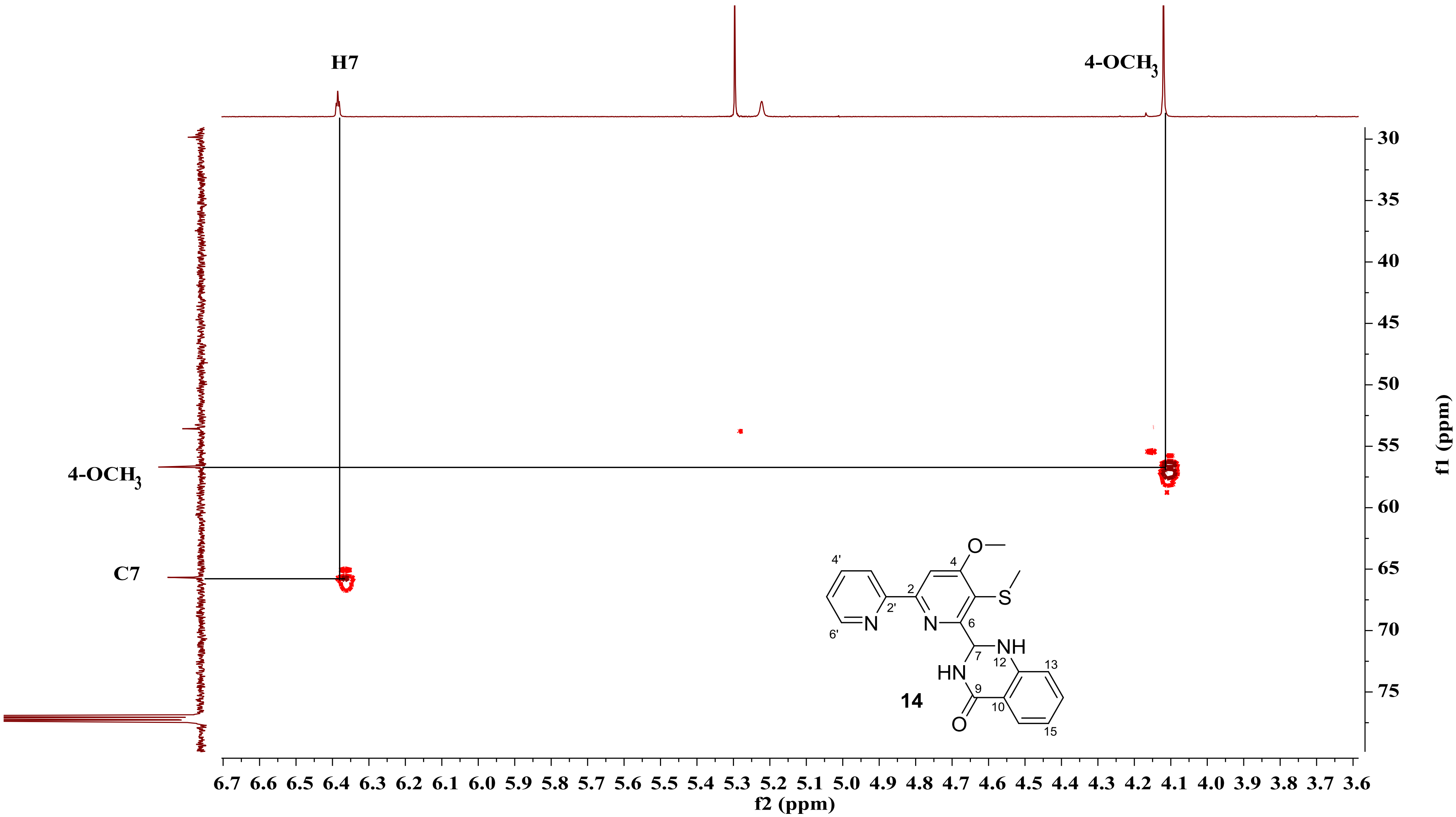

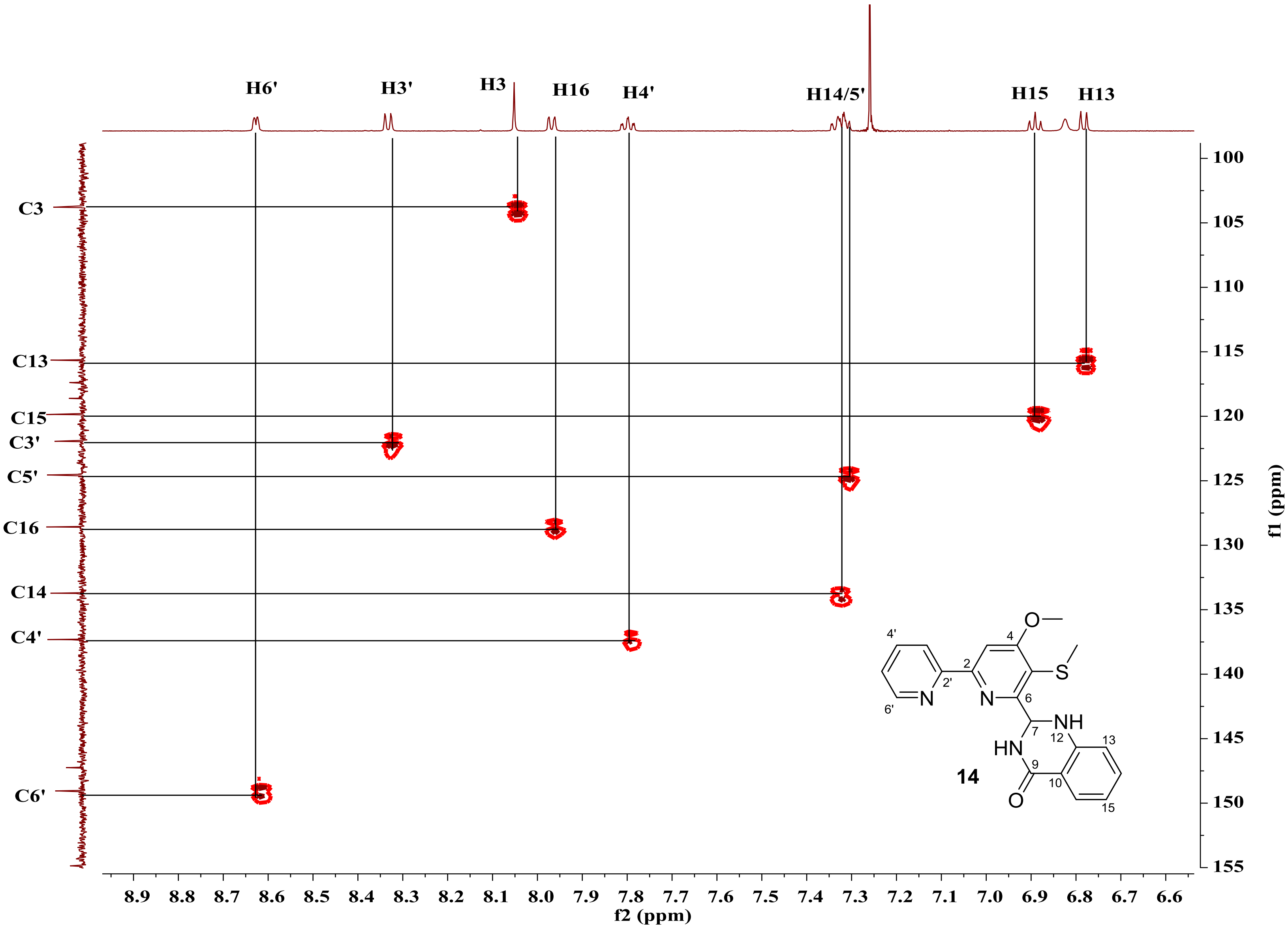
H6'
H3'
H3
H16
H4'
H14/5'
H15
H13
C3
C13
C15
C3'
C5'
C16
C14
C4'
C6'
14
f1 (ppm)
f2 (ppm)

Fig. S50. ^1H-^1H COSY spectrum of (±)-**14** in $CDCl_3$.

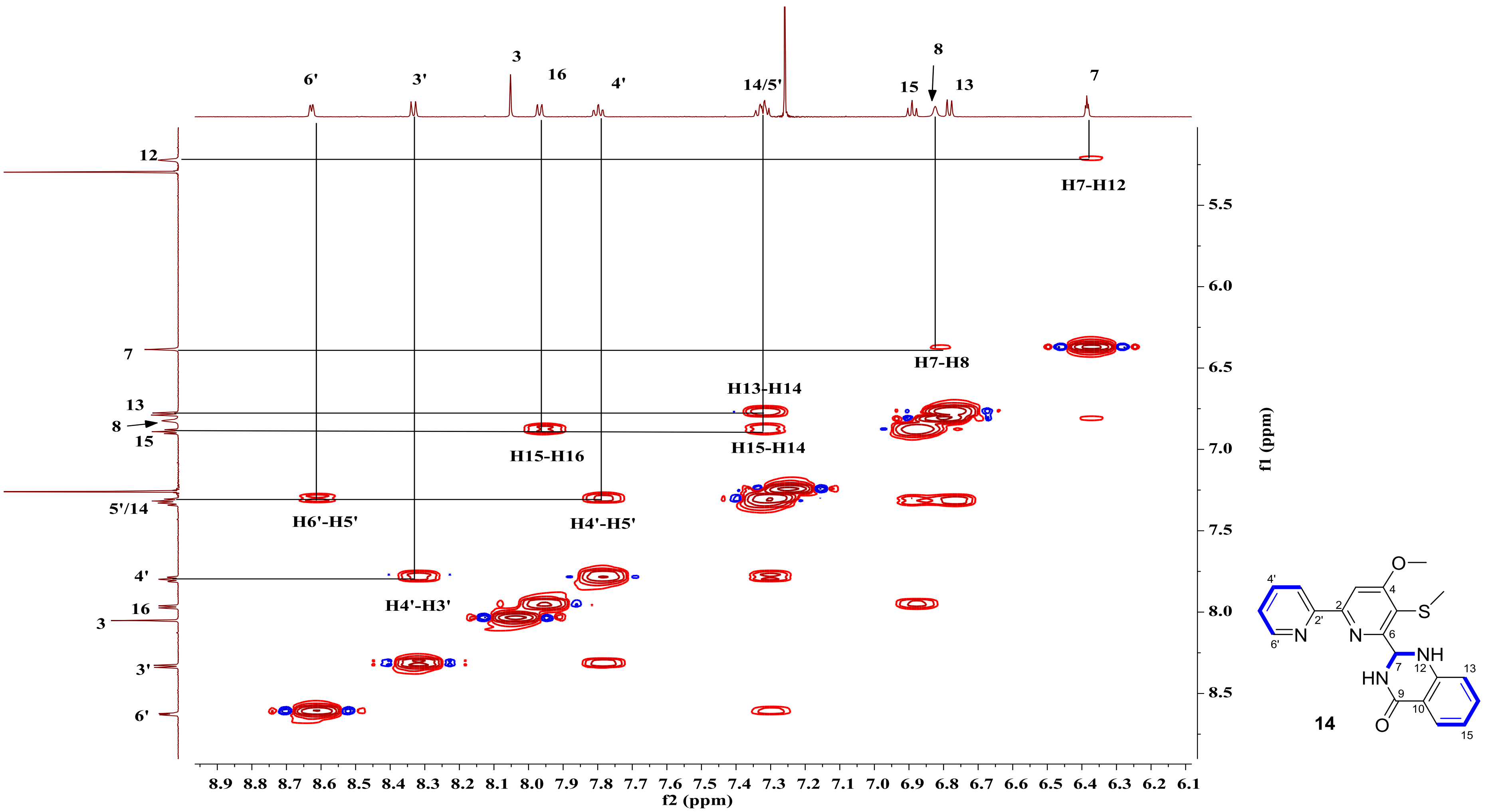

Fig. S51. HMBC spectrum of (±)-**14** in $CDCl_3$.

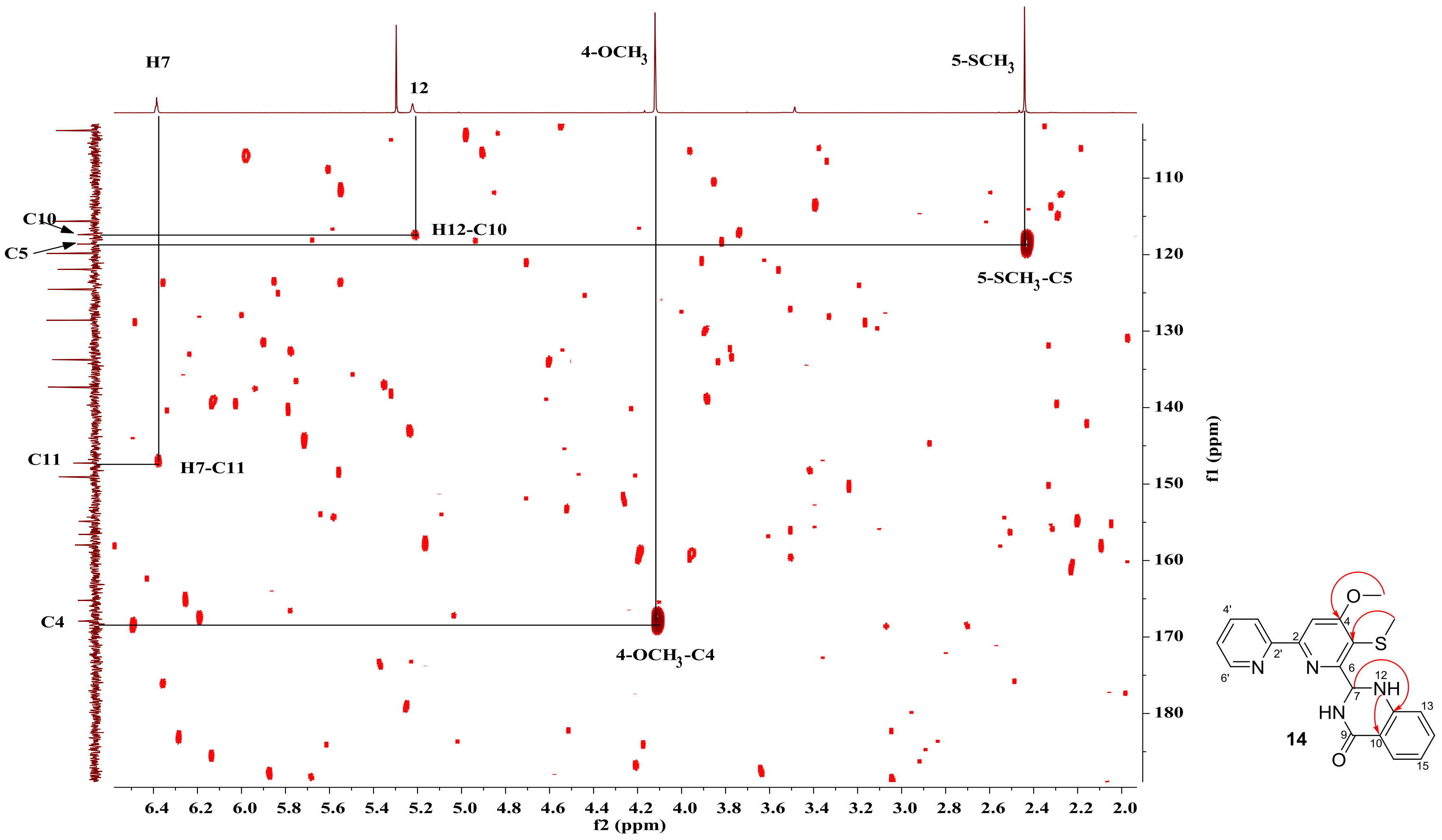

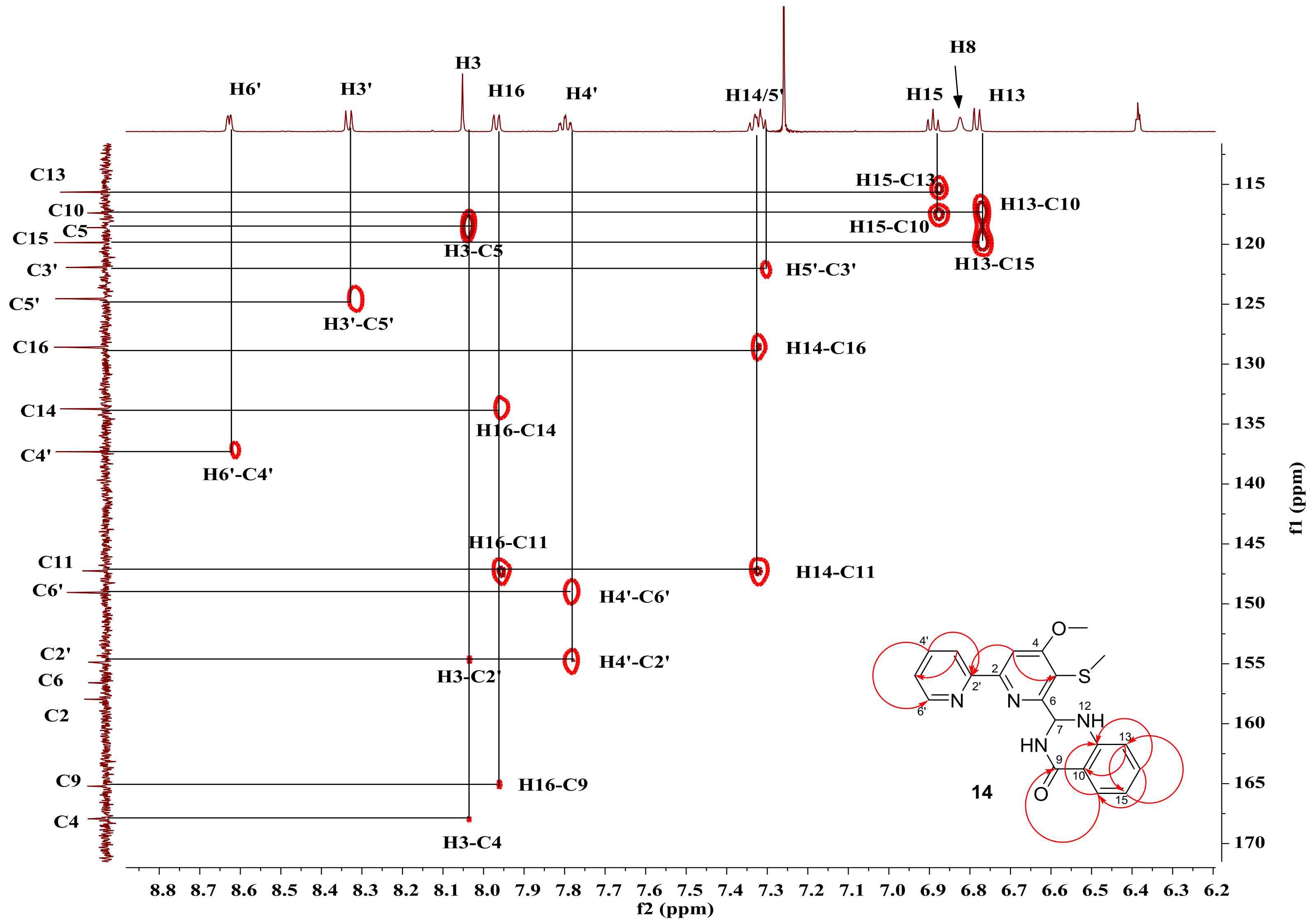

H6'
H3'
H3
H16
H4'
H14/5'
H8
H15
H13
C13
C10
C5
C15
C3'
C5'
C16
C14
C4'
C11
C6'
C2'
C6
C2
C9
C4
H15-C13
H13-C10
H15-C10
H3-C5
H13-C15
H5'-C3'
H3'-C5'
H14-C16
H16-C14
H6'-C4'
H16-C11
H14-C11
H4'-C6'
H4'-C2'
H3-C2'
H16-C9
H3-C4
f2 (ppm)
f1 (ppm)
14

Fig. S52. HPLC profile of (±)-pyrisulfoxin F ((±)-**14**) on chiral analytic column.

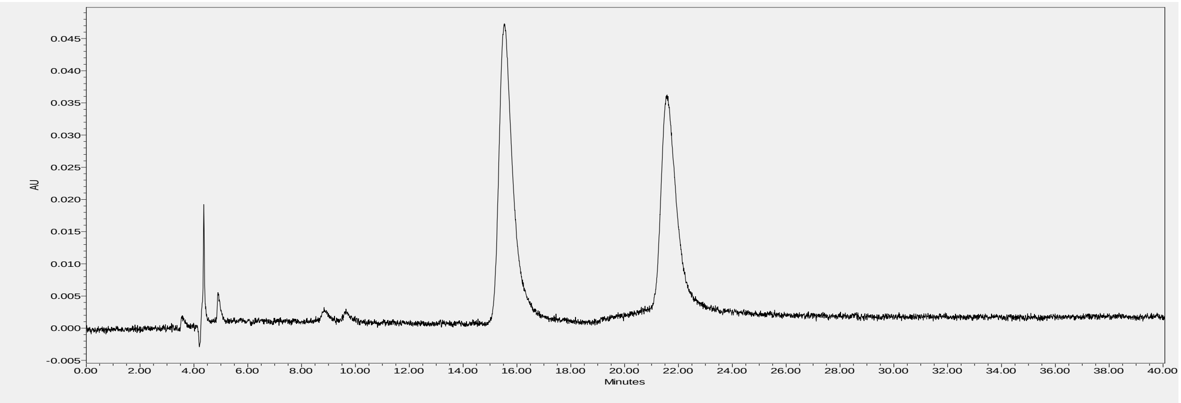

S(+)-**14**: t_R = 22.0 min; *R*(–)-**14**: t_R = 16.0 min (ChiralPak IA analytical column; 30% *i*-PrOH/*n*-C_6H_{14})

Fig. S53. UPLC-MS profile of the EtOAc extracts from solid cultivations of *Streptomyces albolongus* EA12432 at room temperature for 30 d (**a**) and 90 d (**b**), respectively (UPLC column: ACQUITY UPLC BEH C18, 2.1 × 50 mm, 1.7 μm; Solvents: MeCN–H_2O + 0.1% HCO_2H (0–1.5 min: 95%, 1.5–5 min: 95%–60%, 5–8 min: 60%–40%, 8–9 min: 40%–95%); Column temperature: 40 °C; flow rate: 0.4 mL/min; ESI^+ scan at 100–1500 Da, capillary voltage 0–2.5 kV, cone voltage 0–45 V).

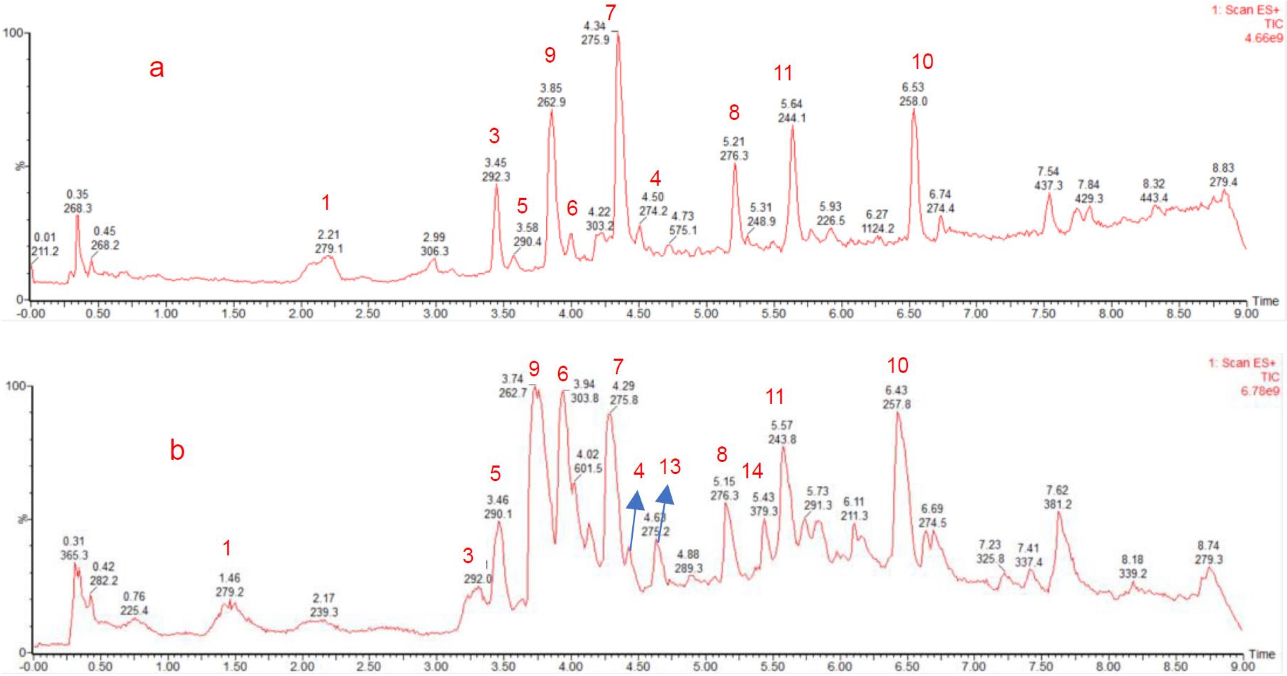

Supplement: Supplementary file 1 [file Data_Sheet_1.PDF]
